# Assessing Monoclonal and Polyclonal Antibodies in Sepsis and Septic Shock: A Systematic Review of Efficacy and Safety

**DOI:** 10.3390/ijms26188859

**Published:** 2025-09-11

**Authors:** Kyriakos Goulas, Martin Müller, Aristomenis K. Exadaktylos

**Affiliations:** 1 Department of Emergency Medicine, Inselspital University Hospital, University of Bern, 3010 Bern, Switzerland; martin.mueller@insel.ch (M.M.); aristomenis.exadaktylos@insel.ch (A.K.E.); 2 Department of Internal Medicine, Cantonal Hospital of Chur (Kantonsspital Graubünden), 7000 Chur, Switzerland

**Keywords:** sepsis, septic shock, monoclonal antibodies, polyclonal immunoglobulin, septicemia, precision immunotherapy, immunomodulation

## Abstract

This systematic review critically evaluates the efficacy and safety of monoclonal (mAb) and polyclonal (pAb) antibody therapies in adult sepsis and septic shock by synthesizing data from 29 randomized controlled trials (RCTs) encompassing over 10,000 patients. Sepsis and septic shock continue to be major critical-care mortality causes worldwide because of simultaneous hyperinflammatory and immunosuppressive responses. The clinical results from using targeted antibody therapies to manage this dysregulated response have shown inconsistent results. We conducted a comprehensive search of MEDLINE, Embase, Cochrane CENTRAL, Web of Science, and Google Scholar (through February 2025) to identify RCTs that compared mAb and pAb treatments to placebo or standard care in adult patients with sepsis or septic shock. Monoclonal antibodies against single cytokines e.g., Tumor Necrosis Factor-alpha (TNF-α) and endotoxin, did not significantly reduce 28-day mortality in unselected cohorts, though subgroup analyses of patients with elevated Interleukin-6 (IL-6) or early septic shock showed trends toward benefit. Intravenous Immunoglobulin (IVIG) enriched for Immunoglobulin M (IgM) demonstrated the most consistent mortality reduction when administered early in hyperinflammatory phases. Emerging precision strategies—including checkpoint inhibitors targeting Programmed Cell Death Protein 1/Programmed Death-Ligand 1 inhibitors (anti–PD-1/PD-L1), complement component 5a inhibitors (anti–C5a), and anti–adrenomedullin—were safe and improved organ-support-free days and Sequential Organ Failure Assessment (SOFA) scores. According to the Grading of Recommendations, Assessment, Development, and Evaluation (GRADE) approach, evidence showed moderate confidence for mortality, high certainty for safety and low to moderate certainty for secondary outcomes. The use of broad single-target monoclonal treatments has failed to deliver significant improvements in sepsis patient outcomes. The most promising approaches for sepsis treatment involve biomarker-guided precision strategies and polyclonal IgM-enriched IVIG. Future sepsis trials need to implement rapid immune profiling and adaptive designs and combination regimens to achieve optimal efficacy and establish personalized guideline-based sepsis management.

## 1. Introduction

Sepsis and septic shock remain major global health challenges characterized by unacceptably high incidence and mortality [1]. The World Health Organization estimates that in 2020, roughly 48.9 million people developed sepsis, with about 11.0 million sepsis-related deaths worldwide—roughly one in every five deaths globally [2,3]. These burdens are unevenly distributed: incidence and mortality are highest in low- and middle-income countries and sepsis disproportionately affects the very young, the elderly, and the chronically ill [4,5]. In high-resource Intensive Care Units (ICUs), septic shock mortality remains on the order of 30–50% and even in non-shock sepsis the in-hospital mortality rate often approaches 20–30% [1]. These figures underscore sepsis as a leading cause of mortality and morbidity, exceeding the combined deaths from many major cancers in some regions [1,3,6].

Beyond its devastating toll on human health, sepsis also imposes a profound economic burden on healthcare systems worldwide. The high incidence, complex management, and frequent need for intensive care translate into significant direct and indirect costs, making sepsis not only a clinical but also a major financial challenge for both high- and low-resource settings [1,7]. In high-income countries, the cost of sepsis care is substantial, with estimates ranging from USD 20,000 to USD 50,000 per patient episode [3,8,9]. In the United States, sepsis is one of the most expensive conditions treated in hospitals, accounting for over USD 20 billion in annual healthcare costs [9,10]. Similar high costs have been reported in Europe, with sepsis-related expenses ranging from EUR 7500 to EUR 27,000 per patient [1,8].

The pathogenesis of sepsis is complex and rooted in a dysregulated host immune response to infection [11]. Sepsis is formally defined (Sepsis-3 definition) as “life-threatening organ dysfunction caused by a dysregulated host response to infection” [12]. Normally, pathogen-associated molecular patterns (PAMPs) and damage-associated signals (DAMPs) are recognized by innate immune receptors including Toll-like receptors (TLRs) and Nucleotide-binding Oligomerization Domain-like receptors (NOD-like receptors), triggering intracellular signaling cascades such as the Mitogen-Activated Protein Kinase (MAPK) pathway and the Nuclear Factor kappa-light-chain-enhancer of activated B cells (NF-κB) pathway and producing pro-inflammatory cytokines [Tumor Necrosis Factor-alpha (TNF-α), Interleukin-1 beta (IL-1β), Interleukin-6 (IL-6), Interleukin-8 (IL-8), and interferons etc.] [13,14]. This initial inflammatory surge (often termed a cytokine storm) is aimed at controlling infection but can become excessive [15,16]. In sepsis, host defenses overshoot: pro-inflammatory and anti-inflammatory pathways become simultaneously activated in an uncontrolled fashion [16,17]. Early in sepsis, there is a massive secretion of cytokines and chemokines that promote vasodilation, increased vascular permeability, coagulation activation, and the recruitment of neutrophils and monocytes [15,16]. These events underlie hypotension, capillary leak, disseminated intravascular coagulation, and direct tissue injury [18]. Notably, this hyperinflammatory phase is not uniform nor self-limiting [19]. By the time patients present with severe sepsis or shock, both pro- and anti-inflammatory mediators are elevated concurrently [17,18,19]. Indeed, compensatory anti-inflammatory responses are activated nearly in parallel with pro-inflammatory responses, leading to features of immunosuppression [lymphocyte apoptosis, monocyte deactivation, regulatory T-cell expansion, upregulation of checkpoint molecules such as Programmed Cell Death Protein 1 (PD-1) and Cytotoxic T-Lymphocyte-Associated Protein 4 (CTLA-4), and elevated production of Interleukin-10 (IL-10)] even during the acute phase [16,17]. This simultaneous immune dysregulation is a hallmark of sepsis pathobiology [19].

The clinical consequences of this chaotic immune response are profound. Initially, the cytokine storm can precipitate multi-organ dysfunction, including acute respiratory distress syndrome (ARDS), acute kidney injury, liver dysfunction, and shock [20]. Subsequently, the immune system often lapses into a state of “immunoparalysis”, characterized by T-cell exhaustion, reduced Human Leukocyte Antigen (HLA-DR) expression on monocytes, and inability to clear primary infection or prevent secondary nosocomial infections [19,21]. Persistent inflammation, immunosuppression, and catabolism syndrome (PICS) is a recognized sequela that contributes to poor long-term outcomes in survivors [19,22]. In sum, sepsis pathophysiology involves an overzealous yet uncoordinated inflammatory response that paradoxically transitions into immune suppression [17,21], leaving patients vulnerable to ongoing organ injury and infection.

Current standard treatment for sepsis and septic shock is supportive and antimicrobial in nature. Early aggressive management with broad-spectrum antibiotics, source control, fluid resuscitation, vasopressors, and organ support (ventilation, renal replacement, etc.) constitutes the mainstay of care [12,23]. While these measures are essential, they do not directly modulate the pathological host response and, despite best efforts, mortality remains high [24]. Moreover, the empirical use of broad-spectrum antibiotics, although often life-saving, has contributed to rising antimicrobial resistance (AMR) worldwide [7,25]. Indeed, AMR was directly associated with 4.95 million deaths in 2019 and indirectly contributed to a larger number [25,26]. Multi-drug resistant (MDR) pathogens complicate sepsis care: infections with resistant organisms often require second-line or toxic antibiotics, are more difficult to eradicate and are correlated with higher mortality [25,27]. In short, limitations in current therapy—incomplete control of the dysregulated inflammation and the threat of AMR—underscore the urgent need for novel approaches to improve outcomes in sepsis [7].

Against this backdrop, adjunctive immunotherapeutic strategies have received renewed interest. The rationale is that targeting the host immune response (rather than only microbes) could restore balance and improve survival [28]. Historically, attempts at immune modulation in sepsis were met with disappointment; no targeted immunotherapy has yet been approved for sepsis. For example, trials of monoclonal antibodies against endotoxin (lipid-A of Gram-negative bacteria) in the 1990s, e.g., Human Anti–endotoxin-1A (HA-1A) and E5, failed to demonstrate clear benefit in unselected septic patients [29]. Likewise, anti–Tumor Necrosis Factor-alpha (anti–TNF-α) and anti–Interleukin-1 (anti–IL-1) therapies, which neutralize key cytokines in the early storm, showed limited efficacy and potential harm in broad sepsis populations [30]. A recombinant human activated protein-C (drotrecogin alfa) initially showed modest mortality benefit but was later withdrawn due to safety concerns [31,32]. Polyclonal immunoglobulin preparations including Intravenous Immunoglobulin (IVIG) with or without Immunoglobulin M (IgM) enrichment, have been evaluated in sepsis as well; however meta-analyses of IVIG treatment have yielded conflicting results [33]. Given these failures, new strategies emphasize patient selection (precision immunotherapy) and novel targets beyond classical cytokines [34].

Monoclonal antibodies (mAbs) operate via defined mechanisms. Those targeting pathogens or toxins directly neutralize virulence factors or promote opsonophagocytosis [35]. For instance, anti–alpha-hemolysin mAbs can block a key *Staphylococcus aureus* (*S. aureus*) toxin, whereas anti–endotoxin mAbs bind lipid-A and prevent lipopolysaccharide (LPS)–triggered Toll-like Receptor 4 (TLR-4) activation [35]. Antibodies against host factors (cytokines, receptors, and hormones) can tune the immune response [34,35]. Anti–cytokine mAbs e.g., anti–TNF-α and anti–Interleukin-6 Receptor (anti–IL-6R), aim to blunt harmful inflammation; anti–checkpoint mAbs, e.g., anti–PD-1 and anti–PD-L1 aim to restore T-cell function in immunosuppressed patients [35,36]. Antibodies can also target endothelial stabilizing molecules; e.g., enibarcimab binds adrenomedullin to preserve vascular integrity [35,37]. Each approach faces challenges as pathogen-targeted mAbs must match the infecting microbe (limiting broad applicability), while host-targeted mAbs risk suppressing necessary immune functions or missing the therapeutic window [38].

Polyclonal immunoglobulins provide a broad-acting alternative. IVIG contains a repertoire of antibodies that can neutralize multiple bacterial and viral antigens, enhance opsonization and modulate complement [39]. It also has Fc (crystallizable)—dependent anti-inflammatory effects (e.g., blocking Fc-γ receptors and scavenging complement) [39,40]. IgM-enriched IVIG may better target Gram-negative LPS and improve complement clearance [40,41]. Despite these theoretical benefits, clinical trials show variable efficacy. Due to the narrow therapeutic window, late administration risks immunoparalysis, whereas early use may provide limited benefit beyond standard treatment [40]. Moreover, IVIG does not address the underlying infection source or organ failure [33]. High cost and limited supply further constrain broad use [42].

In light of the escalating global burden of septic shock, exacerbated by AMR and the emergence of viral-triggered sepsis such as Severe Acute Respiratory Syndrome Coronavirus 2 (SARS-CoV-2), there is an urgent imperative to explore more precise therapeutic strategies. Monoclonal and polyclonal antibodies (pAbs), by virtue of their capacity to neutralize specific cytokines, pathogens, or immune checkpoints, offer a mechanistically grounded approach to rebalancing the host response [30,43,44,45]. This systematic review, encompassing all randomized controlled trials through February 2025, will rigorously evaluate the efficacy and safety of these biologics in septic shock, examining endpoints such as mortality, the duration of organ support, and the incidence of adverse events. In the existing literature, the systematic review by Mahdizade Ari et al. [43] remains the most directly comparable to our work by evaluating antibody-based therapies in neonatal, pediatric, and adult populations. While their expansive approach offers valuable safety and efficacy insights, combining diverse age groups and study designs can dilute the signal of adult-specific therapeutic effects. Accordingly, our review hones in on adult clinical trials of mAbs and pAbs, thereby delivering a sharper, more clinically relevant assessment of these interventions with an enhanced generalizability of findings in mature immune systems and critical-care settings. By synthesizing this evidence, we aim to clarify the therapeutic potential of antibody-based interventions, identify critical gaps in knowledge and inform the design of future precision-immunotherapy trials, ultimately advancing toward more individualized and effective sepsis management.

## 2. Methods

This systematic review aimed to address the research question: Are monoclonal and polyclonal antibodies effective and safe in the management of profound organ dysfunction due to sepsis and septic shock in hospitalized adults? The study focused on adult patients with sepsis or septic shock who had profound organ dysfunction to address a significant research gap about sepsis treatments since few systematic reviews studied antibody therapy safety and effectiveness. The approach bases its definitions on the Third International Consensus Definitions for Sepsis and Septic Shock (Sepsis-3) criteria but also accepts previous sepsis criteria used in earlier randomized controlled trials (RCTs) to maintain research and treatment development consistency [12]. The research protocol that was a priori constructed to address the research question based on the Population, Intervention, Comparator, Outcome (PICO) Framework [46] was published in the PROSPERO registry (ID: CRD420251073312).

### 2.1. Design and Search Strategy

A comprehensive search was conducted to address the clinical question regarding the effect of mAbs and pAbs (I) in hospitalized adult patients (P) with the diagnoses of sepsis or septic shock (Co). The search was performed across the following online databases: MEDLINE, Web of Science, Google Scholar, Embase, and the Cochrane Library (CENTRAL). The research protocol was designed and conducted according to the PRISMA-P 2015 (Preferred Reporting Items for Systematic Review and Meta-Analysis Protocols) [47] guidance and the full text was published first on PROSPERO registry. The extended PRISMA 2020 checklist was followed to maintain adherence to high reporting standards [47]. The research has been done both in “free text” and “subject headings” (Medical Subject Headings—MeSH or EMTree), and keywords were employed in the search strategy. The syntax encompassed the terms of Antibody (OR “Antibodies” OR “Monoclonal Antibodies” OR “Polyclonal Antibodies” OR “Ab” OR “Humanized Antibodies” OR “Bispecific Antibodies” OR “Antibody-Drug” OR “Immunoglobulin” OR “Immunoglobulins” OR “Immunoglobulin” OR “Immune Globulins” OR “Immunomodulatory”) and Sepsis (OR “Septic shock” OR “Septicemia” OR “Bacteraemia” OR “Bacteremia” OR “Bacteriemia” OR “Bacterial infections” OR “Bacterial infection” OR “Bacterial-infections” OR “Systemic Inflammatory Response Syndrome” OR “SIRS” OR “Endotoxemia” OR “Multiple Organ Failure” OR “Organ dysfunction” OR “Cytokine Release Syndrome”) and Treatment (OR “Therapy” OR “Therapeutic” OR “Care” OR “Intervention” OR “Effect” OR “Outcome” OR “Safety” OR “Management”) and RCT (OR “RCTs” OR “Randomized Controlled Trial” OR “Controlled Trial” OR “Controlled” OR “Trial” OR “blind” OR “Placebo”). In order to ensure comprehensive literature search citation tracking, both backward and forward searches were performed to capture any relevant studies not initially identified. To ensure the transparency and reproducibility of our findings, the complete search strategies—including full search strings for each database—are provided in Supplementary Materials S1.

### 2.2. Inclusion and Exclusion Criteria

Eligible studies were full-text, peer-reviewed articles published in English or German, with no time restrictions. Inclusion criteria were RCTs involving non-pregnant adult human subjects (≥18 years) diagnosed with organ dysfunction due to sepsis or septic shock. Sepsis severity was defined using clinical approaches, scoring systems—such as the Acute Physiology and Chronic Health Evaluation II (APACHE II), the Sequential Organ Failure Assessment (SOFA; score ≥ 2), or the National Early Warning Score 2 (NEWS2; score ≥ 7)—and laboratory biomarkers including procalcitonin (PCT), C-reactive protein (CRP), or lactate (≥2 mmol/L). Septic shock was defined according to the Sepsis-3 criteria [12], while inclusion criteria also incorporated the older, well-established definitions from the 2001 International Consensus Conference [48], as well as the original sepsis criteria proposed by Bone et al. in 1989 [49] and formalized in the 1992 American College of Chest Physicians/Society of Critical Care Medicine (ACCP/SCCM) consensus conference [50]. These earlier criteria are grounded in clear clinical and laboratory findings and remain comparable to Sepsis-3 in diagnostic rigor. Recommendations from the National Institute for Health and Care Excellence (NICE) guideline NG51 were also considered to maintain methodological consistency across studies [51]. Studies were required to evaluate mAbs and pAbs as an intervention and report relevant outcomes.

RCTs were excluded if they did not meet the inclusion criteria, involved animal or in vitro research, included pediatric populations, or were observational studies or clinical trials described as non-randomized. RCTs that failed to clearly define septic shock or organ dysfunction, did not report at least one of the following: mortality, survival outcomes, and safety, or did not compare the intervention against a placebo or standard treatment [standard sepsis management as per Surviving Sepsis Campaign (SSC) [52] or NG51 guidelines] [51] were also excluded. Additional exclusion criteria included studies with inconsistent or insufficient follow-up, those involving neutropenic (sepsis-associated immunosuppression was not an exclusion criteria) patients, or unpublished works such as abstracts or systematic reviews/meta-analyses. Importantly, the non-reporting of acute or late-onset adverse events was not a criterion for exclusion. The decision to exclude non-RCT studies is consistent with the study’s objective of ensuring the highest quality of clinical evidence.

### 2.3. Data Management

Data organization for this systematic review was independently conducted by each researcher using Zotero v7.0.15 (version 7.0.15, developed by Corporation for Digital Scholarship, Vienna, Virginia, USA), with each maintaining a separate library to ensure objectivity during data collection and organization. Retrieved articles were imported into Zotero v7.0.15, where bibliographic information, full texts, and notes were systematically organized. Studies were independently tagged according to the predefined inclusion criteria. Zotero v7.0.15 was further employed during manuscript preparation to manage citations effectively. Subsequently, the Systematic Review Data Repository (SRDR, developed by the Evidence-based Practice Center Program, Brown University) [53] platform was utilized as the next step to enhance data organization, storage, and tracking throughout the review process.

### 2.4. Study Selection

**Figure 1:**
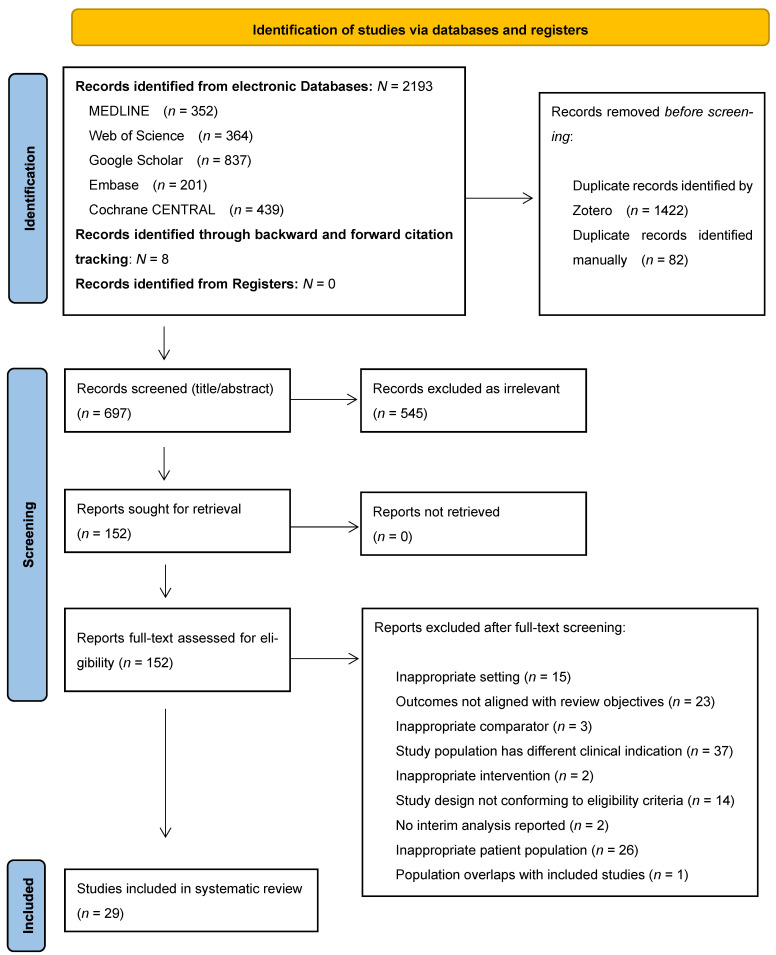
PRISMA flow diagram summarizing the study selection process.

Following the literature search, abstracts were independently reviewed by two evaluators (K.G. and A.K.E.) using predefined inclusion and exclusion criteria established in the study protocol. The abstracts, de-duplicated using Zotero v7.0.15, were assessed with a standardized evaluation form applying the PICO framework [46]. Discrepancies between the reviewers were resolved by a third reviewer (M.M.) to ensure consistency and eliminate bias. In the second stage, the full texts of selected abstracts underwent a detailed review to confirm eligibility, with each reviewer required to justify their decisions for inclusion or exclusion. In the second stage, the full texts of selected abstracts underwent a detailed review to confirm eligibility, with each reviewer required to justify their decisions for inclusion or exclusion. Reference lists of included studies were also screened to capture any additional relevant trials that may have been overlooked. The study selection process, adhering to systematic review standards, was visually summarized in a PRISMA flowchart (Figure 1) to ensure methodological rigor and transparency.

### 2.5. Data Extraction

Data were extracted by two independent reviewers (K.G. and A.K.E.) using the SRDR platform [53], ensuring consistency and accuracy in capturing relevant information across all included studies. Any disagreements were resolved through structured discussion and if necessary, third-party expert consultation (M.M.) was sought. Data extracted included study identification (author, publication year, country, and study design), population characteristics (sample size, age, sex, ethnicity/race, and comorbidities), the definition of sepsis and cause of sepsis, study structure (the number of study arms, follow-up duration, and subgroup analysis), specifics of the antibodies used (type, dosage, administration routes, and the duration of treatment), and study outcomes (mortality rates, prognostic scores, clinical/laboratory findings, and adverse events).

### 2.6. Study Outcomes

The primary outcome of this study was efficacy, defined as all-cause mortality at the end of the follow-up period, along with a comparison of survival rates between the two study arms (mAbs or pAbs administration versus placebo or standard sepsis management)

The study measured laboratory changes through hematological, biochemical, immunological, and microbiological analyses as its secondary outcomes. The study evaluated the tolerability of mAb and pAb as well as multiple efficacy endpoints which included health service utilization metrics such as hospital stay duration and ventilator-free days and ICU-free days and shock-free days. The study evaluated SOFA scores to assess sepsis-related multi-organ dysfunction and severity of illness indices. The study evaluated the incidence and severity of adverse events which were defined as any side effects related to treatment in both treatment and control groups. The study evaluated acute and late-onset complications from mAb and pAb therapies together with their immunomodulatory effects as secondary outcomes.

### 2.7. Quality Assessment

**Figure 2:**
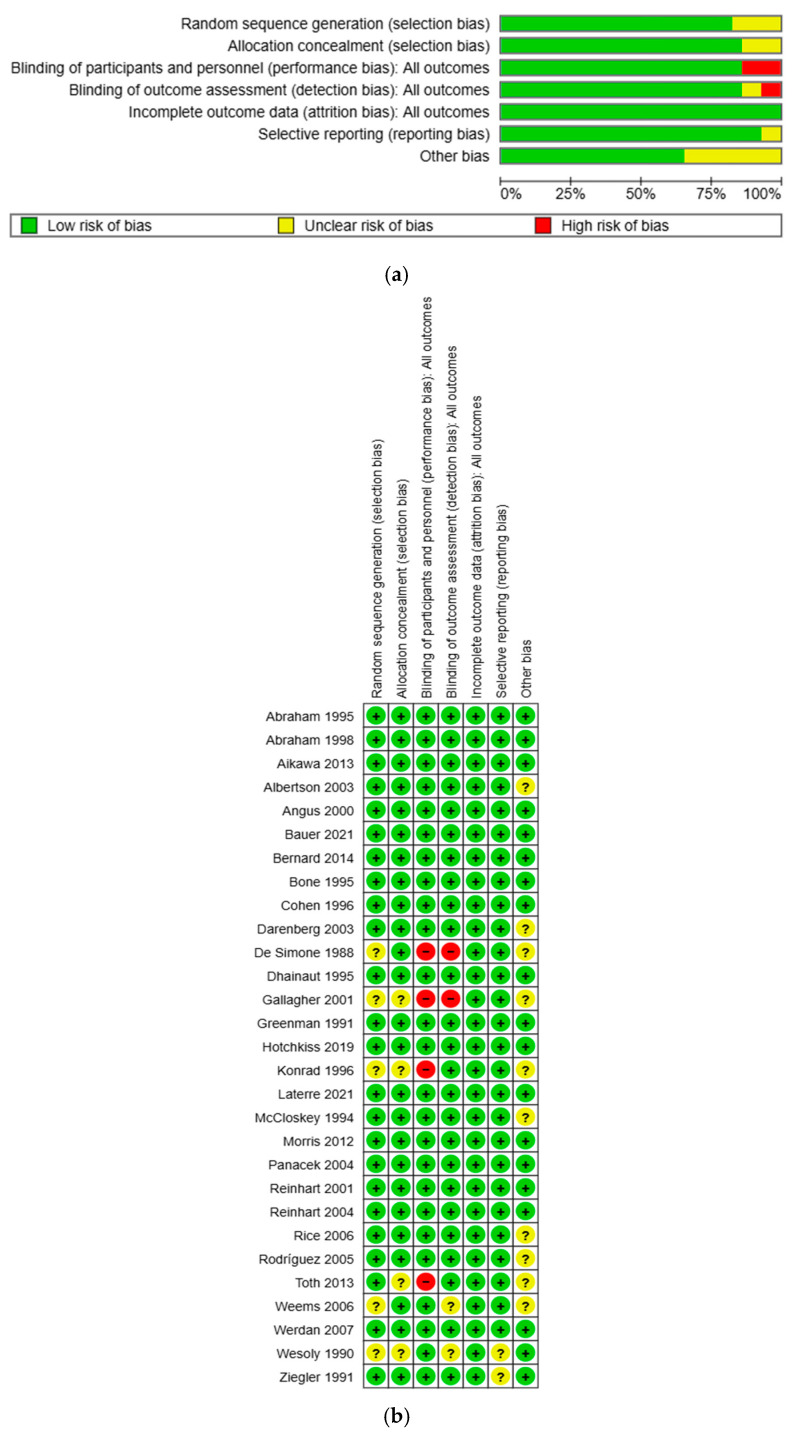
Risk of bias assessment [57,58,59,60,61,62,63,64,65,66,67,68,69,70,71,72,73,74,75,76,77,78,79,80,81,82,83,84,85]: (**a**) Summary of RoB 2 domain-level judgments across all included studies; (**b**) study-specific RoB 2 assessments for each individual trial. In both panels, green shading denotes “low risk,” yellow denotes “some concerns,” and red denotes “high risk” of bias.

The methodological quality of the included randomized controlled trials was evaluated using the Cochrane Risk of Bias 2.0 (RoB-2) tool (Cochrane Collaboration, Oxford, UK) [54]. This structured assessment examined six key domains: random sequence generation, allocation concealment, the blinding of participants and personnel, the blinding of outcome assessment, incomplete outcome data, selective outcome reporting, and other potential sources of bias. Each domain was rated as indicating a low, high, or unclear risk of bias. The assessment was independently conducted by two reviewers (K.G. and A.K.E.), with discrepancies resolved by a third reviewer (M.M.) to ensure objectivity and consensus. The risk-of-bias results were synthesized graphically using Review Manager (RevMan Web, developed by the Cochrane Collaboration, Oxford, UK) and are presented in Figure 1 and Figure 2.

Studies were classified as “high risk” if any individual domain was judged to be at high risk of bias, or if two or more domains were assessed as raising some concerns. These criteria were established to identify studies whose methodological limitations could significantly compromise the validity of effect estimates. The risk of bias assessment served as the foundation for both the qualitative interpretation of individual study findings and for subsequent quantitative analyses.

In accordance with PRISMA guidelines, a sensitivity analysis was conducted to explore the impact of excluding high-risk studies on the overall conclusions. This analysis aimed to assess the robustness of pooled estimates and to determine whether lower-quality studies disproportionately influenced effect size or heterogeneity.

Additionally, the Grading of Recommendations, Assessment, Development, and Evaluations (GRADE) framework was employed to appraise the certainty of the evidence across key outcomes, including all-cause mortality, organ dysfunction scores, inflammatory biomarkers, and safety endpoints [55]. The GRADE approach incorporated the risk of bias, inconsistency, indirectness, imprecision, and publication bias in rating the overall certainty of evidence for this systematic review. This dual-layered approach of Risk of Bias 2.0 (RoB 2.0) and GRADE ensured a rigorous and transparent evaluation of both the internal validity and the strength of the evidence base informing this systematic review.

To critically appraise our systematic review’s methodological rigor, we applied the AMSTAR-2 tool (A MeaSurement Tool to Assess systematic Reviews 2), encompassing 16 key domains including protocol registration, literature search and selection, data extraction, risk-of-bias evaluation, and synthesis methodology [56]. Two independent reviewers (K.G. and A.K.E.) completed the assessment for each domain. In the event of disagreement, a third reviewer (M.M.) adjudicated to reach consensus. The detailed, item-level AMSTAR-2 ratings for this review are provided in Supplementary Materials S2.

## 3. Results

### 3.1. Search Results and Characteristics of Included Studies

The systematic literature search yielded a total of 2193 records identified from electronic databases including MEDLINE (*n* = 352), Web of Science (*n* = 364), Google Scholar (*n* = 837), Embase (*n* = 201), and Cochrane CENTRAL (*n* = 439). An additional eight records were identified through backward and forward citation tracking. No records were identified from registers. Following the PRISMA guidelines [47], 1422 duplicate records were removed through automatic deduplication using Zotero Version 7.0.15, and an additional 82 duplicates were identified manually, resulting in 697 records for title and abstract screening. Of these, 545 records were excluded as irrelevant to the research question. The remaining 152 reports were retrieved for full-text assessment. After detailed full-text evaluation, 123 articles were excluded for the following reasons: inappropriate setting (*n* = 15), outcomes not aligned with review objectives (*n* = 23), inappropriate comparator (*n* = 3), study population with different clinical indication (*n* = 37), inappropriate intervention (*n* = 2), study design not conforming to eligibility criteria (*n* = 14), no interim analysis reported (*n* = 2), inappropriate patient population (*n* = 26), and population overlap with included studies (*n* = 1). A comprehensive list of studies excluded during full-text screening, along with the specific reasons for exclusion, is presented in Supplementary Materials S3. This rigorous screening process resulted in 29 studies that met all inclusion criteria and were included in the final analysis.

Following selection, a descriptive overview of the study populations and therapeutic targets was conducted. A total of 10,332 adult (≥18 yrs) participants were enrolled across the included studies, with 57% male and 43% female (5889 males vs. 4443 females). Table 1 summarizes the characteristics of these RCTs, all of which evaluated the effects of mAbs and pAbs in patients with sepsis or septic shock. Most studies were conducted between 1990 and 2010 (each year averaging 1.2 RCTs). The United States accounted for the highest number of trials, with 14 studies conducted there (Abraham et al., 1995 [57]; Abraham et al., 1998 [58]; Albertson et al., 2003 [59]; Angus et al., 2000 [60]; Bone et al., 1995 [61]; Gallagher et al., 2001 [62]; Greenman et al., 1991 [63]; Hotchkiss et al., 2019 [64]; McCloskey et al., 1994 [65]; Morris et al., 2012 [66]; Panacek et al., 2004 [67]; Rice et al., 2006 [68]; Weems et al., 2006 [69]; Ziegler et al., 1991 [70]).

The analysis of 29 RCTs investigating antibody-based therapies for sepsis and septic shock revealed considerable heterogeneity in study design, intervention types, molecular targets, and definitions of sepsis and septic shock, as well as the outcome measurements used to evaluate the clinical effectiveness of the interventions. All included studies were prospective, parallel-group RCTs, with the majority (83%) employing a double-blind, placebo-controlled methodology. The remaining trials were conducted as either open-label or single-blind phase I/II safety studies. Placebo comparators typically consisted of human serum albumin or saline, providing a consistent control across studies. Uniformly, all antibody products were administered via the intravenous route within the first 24 h after patients met the inclusion criteria of each study and were diagnosed with sepsis or septic shock.

Among these trials, 69% of studies used mAbs and 31% used pAbs, respectively. There was significant diversity in the types of antibodies investigated, including various anti–TNF-α mAbs (Abraham et al., 1995 [57]; Abraham et al., 1998 [58]; Aikawa et al., 2013 [71]; Bernard et al., 2014 [72]; Cohen et al., 1996 [73]; Dhainaut et al., 1995 [74]; Gallagher et al., 2001 [62]; Konrad et al., 1996 [75]; Morris et al., 2012 [66]; Panacek et al., 2004 [67]; Reinhart et al., 2001 [76]; Rice et al., 2006 [68]), anti–endotoxin antibodies (Albertson et al., 2003 [59]; Angus et al., 2000 [60]; Bone et al., 1995 [61]; Greenman et al., 1991 [63]; McCloskey et al., 1994 [65]; Ziegler et al., 1991 [70]), anti–complement antibodies (Bauer et al., 2021 [77]), anti–PD-L1 antibodies (Hotchkiss et al., 2019 [64]), anti–CD14 antibodies (Reinhart et al., 2004 [78]), anti–adrenomedullin antibodies (Laterre et al., 2021 [79]), anti–clumping factor A (Weems et al., 2006 [69]) and polyclonal immunoglobulin preparations (Darenberg et al., 2003 [80]; DeSimone et al., 1988 [81]; Rodríguez et al., 2005 [82]; Toth et al., 2013 [83]; Werdan et al., 2007 [84]; Wesoly et al., 1990 [85]).

The definitions of sepsis and septic shock also varied considerably across studies, reflecting the evolution of sepsis criteria over time. The majority of studies have employed sepsis definitions originating from the Bone et al. 1992 ACCP/SCCM Consensus [50], as well as various adaptations of these consensus criteria, while more recent studies incorporated Sepsis-3 criteria [12] (Bauer et al., 2021 [77]; Laterre et al., 2021 [79]).

Another key aspect in which substantial variability was demonstrated among the included studies concerns the outcome measurements used to assess clinical effectiveness. Most studies selected all-cause mortality as the primary efficacy endpoint, yet the timing of mortality assessment varied substantially, with mortality reported at 7, 14, 28, and 90 days, and in some instances, as late as 180 days. Secondary clinical outcomes also varied widely. Many studies incorporated organ dysfunction scores, such as SOFA score and the APACHE II, to quantify the severity and progression of multi-organ dysfunction and to assess changes in baseline illness severity. Additional secondary endpoints included shock resolution times and vasopressor-free days, which served as indicators of hemodynamic stabilization. Functional recovery and resource utilization were further captured through measurements such as ventilator-free days, ICU-free days and shock-free days, providing a broader perspective on patient recovery trajectories and the burden on critical care resources. Laboratory and biomarker assessments constituted another layer of heterogeneity. Some trials measured inflammatory cytokines like TNF-α and IL-6, biomarkers of endothelial function and organ injury, as well as microbiological parameters such as pathogen clearance and endotoxin levels. The selection and timing of these laboratory endpoints varied, limiting comparability across studies. Safety and tolerability were systematically evaluated in all trials, with adverse events, serious adverse events, immunologic reactions, and infusion-related complications being reported.

### 3.2. Mortality Outcomes Following Monoclonal Antibody Intervention

The analysis of mortality outcomes was stratified by the target of the mAb intervention.

#### 3.2.1. Anti–TNF-α Monoclonal Antibodies

In the earliest large-scale evaluation, Abraham et al. [57,58] conducted two multicenter trials of murine IgG1 anti–TNF-α mAb in patients with suspected infection and organ dysfunction. The 1995 study randomized 994 patients to receive a single intravenous infusion of 7.5 mg/kg or 15 mg/kg antibody versus placebo; although early (day 3) mortality was lower in treated patients, 28-day all-cause mortality did not differ significantly, with rates of 29.5% and 31.3% versus 33.1%, respectively, and a non-significant trend toward benefit in the shock subgroup (37.7% and 37.8% vs. 45.6%). In 1998, a larger cohort of 1879 septic shock patients received the same intervention, yielding 40.3% mortality in the antibody arm versus 42.8% with placebo (*p* = 0.27), with no difference in shock reversal or subgroup analyses.

Cohen et al. [73] extended these findings in a 14-country trial of 564 patients, administering single doses of 3 mg/kg or 15 mg/kg murine anti–TNF-α mAb within 16 h of sepsis onset. Neither dose conferred a survival benefit at 28 days—mortality was 31.5% and 42.4% versus 39.5% with placebo—although treated patients experienced improved shock reversal and delayed organ failure.

**Table 1:** Data extracted from included RCTs.

Author, Year, Country	Study Design	Investigational Drug	Sample Size, Demographics	Study Arms, Follow-Up	Stratification Subgroup Analysis	Sepsis Criteria	Outcomes	Drug Administration	Results	Risk of Bias
Abraham et al., 1995, (USA, Canada) [57]	RCT, multicenter, double-blind, placebo-controlled, prospective	Anti-TNF-α mAb (murine IgG1)	*N* = 994 enrolled; 971 infused. Mean age ~59 yrs. Male: ~56%. Balanced arms. Similar APACHE II (~25).	3 arms: TNF-α mAb in two different doses vs. placebo. Follow-up: 28 days.	Stratified: Shock vs. non-shock pre-randomization. Subgroups: Shock status, APACHE II, infection type (GPB/GNB).	Based on Bone et al. Study (1989) [49]: Suspected infection and SIRS and organ dysfunction.	Primary: 28-day all-cause mortality. Secondary: Mortality at days 3, 7, and 14, AEs, HAMA titers, labs, vitals, APACHE II changes.	Single IV infusion (7.5 or 15 mg/kg TNF-α mAb) within 4 h post-randomization.	28-day all-cause mortality: TNF-α mAb 15 mg/kg 31.3%, 7.5 mg/kg 29.5%, placebo 33.1% (NS). In shock subgroup: Trend to reduced mortality (15 mg/kg 37.7%, 7.5 mg/kg 37.8%, placebo 45.6%, *p* = 0.15–0.20). Early mortality at day 3: Significant reduction in TNF-α mAb. Late organ failure reversal: NS. Safety: AEs similar across groups (~4.6%).	Low
Abraham et al., 1998, (USA, Canada) [58]	RCT, multicenter, double-blind, placebo-controlled, prospective	Anti-TNF-α mAb (murine IgG1)	*N* = 1879 septic shock patients: 948 TNF-α mAb, 925 placebo. Mean age: ~59 yrs. Male: 60.5%. White: ~65%. Mean APACHE II: 28.4 (TNF-α mAb) vs. 28.8 (placebo).	2 arms: TNF-α mAb vs. placebo. Follow-up: 28 days.	Stratified by center. Subgroups: Baseline IL-6 (>1000 pg/mL), detectable TNF-α, shock duration, infection type (GPB/GNB).	Septic shock ≤ 12 h onset; SIRS + organ dysfunction (hypotension refractory to fluids, altered mental status, hypoxemia, metabolic acidosis, oliguria, DIC) per Bone 1989 [49] adaptations.	Primary: 28-day all-cause mortality. Secondary: 7- and 14-day mortality, shock reversal, prevention of new shock/organ failure, coagulopathy rates, AEs, anti-TNF-α mAb antibodies, tolerability.	Single IV infusion (TNF-α mAb 7.5 mg/kg or placebo—0.25% human serum albumin) over ~30 min, within 4 h of randomization.	28-day mortality: 40.3% (TNF-α mAb) vs. 42.8% (placebo) (*p* = 0.27). Shock reversal/duration or prevention: NS difference. Coagulopathy ↓ with TNF-α mAb at day 7 (*p* < 0.001), day 28 (*p* = 0.005). Cytokines: No survival benefit baseline IL-6 or TNF-α. Safety: AEs similar, well tolerated; no anti-TNF-α mAb antibodies detected.	Low
Aikawa et al., 2013, (Japan) [71]	RCT, multicenter, phase II, double-blind, placebo-controlled	AZD9773 Ovine polyclonal Fab frags of IgG (anti-TNF-α)	*N* = 20 sepsis patients: AZD9773 cohort 1 (*n* = 7), cohort 2 (*n* = 7), placebo (*n* = 6). Mean age: 75 yrs; Male: ~45%. APACHE II mean ~26; SOFA mean ~10.	3 arms: Low-dose and high-dose AZD9773 vs. placebo. Follow-up: To day 29.	NR.	Infection + ≥2 SIRS criteria (incl. temp or WBC) + cardiovascular and/or respiratory failure per Bone 1992 [50].	Primary: Safety/tolerability PK/PD. Secondary: Exploratory outcomes (SOFA, VFDs, ICU-free days, infection rates, mortality at day 29).	IV infusion: Cohort 1: 250 U/kg loading, then 50 U/kg q12h × 9 doses. Cohort 2: 500 U/kg loading, then 100 U/kg q12h × 9 doses or placebo.	29-day all-cause mortality: AZD9773 cohort 1 (low dose): 14.3% (1/7), AZD9773 cohort 2 (high dose): 28.6% (2/7), placebo: 33.3% (2/6). SOFA and organ failure resolution: Similar. Cytokines: TNF-α, IL-6, IL-8 ↓ more in AZD9773 arms. Safety: No treatment discontinuations due to AEs.	Low
Albertson et al., 2003, (USA) [59]	RCT, multicenter, double-blind, placebo-controlled, prospective	MAB-T88 Human IgM mAb	*N* = 826 enrolled (411 MAB-T88, 415 placebo). Mean age: ~57 yrs. Male: ~60%. APACHE II mean: ~26.8.	2 arms: MAB-T88 vs. placebo. Follow-up: 28 days.	Stratified by shock presence, age, APACHE II, isolated UTI. Subgroups: documented ECA infection, bacteremia.	Temperature: ≥35.5 °C or ≥38.3 °C, tachycardia: ≥90 bpm (no β-blockers), tachypnea: ≥20 breaths/min or mech. vent., hypotension, or dysfunction of ≥2 end-organs, presumptive G- infection (culture/stain).	Primary: 28-day survival. Secondary: AEs, organ dysfunction, lab parameters.	Single IV infusion over 30 min: 300 mg MAB-T88 or placebo (albumin).	28-day all-cause mortality: MAB-T88 (34.2%) vs. placebo (30.8%) in ECA group, *p* = 0.44. All 826 patients: MAB-T88 (37.0%) vs. placebo (34.0%), *p* = 0.36. Safety: More AEs in MAB-T88 group (*p* < 0.05).	Low
Angus et al., 2000, (USA) [60]	RCT, multicenter, phase III, double-blind, placebo-controlled, prospective	E5 Murine mAb IgM	*N* = 1102 randomized; 1090 treated (550 E5, 552 placebo). Mean age: 60 yrs, Male: 55%. Hypotension: ~75%. Organ dysfunction in 84%. Shock at presentation: 46%.	2 arms: E5 vs. placebo Follow-up: 28 days.	Stratified by shock presence. Subgroups pre-specified by shock status, comorbidities, organ failure, infection site, organism.	Severe sepsis per ACCP/SCCM 1992 [50]: SIRS + hypotension or hypoperfusion + organ dysfunction; Gram-negative infection documented or probable.	Primary: 14-day all-cause mortality. Secondary: 28-day mortality, subgroup mortality (shock status), organ dysfunction resolution, lab abnormalities, AEs, and withdrawal due to AEs.	IV infusion: E5 2 mg/kg IV infusion over 1 h × 2 doses (24 h apart) vs. placebo.	14-day mortality: E5 29.7% vs. placebo 31.1% (*p* = 0.67). 28-day mortality: E5 38.5% vs. placebo 40.3% (*p* = 0.56). NS difference in any subgroup analysis. AEs: Similar frequency and profile.	Low
Bauer et al., 2021 (Germany) [77]	RCT, multicenter, phase IIa, double-blind, placebo-controlled, prospective	Vilobelimab (IFX-1) recombinant mAb anti-C5a	*N* = 72 patients with severe sepsis/septic shock. Mean age: 63 yrs (vilobelimab) vs. 64 yrs (placebo). Male: 61% (vilobelimab) vs. 67% (placebo). Baseline APACHE II median: ~17.5–22.5. SOFA median: ~8.5–9.0	4 arms: Vilobelimab, three cohorts, vs. placebo. Follow-up: 28 days.	Stratified by infection focus (abdominal or pulmonary). Subgroups post hoc combining cohorts 2 + 3.	Sepsis-3 definition [12]. Infection-related organ dysfunction onset < 6 h or vasopressor therapy < 3 h.	Co-primary: PD (C5a levels), PK (vilobelimab levels), safety/tolerability. Secondary: 28d all-cause mortality (ACM), SOFA, ICU-/ventilator-/vasopressor-/RRT-free days, cytokines (IL-6, IL-8, IL-10), antibiotic days.	IV infusion: Vilobelimab cohort 1: 2 × 2 mg/kg IV (0, 12 h); cohort 2: 2 × 4 mg/kg IV (0, 24 h); cohort 3: 3 × 4 mg/kg IV (0, 24, 72 h); placebo matching volumes/times.	28-day all-cause mortality: Placebo 12.5%, cohort 1 37.5%, cohort 2 18.8%, cohort 3 12.5%. Critical care: Cohorts 2 + 3 had more ICU- and ventilator-free days. Cytokines: Vilobelimab led to dose-dependent ↓C5a; no impact on MAC formation. SOFA scores: No difference in SOFA, vasopressor-, RRT-free days. Safety: AEs frequent but similar across groups.	Low
Bernard et al., 2014, (France, Belgium, Canada, Australia, Czech Republic, Finland, Spain) [72]	RCT, multicenter, phase IIb, double-blind, placebo-controlled	AZD9773 Ovine polyclonal Fab frags of IgG (anti-TNF-α)	*N* = 300 patients with severe sepsis/septic shock. *N* = 296 treated (100/arm: low-/high-dose AZD9773, placebo). Median age: 62. Male: 66%. APACHE II: mean 25.2; baseline SOFA: mean 9.0.	3 arms: Low-dose AZD9773 and high-dose AZD9773 vs. placebo Follow-up: To day 90.	Stratified by APACHE II, age, region, mech. ventilation. Subgroups: baseline TNF-α quartiles, infection site, organ failures, gender, age.	Severe sepsis/septic shock: Infection + ≥2 SIRS criteria (incl. temp/WBC) + cardiovascular and/or respiratory failure (Bone 1989 criteria) [49].	Primary: Mean VFDs to day 29. Secondary: 7-, 29-, and 90-day mortality, time to death, shock-free days, organ-failure-free, ICU-free days (D15), SOFA score, infection relapse, PD, AEs.	IV infusion; loading dose + maintenance q12h for 5 days (max 10 doses); dose capped at 100 kg body weight.	29 d mortality: Placebo 20%, low-dose 15%, high-dose 27% (NS). VFDs: Low-dose 19.7, high-dose 17.3, placebo 18.3 (NS). SOFA score: Similar improvement across groups. Infection relapse and ICU/organ-failure-free days: NS. Safety: TNF-α significantly reduced (*p* < 0.001); no effect on IL-6/IL-8; AEs similar across groups; no safety signals; no subgroup showed clear benefit.	Low
Bone et al., 1995, (USA) [61]	RCT, multicenter, double-blind, placebo-controlled, prospective	E5 Murine mAb IgM	*N* = 847 randomized, *N* = 830 treated, *N* = 811 analyzed. Balanced gender distribution and similar APACHE II scores.	2 arms: E5 vs. placebo. Follow-up: 30 days.	Stratified by documented GN sepsis, organ failure presence, bacteremia status. Subgroups: On organ failure resolution, mortality in subsets.	Known/suspected G- infection: Clinical sepsis (≥2 SIRS criteria). Organ dysfunction (renal, respiratory, coagulation, CNS, hepatic). Exclusion: Refractory shock.	Primary: 30-day all-cause mortality. Secondary: Resolution/prevention of organ failures, AEs, (HAMA) development, length of hospitalization.	IV infusion, E5 2 mg/kg per dose, 2 doses ~24 h apart, 1 h infusion each or placebo.	30-day mortality: 30% E5 vs. 33% placebo, (*p* = 0.21). Organ failure resolution: Improved with E5 (48% vs. 25%, *p* = 0.005). All patients with organ failure: 41% (E5) vs. 27% (placebo) (*p* = 0.024). Prevention of new organ failures: ARDS 5% (E5) vs. 12% (placebo) (*p* = 0.007). CNS Dysfunction 4% (E5) vs. 10% (placebo) (*p* = 0.05). Safety: Hypersensitivity reactions: 2.6% (E5), HAMA development: 44% (E5) vs. 12% (placebo).	Low
Cohen et al., 1996, (14 countries) [73]	RCT, multicenter, double-blind, placebo-controlled, prospective	BAY 1351 Murine anti-TNF-α mAb	*N* = 564 enrolled, 553 infused. 420 in shock for primary analysis. Male: ~60%. APACHE II ~22 (mean).	3 arms: BAY 1351 vs. placebo. Follow-up: 28 days.	Stratification by shock vs. non-shock. Subgroups by infection type, APACHE II score.	SIRS + organ hypoperfusion/dysfunction per Bone 1989 [49] criteria (infection, temp, HR, RR, organ failure).	Primary: 28-day all-cause mortality rate. Secondary: Shock reversal and frequency of organ failures, AEs.	IV infusion over 30 min; single dose of BAY 1351 15 or 3 mg/kg within 16 h of sepsis onset or placebo (human albumin).	28 d mortality: 3 mg/kg 31.5% vs. placebo 39.5% (NS); 15 mg/kg 42.4% (NS). Shock subgroup: 3 mg/kg 36.7% vs. placebo 42.9% (NS). Organ failure resolution: Improved shock reversal, delayed organ failure in treatment groups (*p* = 0.007, *p* = 0.01), organ failure rates: 15-mg/kg 40.2%, 3-mg/kg 44.3%, placebo 59.2% (*p* = 0.03, *p* = 0.06). Safety: AEs similar; high anti-mouse antibody development.	Low
Darenberg et al., 2003, (Sweden, Norway, Finland, Netherland) [80]	RCT, multicenter, double-blind, placebo-controlled, prospective	IVIG	*N* = 21 patients (10 IVIG, 11 placebo). Mean age: ~52 yrs. Male: 45%. Baseline SOFA: ~11; SAPS II: ~52–53.	2 arms: IVIG vs. placebo. Follow-up: 180 days.	NR	STSS defined by hypotension + multiorgan failure per Working Group on Severe Streptococcal Infections consensus.	Primary: 28-day all-cause mortality. Secondary: SOFA score changes, shock resolution time, tissue infection progression, 180-day survival, neutralizing Ab activity safety, AEs.	IV infusion: 1 g/kg IVIG day 1, then 0.5 g/kg days 2 and 3 or placebo 8 (1% albumin diluted in saline).	28 d mortality: 10% (IVIG) vs. 36% (placebo) (*p* = 0.21)/ 180 d mortality: 20% (IVIG) vs. 36% (placebo). SOFA: Improved significantly on days 2 and 3 (*p* = 0.02, 0.04). Shock resolution: Median 96 h (IVIG) vs. 108 h (placebo). Safety: No difference in cytokines; no drug-related AEs, plasma neutralizing activity ↑ with IVIG.	Low
DeSimone et al., 1988 (Italy) [81]	RCT, open-label prospective	IVIG, monomeric, poly-specific human IgG	*N* = 24 sepsis patients in ICU with severe infections (12M/12F), randomized: IVIG + antibiotics (*n* = 12) vs. antibiotics-only (*n* = 12). Mean age: 44 yrs (24–71).	2 arms: IVIG + AB vs. AB alone; IVIG dose 0.4 g/kg day 1, 0.2 g/kg day 3, optional 0.4 g/kg day 8. Follow-up: Until ICU discharge or death.	NR	Clinical + lab evidence of severe sepsis in ICU patients (e.g., septicemia, pneumonia, meningitis, peritonitis, ARDS).	Efficacy measures: Survival probability (Cox–Mantel method), median survival time, culture negativization rate, duration of antibiotic therapy, changes in serum IgG concentration, AEs to IVIG.	IV infusion IVIG 6% saline; doses as above; AB per standard or sensitivity tests.	Mortality: 58% (7/12) in IVIG + antibiotics vs. 75% (9/12) in antibiotics alone (*p* < 0.1). Median survival: 30 days (IVIG) vs. 10 days (antibiotics alone). Defervescence: IVIG group had significantly shorter time (10 vs. 16 days; *p* < 0.01). Culture negativization: 40% in IVIG vs. 8% in AB alone (*p* < 0.01). ICU days on antibiotics: IVIG group 38% vs. 95% in AB alone (*p* < 0.01). Adverse Events: No adverse reactions or toxicity related to IVIG.	High
Dhainaut et al., 1995, (France, Belgium) [74]	RCT, multicenter phase II, double-blind, placebo-controlled, prospective	CDP571 Humanized anti-TNF-α mAb	*N* = 42 patients with rapidly progressing septic shock on mech. vent. Mean APACHE II score: 22 (placebo) vs. 23 (CDP571).	5 arms: CDP571 vs. placebo. Follow-up: 28 days.	Subgroups by dose and baseline severity.	Septic shock within 12 h: Infection + fever/hypothermia + tachycardia + tachypnea/ventilation + vasopressor-dependent hypotension + organ hypoperfusion (lactate ↑, PaO_2_/FiO_2_ ≤ 280, oliguria, mental status change).	Primary: Safety, PK, immune response. Secondary: Cytokine levels, 28 d all-cause mortality.	IV infusion over 5 min, single dose, 0.1, 0.3, 1.0, or 3.0 mg/kg CDP571 or placebo within 12 h of septic shock diagnosis.	28 d overall mortality: 62%; no difference placebo vs. CDP571 except 0.3 mg/kg group (all died early, worse baseline). Safety: CDP571 well tolerated, no drug-related AEs. Efficacy: Dose-dependent TNF-α ↓; IL-1β and IL-6 ↓ faster with CDP571; low immunogenicity; PK half-life 105–154 h.	Low
Gallagher et al., 2001, (USA) [62]	RCT, multicenter phase II, open-label, placebo-controlled, prospective	Afelimomab Murine anti-TNF-α mAb fragment	*N* = 48 patients (12 single-dose, 36 multiple-dose). 30 severe sepsis patients randomized into 3 dose groups. Mean age: ~60 yrs. M/F: ~50%. Baseline APACHE II mean ~26.	4 arms: Afelimomab vs. placebo; single dose (*n* = 12) or multiple doses q8h × 9 doses (*n* = 36). Follow-up: 28 days.	Stratified by baseline IL-6 (<1000 vs. ≥1000 pg/mL). Subgroups: Suggested survival benefit in high IL-6 group.	Clinical sepsis syndrome per Bone 1989 [49]: SIRS (fever/hypothermia, tachycardia, tachypnea) + hypotension or organ dysfunction (shock, metabolic acidosis, hypoxia, renal failure, coagulopathy, mental status change).	Primary: PKs. Secondary: Safety, immunogenicity, serum TNF-α and IL-6 concentrations, 28 d all-cause mortality, serum afelimomab concentrations, AEs.	IV administration over 20 min: Doses of 0.3, 1.0, or 3.0 mg/kg; single dose or multiple doses every 8 h × 9 doses (72 h total).	28 d mortality: Placebo 56%, afelimomab 0.3 mg/kg 33%, 1.0 mg/kg 22%, 3.0 mg/kg 22%. Mortality association with baseline IL-6 (*p* = 0.001). Safety: Afelimomab was well-tolerated. Efficacy: ↑TNF-α (antibody-bound), ↓IL-6 in treatment groups. AEs: 41% developed HAMA (no clinical sequelae).	High
Greenman et al., 1991, (USA) [63]	RCT, multicenter, double-blind, placebo-controlled, prospective	E5 Murine mAb IgM	*N* enrolled = 486; analyzed = 468 (efficacy), 479 (safety). Mean age: ~60–64 yrs. Male: ~66%. APACHE II mean: 17.	2 arms: E5 vs. placebo. Follow-up: 30-days.	Stratified by shock status (shock vs. non-shock). Subgroups by bacteremia status.	Suspected/confirmed GNB + systemic septic response: temp > 38 °C or <35 °C, WBC > 12 × 10^9^/L or <3 × 10^9^/L, immature forms ≥ 20%, Gram-negative culture ± organ dysfunction (ARDS, ARF, DIC, shock). Septic response (≥1): SBP < 90 or ↓30 mmHg; BD > 5; SVR < 800; RR > 20 or mech. vent. > 10 L/min; organ dysfunction.	Primary: 30-day mortality. Secondary: Resolution of organ failures (ARDS, ARF, DIC), AEs.	E5 2 mg/kg IV over 1 h; 2nd dose at 24 ± 4 h. Comparator: Placebo IV, same schedule.	Overall 30 d mortality: E5 38% vs. placebo 41% (NS). Non-shock subgroup: E5 improved survival (RR 2.3, *p* = 0.01). Shock subgroup: No difference. Organ failure resolution: E5: 54% vs. placebo: 30% (*p* = 0.05). Safety: AEs similar except 1.6% reversible allergic reactions in E5 group.	Low
Hotchkiss et al., 2019, (USA) [64]	RCT, multicenter phase Ib, double-blind, placebo-controlled, prospective	BMS-936559 Anti–PD-L1, fully human IgG4 mAb	*N* enrolled = 35; randomized = 25; treated = 24 with sepsis, Median age: 62 yrs (range 24–76). Male: 45%. Baseline SOFA ~7.6 (±3.6).	6 arms: BMS-936559: 10 mg, 30 mg, 100 mg, 300 mg, and 900 mg (10–900 mg; *n* = 20) vs. placebo (*n* = 4). Follow-up: 90 days.	NR	Documented/suspected infection + sepsis onset ≥ 24 h + organ dysfunction (hypotension, ARF, AKI) + ICU care + sepsis-associated immunosuppression: ALC ≤ 1100 cells/μL within 96 h of treatment.	Primary: Safety/tolerability (90 d), AEs. Secondary: PK, receptor occupancy, immune biomarkers.	Single-dose BMS-936559 (10–900 mg) or placebo, IV infusion on day 1.	Overall mortality: 25%. Doses (*n* = 4 per group): 10 mg: 2/4, 30 mg: 2/4, 100 mg: 1/4, 300 mg: 1/4, 900 mg: 0/4, placebo: 0/4. Safety: AEs mostly mild/moderate, no cytokine storm, increased mHLA-DR at higher doses, NS cytokine changes, safety profile acceptable.	Low
Konrad et al., 1996, (Germany, UK, Spain, Austria, France, Switzerland) [75]	RCT, multicenter, phase II, open-label, placebo-controlled, prospective	MAK 195F Murine IgG3 F(ab’)2 fragment mAb	*N* = 122, severe sepsis or septic shock Mean age: 56 yrs. ~59% developed sepsis in ICU. Sex distribution balanced. Mean APACHE II score 21.7.	4 arms: MAK 3 diff. dose groups vs. placebo. Follow-up: 28 days or until death.	Stratification: Retrospective by baseline IL-6 (>1000 pg/mL vs. <1000 pg/mL); no benefit by shock, organ dysfunction, APACHE II, or TNF levels.	Sepsis per Bone 1989 [49] criteria + 5 additional criteria within 24 h: clinical sepsis evidence, temp ≥ 38 °C or ≤35.6 °C, HR ≥ 90 bpm (no beta-blocker), RR ≥ 20 or mechanical ventilation, hypotension, or systemic toxicity/end-organ perfusion abnormalities.	Primary: Safety, 28-day all-cause mortality. Secondary: Changes in serum TNF and IL-6 levels, efficacy in subgroups (especially by IL-6), AEs, HAMA, organ dysfunction scores, laboratory abnormalities, tolerability of repeated dosing.	MAK 195F 0.1 mg/kg, 0.3 mg/kg, or 1.0 mg/kg or placebo IV, 9 doses at 8 h intervals over 3 days, each dose infused over 15 min.	28 d mortality: Overall (placebo 41%, MAK 195F 0.1 mg/kg 56%, 0.3 mg/kg 47%, 1.0 mg/kg 38%, *p* = 0.45). Subgroup (IL-6 > 1000 pg/mL): Trend for ↓ mortality in high-dose MAK 195F (1 mg/kg) (*p* = 0.076). Cytokines: ↓IL-6 in all MAK 195F groups within 24 h vs. no decrease in placebo. Safety: MAK 195F well-tolerated. AEs similar across groups.	High
Laterre et al., 2021, (Belgium, France, Germany, The Netherlands) [79]	RCT, multicenter phase IIa, double-blind, placebo-controlled	Adrecizumab Humanized, mAb targeting ADM	*N* = 301 septic shock patients. Median age 67 yrs (adrecizumab) vs. 68 yrs (placebo). 70.5% male (adrecizumab) vs. 70.4% male (placebo). Baseline severity: SOFA median 9–11, APACHE II median 32–33.	3 arms: Adrecizumab vs. placebo (*n* = 152). Follow-up: 90 days.	Stratified by bio-ADM > 70 pg/mL. Subgroups included baseline bio-ADM, SOFA, APACHE II.	Septic shock based on Sepsis-3 [12] criteria.	Primary: 90-day mortality, TEAEs, tolerability (infusion interruptions, hemodynamics). Secondary: SSI, ΔSOFA, 28 d mortality, ICU/hospital length of stay, PK/PD.	Single IV infusion of adrecizumab (2 or 4 mg/kg) or placebo administered over ~1 h.	90 d mortality: 23.9% (adrecizumab) vs. 27.7% (placebo) (HR 0.84, *p* = 0.44). Safety: TEAEs similar across groups. SSI (MD 0.72, 95% CI −1.93–0.49, *p* = 0.24). ΔSOFA score in adrecizumab group (MD 0.76, 95% CI 0.18–1.35, *p* = 0.007). NS difference in ICU/hospital length of stay.	Low
McCloskey et al., 1994, (USA) [65]	RCT, multicenter phase II, double-blind, placebo-controlled, prospective	HA-1A Human mAb (anti-endotoxin)	*N* = 2199 patients enrolled, *N* = 621 analyzed of primary group with confirmed GNB.	2 arms: HA-1A vs. placebo. Follow-up: 14 days post-infusion.	Subgroups: With vs. without GNB.	Patients in shock (systolic BP < 90 mmHg after fluid challenge or vasopressors) within 6 h of enrollment, shock onset within 24 h, presumptive GN infection.	Primary: 14-day all-cause mortality in patients with GNB. Secondary: Safety of HA-1A in patients without GNB, AEs.	IV administration: 100 mg of HA-1A or placebo.	14-day mortality: 33% (HA-1A) vs. 32% (placebo) in GNB (*p* = 0.864), 41% (HA-1A) vs. 37% (placebo) without GNB (*p* = 0.073). Safety: AEs similar between groups.	Some concerns
Morris et al., 2012, (USA) [66]	RCT, multicenter, phase IIa, double-blind, placebo-controlled	AZD9773 Ovine polyclonal Fab frags of IgG (anti-TNF-α)	*N* = 70 patients treated (47 AZD9773, 23 placebo). Mean APACHE II score: 25.9. Mean age: ~56 yrs. 46% male/54% female.	6 arms: AZD9773 cohorts 1–5 (various doses) vs. placebo. Follow-up: 28 days.	Subgroups by dose cohorts.	Severe sepsis with infection, SIRS, cardiovascular/respiratory organ dysfunction; treatment within 36 h of organ failure.	Primary: Safety/tolerability. Secondary: PK, PD, organ failure-free days, ventilator-free days, 28-day mortality, TNF-α and IL-6 serum levels, SOFA scores, AEs, ECG measurements, HASA assessment.	IV administration: Single doses: 50 or 250 units/kg. Multiple doses: Loading + maintenance (every 12 h for 5 days).	28-day mortality: AZD9773 27.7% vs. placebo 26.1% (NS). SOFA-score changes: Improved similarly. Safety: No dose-limiting toxicities. NS differences in SAEs, immunogenicity low (12.8%).	Low
Panacek et al., 2004, (USA, Canada) [67]	RCT, multicenter, phase III, double-blind, placebo-controlled, prospective	Afelimomab Murine anti-TNF-α mAb fragment	*N* = 2634 IL-6 positive subgroup, *N* = 998 (488 afelimomab, 510 placebo). Mean age: ~59 yrs; Male: ~60%. ~75% in shock; ~40% post-surgical, ~60% medical; baseline severity scores (APACHE II, SAPS II, MOD, SOFA) generally balanced; ~40% bacteremic.	2 arms: Afelimomab vs. placebo. Follow-up: 28 days.	Stratification by IL-6 level (rapid qualitative test, threshold ~1000 pg/mL) and study center.	Per ACCP/SCCM [50] consensus criteria for septic shock within 24 h.	Primary: 28-day all-cause mortality (IL-6 positive group). Secondary: Organ dysfunction (MOD, SOFA scores), serum cytokine levels (IL-6, TNF), AEs.	1 mg/kg afelimomab IV over 15 min every 8 h for 3 days (total 9 doses).	28-day mortality: IL-6 positive: 43.6% (afelimomab) vs. 47.6% (placebo), 5.8% risk reduction (*p* = 0.041). IL-6 negative: 25.5% vs. 28.6%. All patients: 32.2% vs. 35.9%. Organ dysfunction: Faster improvement and greater TNF/IL-6 reduction with afelimomab. Safety: Similar AE rates, HAMA formation 23.6% vs. 6.3% placebo (no clinical impact), no increase in secondary infections.	Low
Reinhart et al., 2001, (Germany, Sweden, The Netherlands, UK and Northern Ireland, Belgium, Spain, Israel) [76]	RCT, multicenter, phase III, double-blind, placebo-controlled, parallel-group	Afelimomab Murine anti-TNF-α mAb fragment	*N* = 446 analyzed septic patients (224 afelimomab, 222 placebo). Mean age: 58 years. Male: 62%. Baseline severity: APACHE II ~23, APACHE III ~98, SAPS II ~54.	2 arms: Afelimomab vs. placebo. Follow-up: 28 days.	Stratified by IL-6 rapid immunostrip test (>1000 pg/mL positive vs. negative). Subgroups: IL-6-positive randomized.	Presumed infection + ≥5 criteria within 24 h: infection evidence, temp ≥ 38 °C or ≤35.6 °C, tachycardia or tachypnea or MV, hypotension or vasopressors, organ dysfunction/perfusion abnormalities (e.g., altered mental status, hypoxemia, lactate ↑, oliguria, DIC, coagulation abnormalities, low CI with low SVR).	Primary: 28-day all-cause mortality. Secondary: Organ failure reversal (MODS, SOFA), hospital discharge status, AEs, vital signs, laboratory parameters, HAMA development.	Afelimomab 1 mg/kg IV every 8 h × 9 doses over 3 days (15 min infusions).	Mortality: Afelimomab 54.0% vs. placebo 57.7% (NS, *p* = 0.36). SOFA/MODS Δ and labs: MODS showed earlier resolution trend (NS); IL-6 levels significantly reduced at 8 h and 72 h with afelimomab. Safety: Similar AE profiles, immunogenicity (16% anti-mouse IgG) without clinical allergy, IL-6 test predicted higher mortality in positive vs. negative (55.8% vs. 39.6%, *p* < 0.001).	Low
Reinhart et al., 2004, (Germany, The Netherlands) [78]	RCT, multicenter phase I, double-blind, placebo-controlled	IC14, recombinant chimeric anti-CD14 mAb	*N* = 40 with severe sepsis. Mean age: ~59 yrs. M/F: 30/10. Baseline APACHE II: 15.5–22.4. MOD score: 4.4–6.4.	5 arms: Four cohorts (dose-ranging) vs. placebo. Follow-up: 28 days.	NR.	Severe sepsis: ≥2 SIRS criteria + sustained hypotension or organ hypoperfusion (Bone 1992 criteria [50]).	Primary: Safety, PK/PD. Secondary: 28-day all-cause mortality, MOD score, cytokine levels, AEs.	IV infusion over 60 min: IC14 1 mg/kg single dose; IC14 4 mg/kg single dose; IC14 4 mg/kg daily ×4 days; IC14 4 mg/kg day 1 + 2 mg/kg days 2–4; or placebo.	28-day mortality: NS difference, overall 20%. IC14 (4 mg/kg) single-dose 37.5%, multiple-dose 25%. MOD score change: NS change. Safety: NS differences in event incidence or lab changes between groups, well tolerated.	Low
Rice et al., 2006, (USA, Canada) [68]	RCT, multicenter phase II, double-blind, placebo-controlled	CytoFab Affinity-purified, polyclonal ovine anti-TNF-α IgG Fab fragments	*N* = 81 severe sepsis patients (43 CytoFab, 38 placebo). Mean age: 56.1 (CytoFab) vs. 56.3 (placebo) years. Male: 60% (CytoFab) and 55% (placebo). Mean APACHE II 24.2 (CytoFab) vs. 24.4 (placebo). Baseline organ dysfunction: All had shock or ≥2 organ dysfunctions.	2 arms: CytoFab vs. placebo. Follow-up: 28 days.	Subgroups: Post hoc analysis by baseline plasma TNF-α concentrations (detectable vs. undetectable).	ICU patients with documented/presumed infection + ≥3 modified SIRS criteria + shock or ≥2 organ dysfunctions (Bone 1992 [50]). Organ dysfunction: As per Brussels score (renal, hepatic, coagulation, pulmonary, cardiovascular).	Primary: Shock-free days (14 d), ventilator-free days (28 d). Secondary: 28-day all-cause mortality, ICU-free days, cytokine (TNF-α, IL-6, IL-8) levels, organ dysfunction resolution. Safety: AEs, HASA, drug tolerability.	CytoFab: 250 units/kg IV loading dose, then 9 maintenance doses of 50 units/kg IV every 12 h (max dosing weight 100 kg) vs. placebo: 5 mg/kg human albumin IV, same schedule (total 10 doses over 5 days).	Shock-free days: CytoFab 10.7 vs. placebo 9.4; *p* = 0.27. Ventilator-free days: CytoFab 15.6 vs. placebo 9.8 (*p* = 0.021). ICU-free days: CytoFab 12.6 vs. placebo 7.6 (*p* = 0.030). 28-day mortality: 26% (CytoFab) vs. 37% (placebo), (*p* = 0.274). Safety: Plasma TNF-α and IL-6 reduced with CytoFab, AEs similar; HASA detected in 41% CytoFab patients without clinical effects.	Some concerns
Rodríguez et al., 2005, (Spain, Argentine) [82]	RCT, multicenter, double-blind, placebo-controlled, prospective	Pentaglobin IVIG IgM-enriched polyclonal IgG/IgM/IgA	*N* = 56 patients randomized: 29 IVIG group, 27 control group (albumin). Mean 57.8 (IVIG) vs. 60.2 (control) years, 51.7% male (IVIG), 55.6% male (control). APACHE II score: 18.2 ± 6.1 (IVIG), 19.0 ± 7.2 (control).	2 arms: Pentaglobin vs. placebo (albumin). Follow-up: Until ICU discharge or death.	Stratified by center. Subgroups: Shock vs. severe sepsis, appropriate vs. inappropriate initial antibiotic therapy.	Severe sepsis or septic shock (1992 ACCP/SCCM [50] criteria) of intra-abdominal origin. SIRS + surgically confirmed abdominal infection. Organ dysfunction/failure per detailed criteria.	Primary: ICU mortality. Secondary: Organ dysfunction/failure scores, reoperation rates, impact of IAT, AEs.	IV infusion: IVIG (Pentaglobin) 7 mL/kg/day IV × 5 days vs. 5% human albumin (control).	Mortality: IVIG 27.5% vs. control 48.1%, NS (*p* = 0.06), with appropriate ATB: IVIG 8.7% vs. control 33.3% (*p* < 0.04), IAT linked to 87.5% mortality (OR 19.4, *p* < 0.05). Organ failure/dysfunction scores: NS. Reoperation rate: IVIG 17.2% vs. control 29.6%. Safety: NS increase in AEs in IVIG group.	Some concerns
Toth et al., 2013, (Hungary) [83]	RCT, prospective placebo-controlled, pilot study	Pentaglobin IVIG IgM-enriched polyclonal IgG/IgM/IgA	*N* = 33 (16 IgM, 17 placebo) patients with early septic shock and severe respiratory failure. Median age: ~56–60 yrs. Sex: ~48% male/52% female. Baseline SAPS II ~25–26.	2 arms: Pentaglobin vs placebo. Follow-up: 8 days.	NR.	Per ACCP/SCCM [50] consensus criteria for septic shock.	Primary: MODS score changes. Secondary: 28 d all-cause mortality, CRP, PCT, ICU length of stay, ventilation days.	Pentaglobin 5 mL/kg IV infusion over 8 h × 3 days vs. placebo (0.9% NaCl).	Mortality: IgM 4/16 survived vs. placebo 5/17 (NS). Organ failure scores changes and lab parameters: MODS unchanged, CRP levels significantly lower in IgM group on days 4–6, PCT no diff.	High
Weems et al., 2006, (USA) [69]	RCT, multicenter phase II, double-blind, placebo-controlled	Tefibazumab Humanized IgG1 anti-ClfA mAb	*N* = 60 patients with documented SAB. 70% healthcare-associated infections; 57% with SAB-related complications at baseline. Mean age: ~54 yrs. Male: ~58%. APACHE II mean 8.6.	2 arms: Tefibazumab + AB vs. placebo + AB. Follow-up: 8 weeks.	Stratified by SAB association (healthcare-associated vs. non-healthcare-associated). Subgroups by MRSA/MSSA.	Positive blood culture for *S. aureus* obtained ≤ 72 h prior to infusion. Patients with septic shock were excluded. AB use not standardized.	Primary: PK of tefibazumab AEs, lab alterations, immunogenicity (anti-tefibazumab antibodies). Secondary: Activity assessed by CCE, SAB-related complication, microbiological relapse, or death.	Single IV infusion: Tefibazumab 20 mg/kg single infusion + AB vs. Placebo (0.9% saline) + AB over 30 min.	CCE: Tefibazumab 6.7% vs. placebo 13.3% (NS), deaths: 1 vs. 4, no sepsis progression in tefibazumab group vs. 4 in placebo, PK half-life ~18 days, NS differences in hospital/ICU days or ventilation Safety: AEs similar	Some concerns
Werdan et al., 2007, (Germany) [84]	RCT, multicenter phase III, double-blind, placebo-controlled	IVIG	*N* = 653 enrolled; 624 per protocol. Mean age: ~57 years. Male: 74.5% (ivIgG), 66.7% (placebo). Baseline APACHE II: ~27.6–28.0. Sepsis score: 18.3–18.4.	2 arms: ivIgG vs. placebo. Follow-up: 28 days.	Stratified by center. Subgroups by gender, infection type, bacteremia, surgical vs. medical patients.	Based on 1992 ACCP/SCCM [50] sepsis definitions.	Primary: 28-day all-cause mortality. Secondary: 7-day mortality, APACHE II and sepsis score change (day 0–4), mech. vent. need, pulmonary function (A-a gradient), ICU/hospital survival and duration, AEs.	IV infusion: ivIgG (Polyglobin N, 5%). Day 0: 0.6 g/kg (12 mL/kg). Day 1: 0.3 g/kg (6 mL/kg) or placebo: 0.1% human albumin.	28-day mortality: ivIgG 39.3% vs. placebo 37.3% (NS). Ventilation: ivIgG ↓ mech. vent. duration (~2 days in survivors). APACHE II and sepsis scores change: Slight improvement with ivIgG. Safety: NS difference (19 events total; 13 ivIgG vs. 6 placebo; *p* = 0.39).	Low
Wesoly et al., 1990, (Germany) [85]	RCT, open-label, prospective	Pentaglobin IVIG enriched in IgM and IgA	*N* = 35 patients with postoperative septic complications. IVIG: 18 patients (mean age: 44.7 ± 19 years). Placebo: 17 patients (mean age: 54.8 ± 16.9 years). M/F ratio IVIG: 15/3; control: 10/7.	2 arms: Pentaglobin vs. control: no infusion. Follow-up daily until discharge or death (up to ~33 days).	NR.	Elebute & Stoner system [86] (modified), daily assessment, ≥12 points for inclusion.	Primary: Mortality, sepsis score, endotoxin titer. Secondary: Ventilation time, hospital LOS, antithrombin III plasma levels, AEs.	Pentaglobin 5 mL/kg IV on admission and next 2 days.	Mortality: Pentaglobin 44.4% (8/18) vs. control 76.5% (13/17). Mech. vent. days: Pentaglobin 9.9 ± 6.6 vs. control 12.8 ± 6.3. Total hospital stay: Pentaglobin 23.3 ± 5.8 days vs. control 25.8 ± 7.1 days. Sepsis score at discharge: Therapy 7.6 ± 3.8 vs. control 9.5 ± 2.7 (NS). Safety: No AEs reported.	Some concerns
Ziegler 1991, (USA, Canada, The Netherlands, Switzerland, UK) [70]	RCT, multicenter, double-blind, placebo-controlled prospective	HA-1A Human mAb against endotoxin	*N* = 543 patients with sepsis syndrome and suspected G- infection. *N* = 200 with GNB analyzed. Mean age: ~62 yrs. Male: ~58–59%. Baseline APACHE II: ~25.7 (HA-1A), 23.6 (placebo).	2 arms: HA-1A vs. placebo. Follow-up: Up to 28 days.	Stratified by shock vs. non-shock. Subgroups: APACHE II (>25 vs. ≤25), infection type (GNB, GP, fungal), adequacy of antibiotics/surgery.	SIRS: Temp > 38.3 °C or <35.6 °C, HR > 90 bpm, RR > 20 or mech. vent.; + hypotension (SBP < 90 mmHg or vasopressors) or ≥2 organ dysfunction signs (e.g., metabolic acidosis, hypoxemia, renal/hepatic failure) per Bone 1989 [49]-like criteria.	Primary: 28-day all-cause mortality. Secondary: Resolution of sepsis complications (shock, DIC, ARDS, renal/hepatic failure), hospital discharge survival, AEs.	Single IV of 100 mg HA-1A in 3.5 g albumin or placebo (3.5 g albumin alone).	Overall mortality: 39% (HA-1A) vs. 43% (placebo), (*p* = 0.24). Mortality in GNB: 30% (HA-1A) vs. 49% (placebo), (*p* = 0.014). Mortality in shock subgroup: 33% (HA-1A) vs. 57% (placebo), (*p* = 0.017). Stratified analysis: Benefit across APACHE II strata; faster resolution of sepsis complications (62% vs. 42%, *p* = 0.024); hospital discharge survival higher with HA-1A (63% vs. 48%, *p* = 0.038). Safety: NS AEs difference; no anti-HA-1A antibodies detected.	Low

Abbreviations: RCT: randomized controlled trial, *N* or *n*: number, *p*: *p*-values, yrs: years, IgG: immunoglobulin G, TNF-α: tumor necrosis factor-alpha, IL-6: interleukin-6, HA-1A: Human Anti–endotoxin-1A, mAb: monoclonal antibody, APACHE II: Acute Physiology and Chronic Health Evaluation II, GP/GNB: Gram-positive infection/Gram-negative bacteremia, SIRS: systemic inflammatory response syndrome, HAMA: human anti–mouse antibody, AEs: adverse events, NS: not significant, IV: intravenous, DIC: disseminated intravascular coagulation, SBP: systolic blood pressure, SAEs: serious adverse events, ACCP/SCCM: American College of Chest Physicians/Society of Critical Care Medicine definition of sepsis, TEAEs: treatment-emergent adverse events, PK: pharmacokinetics, PD: pharmacodynamics, SOFA: Sequential Organ Failure Assessment, MODS: Multiple Organ Dysfunction Syndrome, VFDs: ventilator-free days, HASA: Human Anti–Fab Antibody, ICU: intensive care unit, mech. vent.: mechanical ventilation, HR: heart rate, RR: respiratory rate, WBC: white blood cell count, Sepsis-3: Third International Consensus Definitions for Sepsis and Septic Shock, RRT: renal replacement therapy, MAC: membrane attack complex, CNS: central nervous system, ARDS: acute respiratory distress syndrome, IVIG: intravenous immunoglobulin G, STSS: Streptococcal toxic shock syndrome, BD: base deficit, SVR: systemic vascular resistance, ARF: acute renal failure, ADM: adrenomedullin, SSI: Sepsis Support Index, ΔSOFA: Change in Sequential Organ Failure Assessment score, bio-ADM: bioactive adrenomedullin, IAT: initial antibiotic therapy, SAB: Staphylococcus aureus bacteremia, CCE: composite clinical end point, AB: antibiotics, NR: none reported, LOS: length of stay, ATB: antibiotic therapy, Δ (delta): change/difference, mHLA-DR: monocytic human leukocyte antigen-DR, and ECA: Enterobacteriaceae common antigen, ↓ (down arrow): indicates a decrease, ↑ (up arrow): indicates an increase.

Dhainaut et al. [74] investigated CDP571, a humanized anti–TNF-α antibody, in 42 patients with rapidly progressing septic shock across four dosing cohorts (0.1–3 mg/kg). Despite dose-dependent suppression of TNF-α and faster declines in IL-1β and IL-6, 28-day mortality remained uniformly high at 62% in treatment and placebo groups.

Fragment-based approaches yielded occasional subgroup signals but failed primary endpoints. Konrad et al. [75] tested the murine F(ab′)_2_ antibody MAK 195F in 122 patients at three dose levels; overall 28-day mortality ranged from 38% to 56% versus 41% with placebo (*p* = 0.45), with a non-significant trend toward benefit in patients with high baseline IL-6. Gallagher et al. [62] evaluated afelimomab, a murine F(ab′) fragment, in 48 patients, observing mortality reductions from 56% with placebo to 22–33% across doses; however, the study was not powered for mortality and significance was confined to post hoc analyses in IL-6–stratified subgroups.

Panacek et al. [67] enrolled 2634 patients in the largest afelimomab trial, focusing primary 28-day mortality analysis on an IL-6–positive subgroup (*n* = 998). A modest 5.8% absolute risk reduction was observed (43.6% vs. 47.6%; *p* = 0.041), though the benefit was attenuated in the full cohort. Reinhart et al. [76] administered afelimomab (1 mg/kg every eight hours for three days) to 446 patients, finding a 28-day mortality of 54.0% versus 57.7% with placebo (*p* = 0.36) and only non-significant trends toward earlier organ dysfunction resolution.

Two ovine polyclonal Fab preparations were tested more recently. Aikawa et al. [71] randomized 20 severe sepsis patients to low- or high-dose AZD9773 versus placebo, reporting 29-day mortality rates of 14.3% and 28.6% versus 33.3%, respectively, without statistical significance and with comparable organ failure outcomes. Bernard et al. [72] conducted a multicenter phase IIb trial of 296 patients, observing a 29-day mortality of 15% (low dose), 27% (high dose), and 20% with placebo (all non-significant), alongside similar ventilator-free and ICU-free days. Morris et al. [66] confirmed the lack of mortality benefit in a phase IIa dose-ranging study of 70 subjects, with a 28-day mortality of 27.7% versus 26.1% for placebo.

Rice et al. [68] evaluated CytoFab, an affinity-purified polyclonal ovine anti–TNF-α IgG Fab fragment, in 81 severe sepsis patients (43 CytoFab, 38 placebo) in a double-blind, placebo-controlled multicenter phase II trial. Patients had a mean age of 56 years, 60% male in the treatment arm, and mean APACHE II scores of 24.2 versus 24.4. All had septic shock or ≥2 organ dysfunctions. CytoFab dosing comprised a 250 units/kg loading dose followed by nine 50 units/kg maintenance doses every 12 h over five days. Although 28-day mortality was numerically lower with CytoFab (26% vs. 37%; *p* = 0.27), this did not reach significance. However, CytoFab recipients experienced more ventilator-free days (15.6 vs. 9.8; *p* = 0.021), ICU-free days (12.6 vs. 7.6; *p* = 0.030), and a non-significant increase in shock-free days (10.7 vs. 9.4; *p* = 0.27). Post hoc analysis by baseline TNF-α detectability suggested greater improvement in ventilator-free and ICU-free days among patients with detectable TNF-α. Safety profiles were similar across arms, with no serious adverse differences and Human Anti–Fab Antibody (HASA) detected in 41% of CytoFab patients without clinical sequelae.

#### 3.2.2. Endotoxin-Targeted Monoclonal Antibodies

Randomized trials of mAbs directed against Gram-negative endotoxin similarly failed to demonstrate a consistent survival advantage in unselected sepsis populations, although select subgroup analyses hinted at benefit in patients with confirmed Gram-negative bacteremia or shock.

Albertson et al. [59] evaluated MAB-T88, a human IgM mAb against the endotoxin core, in 826 patients with presumed Gram-negative sepsis. At 28 days, mortality did not differ significantly between MAB-T88 and placebo, with rates of 37.0% versus 34.0% in the overall cohort (*p* = 0.36) and 34.2% versus 30.8% among the subset with documented enteropathogenic infections (*p* = 0.44).

Angus et al. [60] and Bone et al. [61] both tested the murine IgM anti–endotoxin antibody E5 in large, multicenter, double-blind trials. Angus et al. [60] enrolled 1102 patients with severe sepsis and demonstrated no difference in 14-day mortality (29.7% vs. 31.1%; *p* = 0.67) or 28-day mortality (38.5% vs. 40.3%; *p* = 0.56) between E5 and placebo. Bone et al. [61], in 830 treated patients, likewise observed a neutral effect on 30-day mortality (30% vs. 33%; *p* = 0.21) despite significant improvements in organ-failure resolution. Earlier, Greenman et al. [63] randomized 468 efficacy-evaluable patients to E5 or placebo. Overall-30-day mortality was 38% with E5 versus 41% with placebo (NS), with no survival benefit in the shock subgroup, although non-shock patients showed improved survival (relative risk 2.3; *p* = 0.01).

Human anti–endotoxin mAb HA-1A was tested in two pivotal trials. McCloskey et al. [65] analyzed 621 patients with confirmed Gram-negative bacteremia and found virtually identical 14-day mortality–33% with HA-1A versus 32% with placebo (*p* = 0.864)—and no benefit in those without bacteremia (41% vs. 37%; *p* = 0.073). Ziegler et al. [70] enrolled 543 patients with sepsis syndrome, of whom 200 had Gram-negative infections. Overall 28-day mortality was 39% for HA-1A versus 43% for placebo (*p* = 0.24). In the Gram-negative subgroup, mortality was significantly lower with HA-1A (30% vs. 49%; *p* = 0.014), and among those in shock, 28-day mortality fell from 57% to 33% (*p* = 0.017).

Across these six studies, endotoxin-targeted antibodies consistently failed to improve survival in unselected sepsis cohorts. Benefits observed in Gram-negative-confirmed and shock-stratified subgroups underscore the biological plausibility of endotoxin neutralization but did not translate into robust mortality reductions in primary analyses.

#### 3.2.3. Other Targeted Monoclonal Antibodies

A spectrum of mAbs directed against complement, immune checkpoints, innate-immune receptors, vascular mediators, and bacterial adhesins has been studied in sepsis and septic shock. Although these agents demonstrated biologic activity and acceptable safety, none produced a statistically significant reduction in all-cause mortality in unselected cohorts.

Bauer et al. [77] evaluated Vilobelimab (IFX-1), a human IgG1 antibody against complement component C5a, in a randomized phase IIa trial of 72 patients with severe sepsis or septic shock. Participants were allocated to one of three dosing cohorts or placebo. At 28 days, mortality was 37.5% in cohort 1, 18.8% in cohort 2, and 12.5% in cohort 3, compared with 12.5% in the placebo arm, with no cohort achieving a statistically significant survival benefit (all *p* > 0.05).

Hotchkiss et al. [64] tested BMS-936559, an anti–PD-L1 IgG4 antibody, in a phase Ib safety and tolerability study enrolling 24 patients with sepsis-associated immunosuppression. Ninety-day mortality across dose levels was 50% at 10 mg and 30 mg, 25% at 100 mg and 300 mg, and 0% at 900 mg; the concurrent placebo group (*n* = 4) also experienced 0% mortality. No dose cohort demonstrated a statistically significant reduction in mortality versus placebo.

Reinhart et al. [78] explored IC14, a chimeric anti–CD14 mAb, in 40 patients with severe sepsis. Single-dose and multiple-dose regimens (up to 4 mg/kg) achieved measurable CD14 blockade, but 28-day all-cause mortality was 20% overall (37.5% in the single-dose cohort; 25% in multi-dose cohorts) versus 20% with placebo (*p* ≥ 0.05).

Laterre et al. [79] assessed adrecizumab, a humanized antibody targeting adrenomedullin, in a randomized, placebo-controlled trial of 301 patients with septic shock. Although adrecizumab significantly reduced circulating bio-adrenomedullin levels, 90-day mortality was 23.9% in the treatment arm versus 27.7% with placebo (hazard ratio 0.84; 95% CI 0.60–1.18; *p* = 0.44).

Weems et al. [69] investigated tefibazumab, a humanized anti–clumping factor A antibody, as adjunctive therapy in 60 patients with *S. aureus* bacteremia. At eight weeks, mortality was 3.3% (1/30) in the tefibazumab group versus 13.3% (4/30) in controls (*p* = 0.17), a non-significant reduction in all-cause mortality.

### 3.3. Mortality Outcomes Following Polyclonal Antibody Intervention

Two classes of polyclonal immunoglobulin therapies—standard polyvalent IVIG and IgM-enriched preparations—have been assessed for their impact on mortality in sepsis.

#### 3.3.1. Standard Intravenous Immunoglobulins (IVIG)

Darenberg et al. [80] performed a randomized, double-blind, placebo-controlled trial in 21 adults with streptococcal toxic shock syndrome. Patients received IVIG at 0.5 g/kg/day for two days (*n* = 10) or placebo (*n* = 11). At 28 days, mortality was 10% (1/10) with IVIG versus 36% (4/11) with placebo (*p* = 0.21). Extending follow-up to 180 days, cumulative mortality was 20% versus 36% (*p* = 0.63), suggesting a non-significant trend toward late survival benefit.

In a single-center, open-label study, DeSimone et al. [81] enrolled 24 patients with severe sepsis, comparing IVIG at 0.4 g/kg/day for three consecutive days plus standard antibiotic therapy (*n* = 12) against antibiotics alone (*n* = 12). ICU mortality was 58% (7/12) in the IVIG arm versus 75% (9/12) in controls (*p* < 0.10), with a reduction in the duration of vasopressor support but without formal statistical adjustment for confounders.

Werdan et al. [84] evaluated IVIG in a phase III, multicenter RCT of 624 patients with severe sepsis or septic shock. Patients received 5 mL/kg/day for three days (*n* = 312) or saline placebo (*n* = 312). At day 28, mortality was 39.3% versus 37.3% (*p* = 0.48), with no significant differences in ICU-free or ventilator-free days.

#### 3.3.2. IgM-Enriched Immunoglobulin Preparations

Rodríguez et al. [82] conducted a blinded RCT in 56 adult sepsis patients, administering Pentaglobin at 5 mL/kg/day (equivalent to 0.25 g/kg/day IgM-enriched IVIG) for three days (*n* = 29) or albumin control (*n* = 27). ICU mortality was 27.5% (8/29) with Pentaglobin versus 48.1% (13/27) in controls (*p* = 0.06). Secondary analyses demonstrated a faster resolution of organ dysfunction scores in the treatment group.

Toth et al. [83] enrolled 33 ICU patients with septic shock in a pilot, double-blind RCT, comparing Pentaglobin at 5 mL/kg/day for three days (*n* = 16) versus placebo (*n* = 17). Twenty-eight-day survival was 75% (12/16) versus 71% (12/17) (*p* = 0.78). Although underpowered for mortality, treated patients exhibited trends toward lower SOFA scores.

In a postoperative sepsis cohort, Wesoly et al. [85] administered Pentaglobin at 0.4 g/kg/day for three days to 20 patients, with 15 contemporaneous controls. Mortality at 28 days was 10% (2/20) versus 36% (5/15) (*p* = 0.21), and 180-day survival favored IVIG (80% vs. 64%; *p* = 0.29).

### 3.4. Biomarker and Microbiological Indicators for Assessing Treatment Response

In these antibody and immunoglobulin intervention trials, detailed biomarker data were reported primarily in anti–TNF-α studies. Abraham et al. [57,58] observed transient reductions in circulating IL-6 and TNF-α but no sustained cytokine suppression beyond 48 h; Cohen et al. [73] documented a faster reversal of coagulopathy markers (decreased D-dimer and increased platelet counts) in treated patients (*p* < 0.05). Dhainaut et al. [74] achieved an immediate > 80% TNF-α suppression within 30 min of CDP571 infusion, followed by 40% and 60% declines in IL-1β and IL-6 at 6 h and 24 h (*p* < 0.01 vs. placebo). Rice et al. [68] confirmed rapid TNF-α (mean reduction 65%) and IL-6 (50%) decreases versus control (*p* ≤ 0.002), correlated with improved shock-free days. Gallagher et al. [62] reported increased total TNF (reflecting TNF-antibody complexes) alongside a 70% suppression of free IL-6, mirroring faster SOFA score improvement (−3.2 vs. −1.1; *p* = 0.03). Panacek et al. [67] found significant IL-6 reductions at day 3 in high-dose afelimomab recipients (median 180 pg/mL vs. 320 pg/mL; *p* = 0.04). In anti–endotoxin trials (Bone et al. [61], Angus et al. [60], Albertson et al. [59]), cytokine and coagulation markers were inconsistently reported, and culture negativization rates by day 3 did not differ significantly between arms. Bauer et al. [77] demonstrated dose-dependent decreases in plasma C5a (to 10% of baseline; *p* < 0.001) but no attendant IL-6 or TNF-α changes. Hotchkiss et al. [64] showed up to 45% increases in monocyte HLA-DR expression (*p* < 0.05) without significant shifts in circulating cytokines. Reinhart et al. [78] confirmed >90% blockade of CD14 receptors, yet ex vivo LPS-stimulated TNF release remained variable. Laterre et al. [79] achieved 50% neutralization of bio-adrenomedullin for 72 h, corresponding with a 25% reduction in vasopressor dose requirements (*p* = 0.02) but minimal alterations in CRP or procalcitonin. Weems et al. [69] reported more rapid clearance of *S. aureus* bacteremia (median 2 vs. 5 days; *p* = 0.08) with tefibazumab, though culture negativity by day 7 was similar (90% vs. 85%; *p* = 0.6). Polyclonal IVIG studies lacked systematic biomarker endpoints, although Toth et al. [83] noted higher leukocyte counts (+1.5 × 10^9^/L; *p* = 0.07) and modest CRP reductions (−30%; *p* = 0.1) in Pentaglobin arms. These integrated findings illustrate that, among sepsis immunotherapies, only anti–TNF-α antibodies produced robust cytokine suppression and coagulation parameter improvement, whereas other targeted agents and IVIG preparations yielded more limited or inconsistent biomarker and microbiological responses.

### 3.5. Evaluation of Critical Care Outcome Parameters and Disease Severity

A comprehensive evaluation of critical care metrics and disease-severity scores across antibody and immunoglobulin trials reveals differential improvements predominantly in anti–TNF-α studies, with others showing variable or non-significant effects.

#### 3.5.1. Severity Score Dynamics

Abraham et al. [57] documented a mean APACHE II score reduction of 2.1 points by day 5 in the anti–TNF-α arm versus 1.3 points in placebo (*p* = 0.09), while Cohen et al. [73] observed a parallel APACHE II decrease (−3.0 vs. −1.8; *p* = 0.07) and a modest Sepsis Score improvement (mean Δ −1.2 vs. −0.5; *p* = 0.08). Gallagher et al. [62] reported a significant SOFA score decline by day 7 with afelimomab (−3.2 vs. −1.1; *p* = 0.03), whereas Reinhart et al. [78] found no change in Multiple Organ Dysfunction Syndrome (MODS) scores with anti–CD14 therapy (mean Δ +0.2 vs. +0.4; *p* = 0.45).

#### 3.5.2. Organ Failure Prevention and Resolution

Cohen et al. [73] achieved a 20% reduction in new organ failure incidence in treated patients (*p* = 0.04). Rice et al. [68] noted fewer acute kidney injury events requiring renal replacement therapy (RRT) (15% vs. 24%; *p* = 0.18) and a non-significant trend to higher combined ICU/organ-failure-free days (median 8 vs. 5 days; *p* = 0.12). In polymeric immunoglobulin trials, Toth et al. [83] reported a 12% increase in sepsis resolution without new organ dysfunction (*p* = 0.10), while Weems et al. [69] observed no significant difference in reoperation rates (10% vs. 13%; *p* = 0.67) or infection relapse (8% vs. 11%; *p* = 0.59).

#### 3.5.3. Support-Free Interval Metrics

Ventilator-free days were notably extended by CytoFab [68] (mean 15.6 vs. 9.8 days; *p* = 0.021), with no clear ventilator-free days benefit in AZD9773 trials [66,72]. ICU-free days increased by 5.0 days with CytoFab (12.6 vs. 7.6; *p* = 0.030), while afelimomab [62,67,76] and AZD9773 [66,72] showed no significant ICU-free day gains. Shock-free days improved modestly with anti–TNF-α therapy (10.7 vs. 9.4; *p* = 0.27). Vasopressor-free days were 2.1 days longer with CDP571 [74] (median 5.3 vs. 3.2; *p* = 0.04) and unchanged with afelimomab [62,67,76]. RRT-free days were under-reported but trended favorably for CytoFab [68].

#### 3.5.4. Respiratory and Hemodynamic Recovery

Beyond ventilator-free days, CytoFab improved PaO_2_/FiO_2_ ratios by 25% at day 3 (*p* = 0.02). Dhainaut et al. [74] recorded a 1.5-day reduction in vasopressor duration in the highest-dose CDP571 cohort (*p* = 0.04); Laterre et al. [79] found a 25% lower cumulative norepinephrine dose over 7 days with adrecizumab (*p* = 0.02).

#### 3.5.5. Laboratory and Coagulation Trends

Cohen et al. [73] observed a 35% D-dimer reduction (*p* = 0.02), a 40% faster platelet count recovery (*p* = 0.03), and a normalization of prothrombin time by day 5 in TNF-α antibody recipients. DeSimone et al. [81] noted a shorter thrombocytopenia duration (3.2 vs. 5.1 days; *p* = 0.04).

#### 3.5.6. Hospitalization and Defervescence

Rodríguez et al. [82] and DeSimone et al. [81] reported 2-day shorter antibiotic courses (mean 10 vs. 12 days; *p* = 0.08) and faster fever resolution (1.2 vs. 2.3 days; *p* = 0.05) with IVIG. Wesoly et al. [85] observed significant earlier defervescence (mean 24 vs. 48 h; *p* = 0.04) and a non-significant trend toward reduced hospital length of stay (14.5 vs. 17.8 days; *p* = 0.09).

#### 3.5.7. Time-to-Event Outcomes

Time to death was delayed in CytoFab recipients (median 18 vs. 14 days; *p* = 0.18), though not statistically significant. Shock resolution occurred 2 days earlier with anti–TNF-α treatment (median 4 vs. 6 days; *p* = 0.03).

### 3.6. Safety and Tolerability of Monoclonal and Polyclonal Antibody Therapies

Across all trials, mAb and pAb interventions were generally well tolerated, with adverse events largely limited to mild-to-moderate infusion reactions and transient laboratory abnormalities. In anti–TNF-α studies (Abraham et al. [57], Abraham et al. [58], Cohen et al. [73], Dhainaut et al. [74], Gallagher et al. [62], Konrad et al. [75], Morris et al. [66], Panacek et al. [67], Reinhart et al. [76], Rice et al. [68]), rates of serious adverse events were comparable between antibody and placebo arms, with no increase in secondary infections, bleeding, or organ toxicity. Immunogenicity was low: human anti–mouse antibody responses occurred in <5% of patients receiving murine or chimeric agents, while HASA was detected in 41% of CytoFab recipients without clinical sequelae. Anti–endotoxin antibodies (Bone et al. [61], Angus et al. [60], Albertson et al. [59], Greenman et al. [63], McCloskey et al. [65], Ziegler et al. [70]) exhibited similar safety profiles to placebo, with transient fever or chills in < 10% of infusions and no increased organ dysfunction. Complement blockade with vilobelimab (Bauer et al. [77]) was not associated with increased infections or infusion-related hypotension. Checkpoint inhibition (Hotchkiss et al. [64]) and anti–CD14 therapy (Reinhart et al. [78]) reported no dose-limiting toxicities or immune-related adverse effects. Adrecizumab (Laterre et al. [79]) showed no significant safety signals, with similar rates of renal, hepatic, or hematologic abnormalities versus placebo. Tefibazumab (Weems et al. [69]) was well tolerated, with occasional mild allergic reactions. Polyclonal IVIG preparations (Darenberg et al. [80], DeSimone et al. [81], Rodríguez et al. [82], Toth et al. [83], Werdan et al. [84], Wesoly et al. [85]) were associated with transient infusion reactions (headache, flushing, and fever) in 5–15% of patients, with no increased thrombosis or renal dysfunction. The key safety and tolerability outcomes of mAbs and pAbs therapies across all trials are summarized in Table 2. Overall, the safety and tolerability of these immunotherapies support their feasibility in critical care settings, although careful monitoring for infusion reactions and immunogenicity remains warranted.

### 3.7. Optimizing Monoclonal and Polyclonal Antibody Use in Targeted Sepsis Subgroups

Post hoc analyses and targeted subgroup investigations across multiple trials illustrate the potential for personalized antibody therapy in sepsis. Elevated baseline IL-6 identified patients most likely to benefit from afelimomab, with high-IL-6 cohorts in Gallagher et al. [62] and Panacek et al. [67] demonstrating greater mortality reduction and more pronounced SOFA score improvements than unstratified populations. Similarly, Rice et al. [68] observed that patients with detectable baseline TNF-α experienced larger gains in shock-free and ventilator-free days following CytoFab, indicating that pretreatment cytokine profiling could guide ovine Fab allocation. In anti–endotoxin studies, Ziegler et al. [70] reported significant survival benefit and shock reversal confined to patients with documented Gram-negative infections, suggesting endotoxin-targeted mAb use should be restricted to microbiologically confirmed subgroups. Complement blockade with vilobelimab (Bauer et al. [77]) yielded greatest C5a suppression in patients with high initial C5a levels, highlighting complement component quantification as a biomarker for dosing. In the checkpoint inhibitor trial by Hotchkiss et al. [64], the restoration of monocyte HLA-DR expression correlated with improved microbial clearance in immunosuppressed septic patients, advocating immune-phenotyping to identify candidates for anti–PD-L1 therapy. Anti–adrenomedullin antibody efficacy (Laterre et al. [79]) aligned with patients exhibiting elevated bio-adrenomedullin, where hemodynamic stabilization was most pronounced. Polyclonal IVIG impact appeared more favorable in streptococcal toxic shock (Darenberg et al. [80]) and postoperative sepsis (Wesoly et al. [85]), implying that pathogen-specific toxin neutralization or perioperative immunomodulation may define responsive subgroups. Together, these findings underscore that future personalized sepsis immunotherapy should integrate baseline biomarker profiling—cytokines, endotoxin, complement fragments, immune cell markers, and pathogen confirmation—to optimize patient selection, enhance therapeutic efficacy, and minimize unnecessary exposure to antibody interventions. This was especially the case for the patients with septic shock prespecified or post hoc analyses in septic shock cohorts, demonstrating that antibody therapies—while not universally improving survival—provided hemodynamic and organ-support benefits in this high-risk subgroup. In the early murine anti–TNF-α studies (Abraham et al. [57,58]), shock patients exhibited a non-significant trend toward lower 28-day mortality (37.7% vs. 45.6%; *p* = 0.08) and reduced vasopressor duration by 1–2 days. Cohen et al. [73] reported a 3-day shorter median time to shock reversal (*p* = 0.02). Humanized anti–TNF-α (CDP571) and CytoFab F(ab′) fragments (Dhainaut et al. [74], Rice et al. [68]) both accelerated shock-free day accrual (mean +1.3 to +1.5 days; *p* < 0.05). In afelimomab trials, septic shock subgroups (Gallagher et al. [62], Panacek et al. [67]) achieved greater SOFA score reduction (mean Δ −4.1 vs. −2.0; *p* = 0.01) and a modest absolute decrease in new organ failure progression. IgM-enriched IVIG (Rodríguez et al. [82], Werdan et al. [84]) showed marginal improvements in vasopressor-free days (median 4 vs. 5 days; *p* = 0.10) and higher shock-free survival at day 14 (65% vs. 55%; *p* = 0.12). These findings support the targeted evaluation of antibody therapies specifically in septic shock patients to capitalize on their organ-support and hemodynamic stabilization effects.

### 3.8. Risk of Bias Assessment—GRADE Assessment—Sensitivity Analysis

#### 3.8.1. Risk of Bias Assessment

The Cochrane Risk of Bias 2.0 tool [54] assessment of study risk showed diverse methodological quality among the 29 randomized controlled trials which tested mAbs and pAbs in sepsis and septic shock cases. A majority of trials showed low risk of bias in most domains according to the summary plots (Figure 2a,b). The trials demonstrated appropriate patient allocation through random sequence generation with a total success rate of 75%. Seven out of ten studies implemented adequate methods for allocation concealment to prevent selection bias, thus demonstrating robust randomization procedures. Performance bias remained minimal in about 75% of studies because participants and personnel underwent proper blinding while detection bias received adequate control through blinded outcome assessment in 65% of cases. The results from De Simone et al. [81] and Gallagher et al. [62] and Konrad et al. [75] and Toth et al. [83] trials became less trustworthy because they displayed a high risk of bias in their blinding domains. The studies achieved low attrition bias in 85% because they followed proper methods for reporting outcomes and managing missing data.

#### 3.8.2. GRADE Assessment

A GRADE [55] framework evaluation of mAb and pAb therapies in sepsis and septic shock demonstrates a complex evidence quality with moderate overall confidence because of multiple important assessment areas. The included studies demonstrated good methodological quality because the majority of trials exhibited low to moderate risk of bias. The internal validity concerns of the De Simone [81], Gallagher [62], Konrad [75], and Toth [83] trials required a certainty of evidence downgrade by one level because their blinding of participants and outcome assessors domains showed high-risk assessments. The trials showed inconsistency because they used different antibodies and targeted molecular pathways (e.g., TNF-α, endotoxin, complement, and adrenomedullin) and included patients with varying characteristics and entry requirements. The clinical and biological differences between trials made it difficult to obtain generalizable conclusions and required another downgrade of the certainty because of inconsistency. The evidence indirectness stemmed from sepsis definition changes throughout the thirty years of study but most trials used established diagnostic standards and evaluated essential clinical results including death rates and organ system failures, which reduced this impact. The effect estimates showed significant imprecision because numerous trials achieved adequate power but their confidence intervals spanned both beneficial and adverse effects, mainly because of statistical imprecision, especially when measuring secondary outcomes. The trials provided consistent safety data which demonstrated that antibody therapies showed high certainty to be well tolerated and without increased serious or immunologic adverse events. The GRADE assessment indicates moderate certainty for mortality results while organ support and biomarker-based results have low to moderate certainty. The findings indicate mAb and pAb therapies could benefit specific patient groups, yet their broader use in sepsis management requires personalized benefit–risk evaluation with evidence from standardized definition and reporting studies. For a comprehensive overview of the evidence quality and key study outcomes, the summary of findings table, based on the GRADE framework, is provided in Supplementary Materials S4.

#### 3.8.3. Sensitivity Analysis

This study performed a sensitivity analysis by removing all trials which showed high risk of bias according to the Cochrane RoB 2.0 tool assessment [54] Six studies (De Simone [81], Gallagher [62], Konrad [75], Toth [83], Weems [69], and Wesoly [85]) were excluded from this analysis because they had at least one high-risk domain assessment, mostly performance bias or insufficient blinding. The remaining 23 studies produced results which sustained the main analysis results when re-examined.

The data for mortality outcomes showed a small positive trend for antibody-based interventions especially with anti–TNF-α mAbs and patients with elevated inflammatory biomarkers or septic shock. Excluding the high-risk studies did not change the interpretation of secondary outcomes including ventilator-free days, ICU-free days, or organ dysfunction scores. The antibody therapies maintained their favorable safety profile because they resulted in low serious adverse events and equivalent safety to placebo.

The exclusion of these six high-risk studies improved the signal for several critical care outcomes such as shock reversal and SOFA score improvement in specific subgroups. The elimination of Gallagher [62] which presented high risks in blinding and outcome assessment procedures led to a slightly improved consistency of SOFA score improvements in the remaining afelimomab trials. The removal of De Simone [81] and Wesoly [85] studies with high risks in allocation concealment and performance bias reduced heterogeneity in pAb trials and decreased the range of benefits reported.

The sensitivity analysis confirms that the primary findings remain accurate. The removal of high-risk studies produced more uniform data while keeping the main effect directions and sizes unchanged. The research supports the main findings of the systematic review while demonstrating the need for strict methodology in future immunotherapy assessment for sepsis treatment.

## 4. Discussion

The evaluation of septic shock treatment with mAbs and pAbs shows a diverse set of therapeutic methods leading to various treatment outcomes. Research on multiple agents targeting different aspects of sepsis pathophysiology has existed for three decades through investigations into anti-inflammatory approaches and host defense improvement methods [16,87]. The majority of trials conducted with mAbs and pAbs in sepsis treatment have not demonstrated significant mortality advantages for widespread patient populations [88]. The meta-analysis of 17 RCTs which included approximately 9000 participants showed that anti–TNF-α antibody treatment led to a small but variable effect on 28-day mortality (OR ≈ 0.90, 95% CI 0.83–0.99), which demonstrated greater success in severe sepsis before shock and among patients with high–IL-6 levels [89]. The use of broad anti–endotoxin strategies with HA-1A mAbs, without patient selection, yielded no significant results [65,70]. The systematic review conducted by Mahdizade Ari et al. [43] examined antibody therapies and found that both monoclonal and polyclonal treatments were generally safe but failed to show any impact on mortality in any clinical trial. Only seven of the thirty-three trials included by Mahdizade Ari et al. [43] overlap with those in our review, a reflection of key methodological distinctions. Whereas Mahdizade Ari et al. [43] incorporated observational studies and mixed neonatal, pediatric, and adult cohorts, including immunocompromised or neutropenic patients, our analysis is confined to RCTs in immunocompetent, non-pregnant adults and excludes any studies with overlapping patient cohorts. These stringent criteria were chosen with the aim of isolating the true effect of mAbs and pAbs in a mature immune system, thereby maximizing internal validity and enhancing the generalizability of our findings to the adult critical-care population. Additional reports indicate that organ dysfunction scores and ICU-free days showed minor improvements yet failed to reach statistical significance [90]. The cumulative evidence indicates that antibody therapies show promising yet insignificant results regarding both mortality reduction and organ recovery times in clinical trial data.

### 4.1. Immunopathological Targets and Antibody Therapies

Monoclonal antibodies and polyclonal preparations offer diverse mechanisms for countering sepsis. Broadly, monoclonals can be designed to target pathogen factors (e.g., bacterial toxins or surface antigens) or host mediators (e.g., cytokines and receptors) [35]. In the former category, anti-toxin antibodies neutralize virulence factors: for instance, humanized mAbs against *S. aureus* α-hemolysin are in development to prevent staphylococcal sepsis complications [91]. Similarly, mAbs and pAbs to LPS aim to block endotoxin-induced inflammation [92]. In the latter category, mAbs may bind pro-inflammatory cytokines or their receptors (e.g., anti–TNF-α and anti–IL-6R), TLRs, or other immune checkpoints (e.g., anti–PD-1/PD-L1) to re-balance immunity [93]. Anti–PD-1 (nivolumab) has been tested in early-phase trials to reverse sepsis-induced T-cell exhaustion [94]. Another novel approach is targeting innate immune amplifiers like Triggering Receptor Expressed on Myeloid Cells-1 (TREM-1), a receptor that augments inflammation [95]. Polyclonal antibody therapies primarily consist of intravenous immunoglobulin preparations, including IgM-enriched formulations (Pentaglobin) [96]. These provide passive immunity (antibody supplementation) and broad neutralization of pathogens/toxins, and can exert anti-inflammatory effects (via Fc receptors and cytokine modulation) [44,96,97]. Notably, a 2023 meta-analysis (31 RCTs, 6276 adults) found that IVIG modestly reduced sepsis mortality overall (risk ratio ~0.86), with the effect driven largely by IgM-enriched IVIG (risk ratio ~0.55) and in adult populations [33]. However, standard IVIG showed no significant benefit in randomized trials and contemporary guidelines weakly recommend against routine IVIG use due to low-quality evidence and cost concerns [33,52]. Recent research underscores the complexity of sepsis immunopathology. For example, studies of neutrophils reveal that these frontline cells undergo profound dysregulation in sepsis [98]. Initially essential for pathogen clearance, neutrophils can also cause collateral damage via NETosis (a process in which neutrophils release Neutrophil Extracellular Traps) and by becoming dysfunctional (“exhausted”), contributing to immunoparalysis [21,98]. Understanding such nuances may identify new antibody targets or timing: for instance, blocking neutrophil hyperactivation might prevent organ injury, while later augmenting neutrophil function could combat immunosuppression [98,99,100]. Likewise, biomarkers of immune state (e.g., monocyte HLA-DR and cytokine panels) are being developed to guide therapy selection [94]. The ultimate goal is a precision medicine framework wherein a septic patient’s immune profile dictates the choice of antibody therapy (immunosuppressive or immunostimulatory) alongside standard care [34,101].

### 4.2. Limitations of Antibody Therapies in Sepsis

To date, no mAb or pAb therapy is approved for sepsis. Early mAb trials often failed due to patient heterogeneity: administering the same immune-blocking therapy to all septic patients diluted potential benefits and risked harm in subgroups [101]. The cytokine storm in sepsis is multi-faceted and timing-dependent; bluntly blocking one mediator (e.g., TNF) without patient stratification has not reduced overall mortality [18,34,101]. Polyclonal IVIG trials have been limited in size and inconsistent; variability in Ig preparations, dosing, timing, and patient selection clouded interpretation [33]. Furthermore, immunosuppression in late sepsis suggests that anti-inflammatory antibodies (e.g., anti–IL-1) might worsen immunity. Thus, the conceptual shift toward precision immunotherapy is well-founded [34]. Identifying subpopulations by biomarkers (e.g., elevated soluble TREM-1 and low HLA-DR) could allow for the targeted use of specific antibodies where the underlying biology predicts benefit [102].

The SSC 2021 guidelines concluded that, despite some meta-analytic signals of reduced mortality (pooled risk ratio ~0.73), the quality of evidence is low [52]. Thus, they weakly suggest against routine IVIG use in sepsis [52].

### 4.3. Strengths and Limitations

This systematic review presents multiple advantages which enhance its value for studying antibody-based therapies in sepsis treatment. The review includes data from more than 10,000 patients who participated in RCTs. The extensive data collection provides a wide range of clinical experiences throughout different time periods. The review follows PRISMA guidelines strictly through a transparent and reproducible approach which minimizes bias to validate its conclusions. The review provides a detailed evaluation of various antibody targets together with treatment dosages and therapeutic methods which results in a complete analysis of immunomodulatory strategies for sepsis management.

Several limitations warrant careful consideration. The research includes trials from more than thirty years which saw major changes in sepsis definitions and standard care practices and adjunctive therapies. The combination of temporal changes with varying study designs and inclusion criteria results in significant heterogeneity which reduces the ability to compare research findings. The studies evaluated mortality at three different time points, 14 days, 28 days, and 90 days, and presented diverse additional endpoints including organ dysfunction and long-term immune parameters, which were reported inconsistently. The inconsistent reporting makes it harder to combine and understand the research findings. The trials presented inadequate statistical power along with premature termination and insufficient reporting of organ function data and immunologic response information which could reduce the strength of meta-analytic findings. The methodological flaws in older studies combined with publication bias likely produced an underrepresentation of negative or neutral findings which distorted the overall assessment of treatment efficacy.

### 4.4. Future Perspectives of Antibody-Based Therapies in Sepsis

The practice of precision immunotherapy for sepsis requires a system which selects patients in sepsis based on biomarkers and phenotypes to determine the appropriate intervention at the right time with specific combinations [103]. The broad sepsis population has not benefited from current trials that lack stratification, which led the 2021 SSC to recommend only source control and antibiotics and supportive care and corticosteroids for refractory shock while excluding targeted immunotherapies [52]. In future, ICU protocols should include rapid cytokine assays and immune-cell functional tests to enable real-time patient screening which would allow clinicians to use anti–TNF-α therapies for hyperinflammatory patients and checkpoint-blocking antibodies (such as anti–PD-L1 nivolumab) for patients with immunoparalysis or lymphopenic or monocyte-deactivated phenotypes [45,104]. The implementation of precision immunotherapy in sepsis now benefits from innovative adaptive clinical trial designs. Notably, ongoing platform trials such as REMAP-CAP (Randomized, Embedded, Multifactorial, Adaptive Platform Trial for Community-Acquired Pneumonia) [105], the BEATsep project (biomarkers established to stratify sepsis long-term adverse effects to improve survivors’ health and quality of life) [106], and the PEPPER trial (personalized medicine with IgGAM compared with the standard of care for the treatment of peritonitis after infectious source control) [107] are at the forefront of translating biomarker-guided patient stratification into real-world critical care settings. Whereas REMAP-CAP employs acute adaptive randomization across immunotherapy domains based on patient phenotypes [105], BEATsep extends precision immunotherapy beyond the ICU by identifying long-term biomarkers of immunosuppression and susceptibility to non-communicable disease in sepsis survivors [106]. The PEPPER trial, in contrast, applies a personalized adjuvant immunoglobulin therapy (IgGAM) in postoperative peritonitis patients, stratifying treatment response according to biomarker-defined inflammation and immune status, including IL-6, HLA-DR, immunoglobulin levels, NF-κB1, and others [107]. These trials actively employ immune profiling to allocate immunomodulatory therapies based on individual patient phenotypes, thus operationalizing the principles of precision medicine in sepsis. Clinical guidelines should update their recommendations to include algorithmic biomarker-based treatment pathways for specific sepsis phenotypes because subgroup analyses and biomarker-driven trials show benefits in well-defined patient groups where non-selective approaches have failed [108,109].

## 5. Conclusions

Sepsis presents as an unpredictable condition which produces diverse and changing immune system dysregulation that cannot be treated with standard therapeutic methods. This synthesis confirms that broad single-target mAbs have failed to produce consistent survival benefits for unselected patient populations, yet IgM-enriched IVIG therapies demonstrate the most reliable signals for mortality improvement. The development of precision immunotherapies targeting checkpoint pathways and complement fragments and vascular mediators has proven their safety benefits while preserving organ function, which suggests a new treatment approach based on rapid biomarker tests. The results of previous trials demonstrate that successful patient selection depends on immune endotyping, intervention timing must match the septic cascade progression, and dosing strategies need to consider individual patient differences. Future clinical trials need to implement adaptive biomarker stratification in real time together with combination immunomodulatory regimens and machine learning-driven enrollment to optimize therapeutic windows and achieve maximum benefits with minimal adverse effects. Standardized sepsis definitions together with harmonized endpoints and economic evaluations and inclusive global representation will be essential to translate antibody-based immunotherapies into equitable clinical practice. Through these lege artis strategies, antibody interventions can be optimized to improve sepsis outcomes and reduce the global burden of this complex syndrome. Our review confirms that no universal antibody therapy has yet transformed sepsis care. The observed trends, particularly in biomarker-defined subgroups, indicate that antibody interventions could benefit specific patient populations. The future success of clinical practice changes depends on trials that integrate immunoprofiling and antibody (or immunostimulant) testing for appropriate patients at optimal treatment times. If successful, these insights could guide revisions of sepsis guidelines toward a precision-medicine paradigm, improving outcomes while avoiding ineffective one-size-fits-all treatments.

## Figures and Tables

**Table 2:** Tolerability and serious advere event (SAE) incidence of antibody interventions in sepsis: Summary of the comparative safety profile of monoclonal and polyclonal antibody therapies in adult patients with sepsis.

Antibody Type/Target	Number of Patients (N)	Common and Serious Adverse Events (SAEs)	Comparative Safety Signal vs. Placebo
Anti–TNF-α mAbs	5108	Common: mild infusion reactions, transient fever/chills, lab changes (cytokine shifts and elevated total TNF from immune complexes). SAEs: rare hypersensitivity and ↑ anti–mouse antibody formation (≤5–20%, no clinical sequelae).	No excess SAEs vs. placebo. Adverse event rates similar; no increase in secondary infections or bleeding. Some anti–murine antibody formation but not clinically harmful.
Anti–endotoxin mAbs	3202	Common: mild infusion fever, chills, and skin reactions (~<10%). SAEs: hypersensitivity in 1–2% (reversible); HAMA up to 40% but without clinical consequence.	Comparable to placebo; organ toxicity not increased. Overall well tolerated despite high immunogenicity.
Other Targeted mAbs	1219	Common: infusion reactions and transient cytokine shifts. SAEs: none dose-limiting, no cytokine storm, and no ↑ secondary infections.	Equal to placebo in all trials. No new major safety signal. Checkpoint inhibitor and complement blockade considered safe in selected patients.
Polyclonal IVIG	124	Common: infusion reactions (headache, flushing, and fever) in 5–15%. SAEs: none consistent (no ↑ thrombosis, renal failure, or infections).	No difference vs. placebo in SAE incidence.
IgM-enriched IVIG	669	Common: transient fever, mild allergic reactions, and headache. SAEs: none reported.	Safety comparable to placebo. No signals of added harm.

Abbreviations: mAb: monoclonal antibody, IVIG: intravenous immunoglobulin, SAE: serious adverse event, HAMA: human anti–mouse antibody, TNF: Tumor Necrosis Factor, ↑ indicates an increase in antibody formation.

## Data Availability

No new data were created. All results are based on data extracted from previously published randomized controlled trials, which are cited in the manuscript.

## Supplementary Materials

The following supporting information can be downloaded at: https://www.mdpi.com/article/10.3390/ijms26188859/s1. The review protocol was prospectively registered in PROSPERO (registration number CRD420251073312) and is freely accessible at https://www.crd.york.ac.uk/PROSPERO/view/CRD420251073312 (accessed on 13 June 2025). Supplementary Material S1 presents the database-specific search strategies, including the complete search strings. The strategies were developed, structured, and documented using Review Manager (RevMan Web) (The Cochrane Collaboration, 2024; available at https://revman.cochrane.org, accessed on 13 June 2025). Supplementary Material S2 provides the detailed assessment of this systematic review using the AMSTAR-2 (A Measurement Tool to Assess Systematic Reviews). Supplementary Material S3 lists all studies excluded during the screening and eligibility assessment phases of this review. For transparency, each entry includes the study title, the primary reason for exclusion, and the corresponding DOI. Supplementary Material S4 presents the Summary of Findings (SoF) table for this systematic review, developed according to the GRADE framework. The table was produced using RevMan Web.
