# Supplementary material for: Assessing Monoclonal and Polyclonal Antibodies in Sepsis and Septic Shock: A Systematic Review of Efficacy and Safety
Source: Int J Mol Sci. 2025 Sep 11;26(18):8859. doi: 10.3390/ijms26188859 (PMC12469444; doi:10.3390/ijms26188859)
Supplement: Supplementary file 1 [file ijms-26-08859-s001.zip › Supplementary Material 1. Full Search Strategy.pdf]

Search strategies

Database-Specific Search Strategies - Full Search Strings

The search was performed across the following online databases: MEDLINE, Web of Science, Google Scholar, Embase, and the Cochrane Library (CENTRAL). The research has been done both in "free text","subject headings" (Medical Subject Headings - MeSH or EMTree) and keywords were employed in the search strategy. The syntax will encompass the terms of Antibody (OR "Antibodies" OR "Monoclonal Antibodies" OR "Polyclonal Antibodies" OR "Ab" OR "Humanized Antibodies" OR "Bispecific Antibodies" OR "Antibody-Drug" OR "Immunoglobulin" OR "Immunoglobulins" OR "Immunoglobulin" OR "Immune Globulins" OR "lmmunomodulatory") and Sepsis (OR "Septic shock" OR "Septicemia" OR "Bacteraemia" OR "Bacteremia" OR "Bacteriemia" OR "Bacterial infections" OR "Bacterial infection" OR "Bacterial-infections" OR "Systemic Inflammatory Response Syndrome" OR "SIRS" OR "Endotoxemia" OR "Multiple Organ Failure" OR "Organ dysfunction" OR "Cytokine Release Syndrome") and Treatment (OR "Therapy" OR "Therapeutic" OR "Care" OR "Intervention" OR "Effect" OR "Outcome" OR "Safety" OR "Management") and RCT (OR "RCTs OR "Randomized Controlled Trial" OR "Controlled Trial" OR "Controlled" OR "Trial" OR "blind" OR "Placebo").

Sources and search methods

Electronic searches: core databases

Database	Search Period
CENTRAL	17.09.2024 - 28.02.2025 (weekly updated)
MEDLINE (Ovid)	01.10.2024 - 28.02.2025 (weekly updated)
EMBASE (Ovid)	13.11.2024 - 28.02.2025 (weekly updated)
Web of Science	18.11.2024 - 28.02.2025 (weekly updated)
Scopus	02.12.2024 - 28.02.2025 (weekly updated)

MEDLINE search strategy

1. exp Antibodies/
2. (antibody or antibodies or monoclonal antibody* or polyclonal antibody* or ab or humanized antibodies or bispecific antibodies or antibody-drug or immunoglobulin" or immune globulins or immunomodulatory).mp.
3. or/1-2

4. exp Sepsis/
5. (sepsis or septic shock or septicemia or bacteraemia or bacteremia or bacteriemia or bacterial infection* or systemic inflammatory response syndrome or SIRS or endotoxemia or multiple organ failure or organ dysfunction or cytokine release

syndrome).mp.
6. or/4-5

7. (therapy or therapeutic or care or intervention or effect or outcome or safety or management).mp.

8. ("randomized controlled trial" or RCT or "randomized controlled trial" or "controlled trial" or controlled or trial or blind or placebo).ab,ti.

9. adult.mp. or exp Adult/
10. (adult* or critically ill).ti,ab.

11. OF/9-10

12. 3 and 6 and 7 and 8 and 11

13. exclude animal studies: exp Animals/ not Humans/

14. 12 not 13

Filter to identify RCTs

1. exp "clinical trial [publication type]"/

2. (randomized or randomised).ab,ti.

3. placebo.ab,ti.

4. dt.Is.

5. randomly.ab,ti.

6. trial.ab,ti.

T. groups.ab,ti.

8. or/1-7

9. Animals/

10. Humans/

11. 9 not (9 and 10)

12. 8 not 11

The MEDLINE strategy and RCT filter are adapted to identify trials in other electronic databases.

CENTRAL search strategy

#1 MeSH descriptor: [Antibodies] explode all trees OR "antibodies":ti,ab OR "monoclonal antibodies":ti,ab OR "polyclonal antibodies":ti,ab OR "ab":ti,ab OR "humanized antibodies":ti,ab OR "bispecific antibodies":ti,ab OR "antibody-drug":ti,ab OR "immunoglobulin*":ti,ab OR "immune globulins":ti,ab OR "immunomodulatory":ti,ab

#2 MeSH descriptor: [Sepsis] explode all trees OR sepsis:ti,ab OR "septic shock":ti,ab OR septicemia:ti,ab OR bacteraemia:ti,ab OR bacteremia:ti,ab OR bacteriemia:ti,ab OR "bacterial infection*":ti,ab OR "systemic inflammatory response syndrome":ti,ab OR SIRS:ti,ab OR endotoxemia:ti,ab OR "multiple organ failure":ti,ab OR "organ dysfunction":ti,ab OR "cytokine release syndrome":ti,ab

#3 therapy:ti,ab OR therapeutic:ti,ab OR care:ti,ab OR intervention:ti,ab OR effect:ti,ab OR outcome:ti,ab OR safety:ti,ab OR management:ti,ab

#4 "randomized controlled trial":ti,ab OR rct:ti,ab OR "controlled trial":ti,ab OR controlled:ti,ab OR trial:ti,ab OR blind:ti,ab OR placebo:ti,ab

#5 adult:ti,ab OR "critically ill":ti,ab

#6#1AND#2AND#3AND#4AND#5

[Note: in search line #1, MISC1 denotes the field in the record where the reference has been coded for condition, in this case, Sepsis]

EMBASE search strategy

1. 'monoclonal antibody'/exp OR 'polyclonal antibody'/exp OR antibody:ti,ab OR antibodies:ti,ab OR monoclonal antibody*:ti,ab OR polyclonal antibody*:ti,ab OR ab:ti,ab OR humanized antibodies:ti,ab OR bispecific antibodies:ti,ab OR 'antibody-drug".ti,ab OR immunoglobulin*:ti,ab OR 'immune globulins".ti,ab OR immunomodulatory:ti,ab
2. or/1

3. 'sepsis'/exp OR sepsis:ti,ab OR 'septic shock':ti,ab OR septicemia:ti,ab OR bacteraemia:ti,ab OR bacteremia:ti,ab OR bacteriemia:ti,ab OR 'bacterial infection"".ti,ab OR 'systemic inflammatory response syndrome".ti,ab OR sirs:ti,ab OR endotoxemia:ti,ab OR 'multiple organ failure".ti,ab OR 'organ dysfunction".ti,ab OR 'cytokine release syndrome':ti,ab
4. or/3

5. therapy:ti,ab OR therapeutic:ti,ab OR care:ti,ab OR intervention:ti,ab OR effect:ti,ab OR outcome:ti,ab OR safety:ti,ab OR management:ti,ab

6. 'randomized controlled trial'/exp OR rct:ti,ab OR 'randomized controlled trial".ti,ab OR 'controlled trial".ti,ab OR controlled:ti,ab OR trial:ti,ab OR blind:ti,ab OR placebo:ti,ab
7. or/6

8. 'adult'/exp OR adult:ti,ab OR 'critically ill':ti,ab
9. or/8

10.2 and 4 and 5 and 7 and 9

Web of Science search strategy

TS=("antibody" OR "antibodies" OR "monoclonal antibodies" OR "polyclonal antibodies" OR ab OR "humanized antibodies" OR "bispecific antibodies" OR "antibody-drug" OR immunoglobulin* OR "immune globulins" OR immunomodulatory)
AND
TS=(sepsis OR "septic shock" OR septicemia OR bacteraemia OR bacteremia OR bacteriemia OR "bacterial infection"" OR "systemic inflammatory response syndrome" OR sirs OR endotoxemia OR "multiple organ failure" OR "organ dysfunction" OR "cytokine release syndrome")
AND
TS=(therapy OR therapeutic OR care OR intervention OR effect OR outcome OR safety OR management)
AND
TS=(RCT OR "randomized controlled trial" OR "controlled trial" OR controlled OR trial OR blind OR placebo)

Scopus search strategy

TITLE-ABS-KEY(("antibody" OR "antibodies" OR "monoclonal antibodies" OR "polyclonal antibodies" OR ab OR "humanized antibodies" OR "bispecific antibodies" OR "antibody-drug" OR immunoglobulin* OR "immune globulins" OR immunomodulatory)
AND
(sepsis OR "septic shock" OR septicemia OR bacteraemia OR bacteremia OR bacteriemia OR "bacterial infection"" OR "systemic inflammatory response syndrome" OR sirs OR endotoxemia OR "multiple organ failure" OR "organ dysfunction" OR "cytokine release syndrome")
AND
(therapy OR therapeutic OR care OR intervention OR effect OR outcome OR safety OR management)
AND
(rct OR "randomized controlled trial" OR "controlled trial" OR controlled OR trial OR blind OR placebo)
