# Supplementary material for: Assessing Monoclonal and Polyclonal Antibodies in Sepsis and Septic Shock: A Systematic Review of Efficacy and Safety
Source: Int J Mol Sci. 2025 Sep 11;26(18):8859. doi: 10.3390/ijms26188859 (PMC12469444; doi:10.3390/ijms26188859)
Supplement: Supplementary file 1 [file ijms-26-08859-s001.zip › supplementary material 3.pdf]

List of excluded studies

Supplementary Material 3: Studies excluded from the systematic review, provided in accordance with AMSTAR-2 guidelines, based on the predefined inclusion and exclusion criteria. The table presents the authors, full citation, and reason for exclusion.

Authors	Title	Reason of exclusion
Abraham et al. [1]	P55 Tumor Necrosis Factor Receptor Fusion Protein in the Treatment of Patients with Severe Sepsis and Septic Shock: A Randomized Controlled Multicenter Trial.	**Inappropriate intervention** (not a mAb or pAb)
Aitchison et al. [2]	Anti-endotoxin in the treatment of severe surgical septic shock: results of a randomized double-blind trial.	**Inappropriate patient population** (the patient age group was not clearly defined). **Study design not conforming to eligibility criteria** (study failed to clearly define septic shock or organ dysfunction using standardized diagnostic criteria)
Biagioni et al. [3]	Adjunctive IgM-enriched immunoglobulin therapy with a personalised dose based on serum IgM-titres versus standard dose in the treatment of septic shock: a randomised controlled trial (IgM-fat trial).	**Outcomes not aligned with review objectives** (Comparator was a different dosing regimen of the same intervention, not placebo or standard care)
Burns et al. [4]	A. Treatment of septic thrombocytopenia with immune globulin.	**Study population has different clinical indication** (restricted to septic patients with acute thrombocytopenia; primary endpoint focused on platelet recovery, not general sepsis outcomes)
Calandra et al. [5]	Treatment of gram-negative septic shock with human IgG antibody to Escherichia coli J5: a prospective, double-blind, randomized trial.	**Inappropriate comparator** (comparator was another immunoglobulin preparation, not placebo or standard sepsis treatment). **Inappropriate patient population** (children are also included)
Clark et al. [6]	Effect of a chimeric antibody to tumor necrosis factor-alpha on cytokine and physiologic responses in patients with severe sepsis - a randomized, clinical trial.	**Outcomes not aligned with review objectives** (Primary outcomes were cytokine profiles, metabolic and fluid balance parameters)

Dominioni et al. [7]	Effects of high-dose IgG on survival of surgical patients with sepsis scores of 20 or greater.	**Inappropriate patient population** (individuals under 18 years were also included)
Dominioni et al. [8]	High-Dose Intravenous IgG for Treatment of Severe Surgical Infections.	**Inappropriate patient population** (individuals under 18 years were also included)
Domizi et. al. [9]	IgM-enriched immunoglobulins (Pentaglobin) may improve the microcirculation in sepsis: a pilot randomized trial.	**Outcomes not aligned with review objectives** (primary outcomes assess microvascular perfusion and cytokine levels)
François et al. [10]	Safety and tolerability of a single administration of AR-301, a human monoclonal antibody, in ICU patients with severe pneumonia caused by Staphylococcus aureus: first-in-human trial.	**Inappropriate setting** (not primarily address sepsis or septic shock, but severe pneumonia)
François et al. [11]	COMBACTE Consortium and the SAATELLITE Study Group. Efficacy and safety of suvratoxumab for prevention of Staphylococcus aureus ventilator-associated pneumonia (SAATELLITE): a multicentre, randomised, double-blind, placebo-controlled, parallel-group, phase 2 pilot trial.	**Inappropriate setting** (study population did not include patients with active sepsis or septic shock)
Geven et al. [12]	A double-blind, placebo-controlled, randomised, multicentre, proof-of-concept and dose-finding phase II clinical trial to investigate the safety, tolerability and efficacy of adrecizumab in patients with septic shock and elevated adrenomedullin concentration (AdrenOSS-2).	**Population overlaps with included studies** (the Phase IIa study by Laterre et al., 2021 is already included in this systematic review)
Greenberg et al. [13]	Observations using antiendotoxin antibody (E5) as adjuvant therapy in humans with suspected, serious, gram-negative sepsis.	**Study design not conforming to eligibility criteria** (study failed to clearly define septic shock or organ dysfunction)
Grundmann et al. [14]	Immunoglobulin therapy in patients with endotoxemia and postoperative sepsis - a prospective randomized study.	**Population overlaps with included studies** (the RCT study by Wesoly et. al., 1990 is already included in this systematic review)
Hentrich et al. [15]	IgMA-enriched immunoglobulin in neutropenic patients with sepsis syndrome and septic shock: a randomized, controlled, multiple-center trial.	**Inappropriate patient population** (neutropenic patients with active cancer)
Jaspers et al. [16]	Antibody to lipoid A in the treatment of septic shock [Antikörper gegen Lipoid A in der Behandlung des septischen Schocks].	**Study design not conforming to eligibility criteria** (the study is not a randomized controlled trial)
Just et al. [17]	Einfluss einer adjuvanten Immunglobulintherapie auf Infektionen bei Patienten einer operativen Intensiv-Therapie-	**Inappropriate patient population** (the study

	Station [Effect of adjuvant immunoglobulin therapy on infections in patients in an surgical intensive care unit. Results of a randomized controlled study].	included children and neonates and not all patients under investigation had sepsis or septic shock)
Kett et al. [18]	An evaluation of the hemodynamic effects of HA-1A human monoclonal antibody.	**Outcomes not aligned with review objectives** (focused on the effect of HA-1A on hemodynamic changes). **Study design not conforming to eligibility criteria** (post-hoc analysis of an RCT rather than a primary randomized controlled trial.)
Lindquist et al. [19]	Pepsin-treated human gamma globulin in bacterial infections. A randomized study in patients with septicaemia and pneumonia.	**Inappropriate patient population** (Included patients were not limited to those with sepsis or septic shock) **Study design not conforming to eligibility criteria** (study failed to clearly define septic shock or organ dysfunction
McCarthy MW. [20]	Optimizing the use of vilobelimab for the treatment of COVID-19.	**Inappropriate patient population** (Included patients had COVID-19 disease but were not necessarily diagnosed with sepsis or septic shock attributable to COVID-19)
Schedel et al. [21]	Treatment of gram-negative septic shock with an immunoglobulin preparation: a prospective, randomized clinical trial.	**Inappropriate patient population** (individuals under 18 years were also included)
Tugrul et al. [22]	The effects of IgM-enriched immunoglobulin preparations in patients with severe sepsis [ISRCTN28863830].	**Inappropriate patient population** (individuals under 18 years were also included)
Vlaar et al. [23]	Anti-C5a antibody IFX-1 (vilobelimab) treatment versus best supportive care for patients with severe COVID-19 (PANAMO): an exploratory, open-label, phase 2 randomised controlled trial.	**Inappropriate patient population** (included patients had severe pneumonia due to COVID-19 but did not necessarily meet criteria for sepsis or septic shock)
Wortel et al. [24]	Effectiveness of a human monoclonal anti-endotoxin antibody (HA-1A) in gram-negative sepsis: relationship to endotoxin	**Population overlaps with included studies** (the RCT

	and cytokine levels.	study by Ziegler et. al. 1991 is already included in this systematic review)
Abraham et al. [25]	Drotrecogin alfa (activated) for adults with severe sepsis and a low risk of death.	**Inappropriate intervention** (drotrecogin alfa, a recombinant activated protein C, not a monoclonal or polyclonal antibody)
Bone et al. [26]	A controlled clinical trial of high-dose methylprednisolone in the treatment of severe sepsis and septic shock.	**Inappropriate intervention** (methylprednisolone, not a monoclonal or polyclonal antibody)
Brocklehurst et al. [27]	INIS Collaborative Group. Treatment of neonatal sepsis with intravenous immune globulin.	**Inappropriate patient population** (neonates with suspected or proven sepsis)
Fisher et al. [28]	Treatment of septic shock with the tumor necrosis factor receptor:Fc fusion protein. The Soluble TNF Receptor Sepsis Study Group.	**Inappropriate intervention** (soluble TNF receptor fusion protein, not a monoclonal or polyclonal antibody)
Fisher et al. [29]	Initial evaluation of human recombinant interleukin-1 receptor antagonist in the treatment of sepsis syndrome: a randomized, open-label, placebo-controlled multicenter trial.	**Inappropriate intervention** (interleukin-1 receptor antagonist, not mAb or pAb)
Reinhart et al. [30]	Anti-tumor necrosis factor therapy in sepsis: update on clinical trials and lessons learned.	**Inappropriate intervention** (the study is a review of anti-TNF therapies)
Fisher et al. [31]	Influence of an anti-tumor necrosis factor monoclonal antibody on cytokine levels in patients with sepsis. The CB0006 Sepsis Syndrome Study Group.	**Study design not conforming to eligibility criteria** (open-label phase II trial not a randomized, double-blind controlled trial)
Kaul et al. [32]	Intravenous immunoglobulin therapy for streptococcal toxic shock syndrome - a comparative observational study. The Canadian Streptococcal Study Group.	**Study design not conforming to eligibility criteria** (observational comparative study rather than a randomized controlled trial)
Leeies et al. [33]	Intravenous immune globulin in septic shock: a Canadian national survey of critical care medicine and infectious disease specialist physicians.	**Study design not conforming to eligibility criteria** (cross-sectional survey of physician practices and perceptions, not a randomized controlled trial)
Laupland et al. [34]	Polyclonal intravenous immunoglobulin for the prophylaxis and treatment of infection in critically ill adults.	**Study design not conforming to eligibility criteria** (review article, not a primary randomized controlled trial)

Hung et al. [35]	Hyperimmune IV immunoglobulin treatment: a multicenter double-blind randomized controlled trial for patients with severe 2009 influenza A(H1N1) infection.	**Study population has different clinical indication** (patients with severe influenza infection, rather than sepsis or septic shock)
Kalvelage et al. [36]	Personalized medicine with IgGAM compared with standard of care for treatment of peritonitis after infectious source control (the PEPPER trial): study protocol for a randomized controlled trial.	**Study design not conforming to eligibility criteria** (it is a study protocol for a Phase III RCT still in the recruiting phase and not yet published)
Levin et al. [37]	Recombinant bactericidal/permeability-increasing protein (rBPI21) as adjunctive treatment for children with severe meningococcal sepsis: a randomised trial.	**Inappropriate patient population** (children rather than the adult population)
Ohlsson et al. [38]	Intravenous immunoglobulin for preventing infection in preterm and/or low birth weight infants.	**Inappropriate patient population** (preterm and/or low birth weight infants rather than the adult population)
Migone et al. [39]	Raxibacumab for the treatment of inhalational anthrax.	**Study design not conforming to eligibility criteria** (efficacy data come only from animal studies, no data for sepsis or septic shock)
Tocut et al. [40]	Outcomes of ICU patients treated with intravenous immunoglobulin for sepsis or autoimmune diseases.	**Study design not conforming to eligibility criteria** (retrospective observational study rather than a randomized controlled trial)
Scarpati et al. [41]	Effect of Intravenous IgM-Enriched Immunoglobulins on Presepsin and Other Sepsis Biomarkers.	**Study design not conforming to eligibility criteria** (observational cohort study not an RCT)
Derkx et al. [42]	Randomized, placebo-controlled trial of HA-1A, a human monoclonal antibody to endotoxin, in children with meningococcal septic shock. European Pediatric Meningococcal Septic Shock Trial Study Group.	**Inappropriate patient population** (involved a pediatric population)
Lee et al. [43]	Impact of polymyxin B hemoperfusion therapy on high endotoxin activity level patients after successful infection source control: a prospective cohort study.	**Study design not conforming to eligibility criteria** (not an RCT, no evaluation of mAbs or pAbs)
Senda et al. [44]	Effectiveness of intravenous immunoglobulin therapy for invasive group A Streptococcus infection: A Japanese nationwide observational study.	**Study design not conforming to eligibility criteria** (retrospective observational, not a

		randomized controlled trial)
Akdag et al. [45]	Role of pentoxifylline and/or IgM-enriched intravenous immunoglobulin in the management of neonatal sepsis.	**Inappropriate patient population** (involves neonates rather than adults)
Elgendy et al. [46]	Effect of Pentoxifylline on Organ Dysfunction and Mortality in Severe Sepsis.	**Inappropriate intervention** (pentoxifylline under investigation so not a mAb or pAb)
Aggarwal et al. [47]	Efficacy of high-dose intravenous immunoglobulin in severe and critical COVID-19: A retrospective cohort study.	**Study design not conforming to eligibility criteria** (retrospective cohort, not a randomized controlled trial, patients with severe COVID-19 but no incidences of severe sepsis or septic shock)
Klein et al. [48]	Polymyxin B hemoperfusion in endotoxemic septic shock patients without extreme endotoxemia: a post hoc analysis of the EUPHRATES trial.	**Study design not conforming to eligibility criteria** (post hoc analysis of an existing trial)
Choi et al. [49]	Adjuvant intravenous immunoglobulin administration on postoperative critically ill patients with secondary peritonitis: a retrospective study.	**Study design not conforming to eligibility criteria** (retrospective observational study, not a randomized controlled trial)
Moughames et al. [50]	Outcomes of intravenous immunoglobulin treatment of immunocompromised patients with viral respiratory infections.	**Study design not conforming to eligibility criteria** (observational retrospective cohort study, rather than a randomised controlled trial, involves patients with viral respiratory tract infections, but not sepsis or septic shock)
Derhaschnig et al. [51]	Recombinant human activated protein C (rhAPC; drotrecogin alfa [activated]) has minimal effect on markers of coagulation, fibrinolysis, and inflammation in acute human endotoxemia.	**Inappropriate intervention** (Recombinant human activated protein C is not a monoclonal or polyclonal antibody, volunteers with experimental endotoxemia rather than patients with sepsis or septic shock)
Portefaix et al. [52]	High-dose intravenous immunoglobulin versus albumin 4% in paediatric toxic shock syndrome: a randomised controlled feasibility study.	**Inappropriate patient population** (children rather than the adult population)
Presneill et al. [53]	A randomized phase II trial of granulocyte-macrophage	**Inappropriate intervention**

	colony-stimulating factor therapy in severe sepsis with respiratory dysfunction.	(granulocyte-macrophage colony-stimulating factor not a mAb or pAb)
Cometta et al. [54]	Prophylactic intravenous administration of standard immune globulin as compared with core-lipopolysaccharide immune globulin in patients at high risk of postsurgical infection.	**Study Population has different clinical Indication** (pAb was administered as a prophylaxis to high-risk postsurgical patients without diagnosed sepsis or septic shock)
McCulloch et al. [55]	Treatment with IgM-enriched intravenous immunoglobulins enhances clearance of stroke-associated bacterial lung infection.	**Inappropriate setting** (animal model experiment)
Welte et al. [56]	Concept for a Study Design in Patients with Severe Community-Acquired Pneumonia: A Randomised Controlled Trial with a Novel IGM-Enriched Immunoglobulin Preparation – the CIGMA Study.	**Study population has different clinical indication** (focuses on patients with severe community-acquired pneumonia, it does not provide clear diagnosis or confirmation of sepsis or septic shock)
Singer et al. [57]	The immunomodulating activity of trimodulin (polyvalent IgM, IgA, IgG solution): a post hoc analysis of the phase II CIGMA trial.	**Study design not conforming to eligibility criteria** (post hoc analysis rather than a primary randomized controlled trial)
Farkhutdinov et al. [58]	Intravenous immunoglobulin in community acquired pneumonia.	**Study population has different clinical indication** (patients under investigation have community-acquired pneumonia but do not have sepsis or septic shock)
Rupp et al. [59]	Phase II, randomized, multicenter, double-blind, placebo-controlled trial of a polyclonal anti-Staphylococcus aureus capsular polysaccharide immune globulin in treatment of Staphylococcus aureus bacteremia.	**Study population has different clinical indication** (focuses on patients with Staphylococcus aureus bacteremia rather than patients with sepsis or septic shock)
Agafina et al. [60]	Efficacy and safety of trimodulin in patients with severe COVID-19: results from a randomised, placebo-controlled, double-blind, multicentre, phase II trial (ESsCOVID).	**Study population has different clinical indication** (focuses on adults hospitalized with severe COVID-19 pneumonia requiring non-invasive ventilation or high-flow oxygen, not on patients with

		sepsis or septic shock)
Newham et al. [61]	Determination of the safety and efficacy of therapeutic neutralization of tumor necrosis factor-α (TNF-α) using AZD9773, an anti-TNF-α immune Fab, in murine CLP sepsis.	**Inappropriate setting** (preclinical animal study)
Newham et al. [62]	AZD9773, a novel anti-TNFα immune Fab in development for severe sepsis and septic shock: demonstration of safety and efficacy in a murine CLP sepsis model.	**Inappropriate setting** (preclinical murine CLP sepsis model study)
Chen et al. [63]	Safety, Tolerability, Pharmacokinetics, and Pharmacodynamics of Anti-C5a Antibody BDB-001 for Severe COVID-19: A Randomized, Double-Blind, Placebo-Controlled Phase 1 Clinical Trial in Healthy Chinese Adults.	**Inappropriate patient population** (healthy volunteers, not in patients with sepsis or septic shock)
Rizvi et al. [64]	Intravenous immunoglobulin in the management of neonatal sepsis: A randomised controlled trial.	**Inappropriate patient population** (neonatal populations)
Shenoi et al. [65]	Multicenter randomized placebo-controlled trial of therapy with intravenous immunoglobulin in decreasing mortality due to neonatal sepsis.	**Inappropriate patient population** (neonatal populations)
Horspool et al. [66]	Development of an anti-*Pseudomonas aeruginosa* therapeutic monoclonal antibody WVDC-5244.	**Inappropriate setting** (preclinical study involving murine models)
Sandberg et al. [67]	Preterm infants with low immunoglobulin G levels have increased risk of neonatal sepsis but do not benefit from prophylactic immunoglobulin G.	**Inappropriate patient population** (preterm neonates with low IgG levels)
Fang et al. [68]	Monoclonal antibodies to endotoxin in the management of sepsis.	**Study design not conforming to eligibility criteria** (primarily a review of sepsis nomenclature)
Cross et al. [69]	A pilot study of an anti-endotoxin Ig-enriched bovine colostrum to prevent experimental sepsis.	**Inappropriate setting** (preclinical animal study)
Soares et al. [70]	An evaluation of the feasibility, cost and value of information of a multicentre randomised controlled trial of intravenous immunoglobulin for sepsis (severe sepsis and septic shock): incorporating a systematic review, meta-analysis and value of information analysis.	**Study design not conforming to eligibility criteria** (meta-analysis)
Soares et al. [71]	Intravenous immunoglobulin for severe sepsis and septic shock: clinical effectiveness, cost-effectiveness and value of a further randomised controlled trial.	**Outcomes not aligned with review objectives** (study's outcomes are economic and modeling-focused rather than the clinical endpoints)
Akatsuka et al. [72]	Efficacy of Intravenous Immunoglobulin Therapy for Patients With Sepsis and Low Immunoglobulin G Levels: A Single-Center Retrospective Study.	**Study design not conforming to eligibility criteria** (retrospective observational study, not a randomized controlled trial)
Ando et al. [73]	Intravenous Immunoglobulin G Modulates the Expression of Sepsis-Induced Coagulopathy Factors and Increases Serum	**Study Design Not Conforming to Eligibility**

	IgM Levels: A Prospective, Single-Center Intervention Study.	**Criteria** (prospective interventional study, main outcomes focus on biomarkers and coagulopathy factors) **Inappropriate Comparator** (compares groups based on serum IgG levels)
Goto et al. [74]	Effects of low-dose intravenous immunoglobulin as the adjunctive therapy in septic shock patients with and without hypogammaglobulinemia: a retrospective cohort study.	**Study design not conforming to eligibility criteria** (non-RCT, retrospective cohort design)
Stephens et al. [75]	Randomized, double-blind, placebo-controlled trial of granulocyte colony-stimulating factor in patients with septic shock.	**Inappropriate intervention** (granulocyte colony-stimulating factor not a mAb or pAb)
Behre et al. [76]	Endotoxin concentrations and therapy with polyclonal IgM-enriched immunoglobulins in neutropenic cancer patients with sepsis syndrome: pilot study and interim analysis of a randomized trial.	**Inappropriate patient population** (neutropenic patients with active cancer)
Buda et al. [77]	Clinical experience with polyclonal IgM-enriched immunoglobulins in a group of patients affected by sepsis after cardiac surgery.	**Study design not conforming to eligibility criteria** (retrospective case-controlled study)
Sakka, S.G. [78]	Sepsis bei einer Patientin mit Autoimmunerkrankung und immunsuppressiver Therapie – Einsatz eines polyvalenten IgGAM-Präparates	**Study design not conforming to eligibility criteria** (not-RCT)
Vogel et al. [79]	Bewertung der intravenösen IgM-Therapie bei schweren nosokomialen Infektionen (Ergebnis einer kontrollierten randomisierten Studie).	**Study design not conforming to eligibility criteria** (not-RCT)
Yavuz et al. [80]	The effects of adjuvant immunoglobulin M-enriched immunoglobulin therapy on mortality rate and renal function in sepsis-induced multiple organ dysfunction syndrome: retrospective analysis of intensive care unit patients.	**Study design not conforming to eligibility criteria** (retrospective study)
Kakoullis et al. [81]	The use of IgM-enriched immunoglobulin in adult patients with sepsis.	**Study design not conforming to eligibility criteria** (review article, not-RCT)
Masaoka T. [82]	[Combination therapy of antibiotics and intravenous immunoglobulin].	**Study population has different clinical indication** (patients with severe infections, not defined as sepsis or septic shock with organ dysfunction)
Opal et al. [83]	Confirmatory interleukin-1 receptor antagonist trial in severe sepsis: a phase III, randomized, double-blind, placebo-controlled, multicenter trial. The Interleukin-1 Receptor	**Inappropriate intervention** (interleukin-1 receptor antagonist, not mAb or pAb)

	Antagonist Sepsis Investigator Group.	
Dhainaut et al. [84]	Platelet-activating factor receptor antagonist BN 52021 in the treatment of severe sepsis: a randomized, double-blind, placebo-controlled, multicenter clinical trial. BN 52021 Sepsis Study Group.	**Inappropriate intervention** (platelet-activating factor receptor antagonist, not mAb or pAb)
Dries et al. [85]	Effect of interferon gamma on infection-related death in patients with severe injuries. A randomized, double-blind, placebo-controlled trial.	**Study population has different clinical indication** (severely injured trauma patients, not sepsis or septic shock)
Sallam et al. [86]	A novel combination approach of human polyclonal IVIG and antibiotics against multidrug-resistant Gram-positive bacteria.	**Study design not conforming to eligibility criteria** (in vitro and murine model study, not RCT in human patients)
Stuttmann et al. [87]	Prophylaxe mit einem Pseudomonas-Immunglobulin bei Brandverletzten [Prevention using a Pseudomonas immunoglobulin in burn patients].	**Study population has different clinical indication** (severely burned patients, not sepsis or septic shock)
Hansbrough et al. [88]	High dose intravenous immunoglobulin therapy in burn patients: pharmacokinetics and effects on microbial opsonization and phagocytosis.	**Study population has different clinical indication** (severe burn patients, not sepsis or septic shock)
Pilz et al. [89]	Supplemental immunoglobulin (ivIgG) treatment in 163 patients with sepsis and septic shock--an observational study as a prerequisite for placebo-controlled clinical trials. Infection.	**Study design not conforming to eligibility criteria** (observational study, not RCT)
Cavazzuti et al. [90]	Early therapy with IgM-enriched polyclonal immunoglobulin in patients with septic shock.	**Study design not conforming to eligibility criteria** (retrospective study, not RCT)
Hamano et al. [91]	Efficacy of single-dose intravenous immunoglobulin administration for severe sepsis and septic shock.	**Inappropriate comparator** (divided vs. single-dose IVIG, does not reflect a standard care or placebo)
Tagami et al. [92]	Intravenous immunoglobulin use in septic shock patients after emergency laparotomy.	**Study design not conforming to eligibility criteria** (observational database study, not RCT)
Tagami et al. [93]	Intravenous immunoglobulin and mortality in pneumonia patients with septic shock: an observational nationwide study.	**Study design not conforming to eligibility criteria** (observational study, not RCT)
Sakoulas et al. [94]	Intravenous Immunoglobulin (IVIG) Significantly Reduces Respiratory Morbidity in COVID-19 Pneumonia: A Prospective Randomized Trial.	**Study population has different clinical indication** (COVID-19 pneumonia, not sepsis or septic shock)

Hellerud et al. [95]	Combined inhibition of C5 and CD14 efficiently attenuated the inflammatory response in a porcine model of meningococcal sepsis.	**Inappropriate setting** (animal study)
Stevens et al. [96]	Therapeutic targeting of HMGB1 during experimental sepsis modulates the inflammatory cytokine profile to one associated with improved clinical outcomes.	**Inappropriate setting** (animal study)
Wang et al. [97]	Therapeutic potential of HMGB1-targeting agents in sepsis.	**Inappropriate setting** (review article)
Warren et al. [98]	Assessment of ability of murine and human anti-lipid A monoclonal antibodies to bind and neutralize lipopolysaccharide.	**Study design not conforming to eligibility criteria** (in vitro study, not RCT in human patients)
Waymack et al. [99]	A prospective trial of prophylactic intravenous immune globulin for the prevention of infections in severely burned patients.	**Study population has different clinical indication** (severely burned patients)
Pedraza-Sánchez et al. [100]	Polyvalent human immunoglobulin for infectious diseases: Potential to circumvent antimicrobial resistance.	**Study design not conforming to eligibility criteria** (review article, not RCT)
Weisman et al. [101]	Phase 1/2 double-blind, placebo-controlled, dose escalation, safety, and pharmacokinetic study of pagibaximab (BSYX-A110), an antistaphylococcal monoclonal antibody for the prevention of staphylococcal bloodstream infections, in very-low-birth-weight neonates.	**Inappropriate patient population** (very-low-birth-weight neonates)
Weisman et al. [102]	A randomized study of a monoclonal antibody (pagibaximab) to prevent staphylococcal sepsis.	**Inappropriate patient population** (very-low-birth-weight neonates)
Ishikura et al. [103]	Intravenous immunoglobulin improves sepsis-induced coagulopathy: A retrospective, single-center observational study.	**Study design not conforming to eligibility criteria** (retrospective observational study, not RCT)
Perrella et al. [104]	Sepsis Outcome after Major Abdominal Surgery Does Not Seem to Be Improved by the Use of Pentameric Immunoglobulin IgM: A Single-Center Retrospective Analysis.	**Study design not conforming to eligibility criteria** (retrospective analysis, not RCT)
Dimaano et al. [105]	Lack of efficacy of high-dose intravenous immunoglobulin treatment of severe thrombocytopenia in patients with secondary dengue virus infection.	**Inappropriate patient population** (patients with secondary dengue virus infection, not sepsis or septic shock)
Takahashi et al. [106]	Study of usefulness of low-dose IgG for patients with septic disseminated intravascular coagulation.	**Study design not conforming to eligibility criteria** (retrospective study)
Takano et al. [107]	Intravenous immunoglobulin for mortality and inflammatory status in patients with sepsis: a retrospective database study.	**Study design not conforming to eligibility criteria** (retrospective cohort

		study)
Baker et al. [108]	Intravenous immune globulin for the prevention of nosocomial infection in low-birth-weight neonates. The Multicenter Group for the Study of Immune Globulin in Neonates.	**Inappropriate patient population** (neonates/low-birth-weight infants)
Fanaroff et al. [109]	A controlled trial of intravenous immune globulin to reduce nosocomial infections in very-low-birth-weight infants. National Institute of Child Health and Human Development Neonatal Research Network.	**Inappropriate patient population** (very-low-birth-weight infants/neonates)
Bernard et al. [110]	Recombinant human protein C Worldwide Evaluation in Severe Sepsis (PROWESS) study group. Efficacy and safety of recombinant human activated protein C for severe sepsis.	**Inappropriate intervention** (recombinant human activated protein C, not mAb or pAb)
Savva et al. [111]	Targeting toll-like receptors: promising therapeutic strategies for the management of sepsis-associated pathology and infectious diseases.	**Study design not conforming to eligibility criteria** (not RCT)
Palliyil et al. [112]	Monoclonal Antibodies Targeting Surface-Exposed Epitopes of Candida albicans Cell Wall Proteins Confer In Vivo Protection in an Infection Model.	**Inappropriate setting** (animal study)
Rosario-Colon et al. [113]	Monoclonal antibodies targeting Candida disrupt biofilms and inhibit growth across global clinical isolates.	**Inappropriate setting** (animal study)
Rello et al. [114]	A randomized placebo-controlled phase II study of a Pseudomonas vaccine in ventilated ICU patients.	**Inappropriate patient population** (mechanically ventilated ICU patients without clear evidence of sepsis or septic shock)
Ali et al. [115]	Phase 1 study of MEDI3902, an investigational anti-Pseudomonas aeruginosa PcrV and Psl bispecific human monoclonal antibody, in healthy adults.	**Inappropriate patient population** (healthy adult volunteers)
Chastre et al. [116]	COMBACTE-MAGNET EVADE Study Group. Safety, efficacy, and pharmacokinetics of gremubamab (MEDI3902), an anti-Pseudomonas aeruginosa bispecific human monoclonal antibody, in P. aeruginosa-colonised, mechanically ventilated intensive care unit patients: a randomised controlled trial.	**Inappropriate patient population** (mechanically ventilated ICU patients)
Dhainaut et al. [117]	Extended drotrecogin alfa (activated) treatment in patients with prolonged septic shock.	**Inappropriate intervention** (recombinant human activated protein C, not mAb or pAb)
Ranieri et al. [118]	Drotrecogin alfa (activated) in adults with septic shock.	**Inappropriate intervention** (Drotrecogin alfa, not mAb or pAb)
Carlone et al. [119]	Pentaglobin Efficacy in Reducing the Incidence of Sepsis and Transplant-Related Mortality in Pediatric Patients Undergoing Hematopoietic Stem Cell Transplantation: A Retrospective Study.	**Inappropriate patient population** (pediatric patients undergoing hematopoietic stem cell transplantation)
Douzinas et al. [120]	Prevention of infection in multiple trauma patients by high-dose intravenous immunoglobulins.	**Study population has different clinical indication**

		(trauma patients rather than patients with sepsis or septic shock)
Fomsgaard et al. [121]	Effect of a human IgG preparation rich in antibodies to a wide range of lipopolysaccharides on gram-negative bacterial sepsis in burned mice.	**Inappropriate setting** (animal study)
Gooding et al. [122]	Safety and efficacy of intravenous immunoglobulin prophylaxis in pediatric head trauma patients: a double-blind controlled trial.	**Inappropriate patient population** (pediatric patients with severe head trauma)
Bancalari et al. [123]	Prolonged intravenous immunoglobulin treatment in very low birth weight infants with late onset sepsis.	**Inappropriate patient population** (neonates/very low birth weight infants)

References

1. Abraham, E.; Glauser, M.P.; Butler, T.; Garbino, J.; Gelmont, D.; Laterre, P.F.; Kudsk, K.; Bruining, H.A.; Otto, C.; Tobin, E.; et al. p55 Tumor Necrosis Factor Receptor Fusion Protein in the Treatment of Patients with Severe Sepsis and Septic Shock: A Randomized Controlled Multicenter Trial. *JAMA* **1997**, 277, 1531–1538. https://doi.org/10.1001/jama.1997.03540430043031.
2. Aitchison, J.M.; Arbuckle, D.D. Anti-Endotoxin in the Treatment of Severe Surgical Septic Shock. Results of a Randomized Double-Blind Trial. *S. Afr. Med. J.* **1985**, 68, 787–789. Available online: https://journals.co.za/doi/pdf/10.10520/AJA20785135_5338 (accessed on 20 August 2025).
3. Biagioni, E.; Tosi, M.; Berlot, G.; Castiglione, G.; Corona, A.; De Cristofaro, M.G.; Donati, A.; Feltracco, P.; Forfori, F.; Fragranza, F.; et al. Adjunctive IgM-Enriched Immunoglobulin Therapy with a Personalised Dose Based on Serum IgM-Titres versus Standard Dose in the Treatment of Septic Shock: A Randomised Controlled Trial (IgM-fat Trial). *BMJ Open* **2021**, 11, e036616. https://doi.org/10.1136/bmjopen-2019-036616
4. Burns, E.R.; Lee, V.; Rubinstein, A. Treatment of Septic Thrombocytopenia with Immune Globulin. *J. Clin. Immunol.* **1991**, 11, 363–368. https://doi.org/10.1007/BF00918802
5. Calandra, T.; Glauser, M.P.; Schellekens, J.; Verhoef, J. Treatment of Gram-Negative Septic Shock with Human IgG Antibody to Escherichia coli J5: A Prospective, Double-Blind, Randomized Trial. *J. Infect. Dis.* **1988**, 158, 312–319. https://doi.org/10.1093/infdis/158.2.312
6. Clark, M.A.; Plank, L.D.; Connolly, A.B.; Streat, S.J.; Hill, A.A.; Gupta, R.; Monk, D.N.; Shenkin, A.; Hill, G.L. Effect of a Chimeric Antibody to Tumor Necrosis Factor-Alpha on Cytokine and Physiologic Responses in Patients with Severe Sepsis—A Randomized, Clinical Trial. *Crit. Care Med.* **1998**, 26, 1650–1659. https://doi.org/10.1097/00003246-199810000-00016
7. Dominioni, L.; Dionigi, R.; Zanello, M.; et al. Effects of High-Dose IgG on Survival of Surgical Patients with Sepsis Scores of 20 or Greater. *Arch. Surg.* **1991**, 126, 236–240. https://doi.org/10.1001/archsurg.1991.01410260126018
8. Dominioni, L.; Bianchi, V.; Imperatori, A.; Minoia, G.; Dionigi, R. High-Dose Intravenous IgG for Treatment of Severe Surgical Infections. *Dig. Surg.* **1996**, *13*, 430–434. https://doi.org/10.1159/000172479
9. Domizi, R.; Adrario, E.; Damiani, E.; Scorcella, C.; Carsetti, A.; Giaccaglia, P.; Casarotta, E.; Gabbanelli, V.; Pantanetti, S.; Lamura, E.; et al. IgM-Enriched Immunoglobulins (Pentaglobin) May Improve the Microcirculation in Sepsis: A Pilot Randomized Trial. *Ann. Intensive Care* **2019**, 9, 135. https://doi.org/10.1186/s13613-019-0609-5
10. François, B.; Mercier, E.; Gonzalez, C.; Asehnoune, K.; Nseir, S.; Fiancette, M.; Desachy, A.; Plantefève, G.; Meziani, F.; de Lame, P.-A.; Laterre, P.-F.; et al. Safety and Tolerability of a Single Administration of AR-301, a Human

Monoclonal Antibody, in ICU Patients with Severe Pneumonia Caused by Staphylococcus aureus: First-in-Human Trial. *Intensive Care Med.* **2018**, 44, 1787–1796. https://doi.org/10.1007/s00134-018-5229-2
11. François, B.; Jafri, H.S.; Chastre, J.; Sánchez-García, M.; Eggimann, P.; Dequin, P.-F.; Huberlant, V.; Viña Soria, L.; Boulain, T.; Bretonnière, C.; et al. Efficacy and Safety of Suvratoxumab for Prevention of Staphylococcus aureus Ventilator-Associated Pneumonia (SAATELLITE): A Multicentre, Randomised, Double-Blind, Placebo-Controlled, Parallel-Group, Phase 2 Pilot Trial. *Lancet Infect. Dis.* **2021**, 21, 1313–1323. https://doi.org/10.1016/S1473-3099(20)30995-6
12. Geven, C.; Blet, A.; Kox, M.; Hartmann, O.; Scigalla, P.; Zimmermann, J.; Marx, G.; Laterre, P.-F.; Mebazaa, A.; Pickkers, P. A Double-Blind, Placebo-Controlled, Randomised, Multicentre, Proof-of-Concept and Dose-Finding Phase II Clinical Trial to Investigate the Safety, Tolerability and Efficacy of Adrecizumab in Patients with Septic Shock and Elevated Adrenomedullin Concentration (AdrenOSS-2). *BMJ Open* **2019**, 9, e024475. https://doi.org/10.1136/bmjopen-2018-024475
13. Greenberg, R.N.; Wilson, K.M.; Kunz, A.Y.; Wedel, N.I.; Gorelick, K.J. Observations Using Antiendotoxin Antibody (E5) as Adjuvant Therapy in Humans with Suspected, Serious, Gram-Negative Sepsis. *Crit. Care Med.* **1992**, 20, 730–735. https://doi.org/10.1097/00003246-199206000-00005
14. Grundmann, R.; Hornung, M. Immunoglobulin Therapy in Patients with Endotoxemia and Postoperative Sepsis—A Prospective Randomized Study. *Prog. Clin. Biol. Res.* **1988**, 272, 339–349. Available online: https://pubmed.ncbi.nlm.nih.gov/3293080/ (accessed on 20 August 2025).
15. Hentrich, M.; Fehnle, K.; Ostermann, H.; Kienast, J.; Cornely, O.; Salat, C.; Übelacker, R.; Buchheidt, D.; Behre, G.; Hiddemann, W.; Schiel, X. IgMA-Enriched Immunoglobulin in Neutropenic Patients with Sepsis Syndrome and Septic Shock: A Randomized, Controlled, Multiple-Center Trial. *Crit. Care Med.* **2006**, 34, 1319–1325. https://doi.org/10.1097/01.CCM.0000215452.84291.C6
16. Jaspers, L.; Langecker, P.; Obermeier, A.; Kastenbauer, E.; Marget, W.; Mar, P.J.; Hoffmann, K.; Ruckdeschel, G. Antikörper gegen Lipoid A in der Behandlung des septischen Schocks. *Infection* **1987**, 15, 146–152. https://doi.org/10.1007/BF01650221
17. Just, H.M.; Metzger, M.; Vogel, W.; Pelka, R.B. Effect of Adjuvant Immunoglobulin Therapy on Infections in Patients in a Surgical Intensive Care Unit: Results of a Randomized Controlled Study. *Klin. Wochenschr.* **1986**, 64, 245–256. https://doi.org/10.1007/BF01711930
18. Kett, D.H.; Quartin, A.A.; Sprung, C.L.; Fisher, C.J., Jr.; Peña, M.A.; Heard, S.O.; Zimmerman, J.L.; Albertson, T.E.; Panacek, E.A.; Eidelman, L.A.; et al. An Evaluation of the Hemodynamic Effects of HA-1A Human Monoclonal Antibody in Patients with Presumed Gram-Negative Sepsis: A Post Hoc Analysis. *Crit. Care Med.* **1994**, 22, 1227–1234. https://doi.org/10.1097/00003246-199408000-00005
19. Lindquist, L.; Lundbergh, P.; Maasing, R. Pepsin-Treated Human Gamma Globulin in Bacterial Infections: A Randomized Study in Patients with Septicaemia and Pneumonia. *Vox Sang.* **1981**, 40, 329–337. https://doi.org/10.1111/j.1423-0410.1981.tb00717.x
20. McCarthy, M.W. Optimizing the Use of Vilobelimab for the Treatment of COVID-19. *Expert Opin. Biol. Ther.* **2023**, 23, 877–881. https://doi.org/10.1080/14712598.2023.2235269
21. Schedel, I.; Dreikhausen, U.; Nentwig, B.; Höckenschnieder, M.; Rauthmann, D.; Balikcioglu, S.; Coldewey, R.; Deicher, H. Treatment of Gram-Negative Septic Shock with an Immunoglobulin Preparation: A Prospective, Randomized Clinical Trial. *Crit. Care Med.* **1991**, 19, 1104–1113. https://doi.org/10.1097/00003246-199109000-00003
22. Tugrul, S.; Ergin Özcan, P.; Akinci, O.; Seyhun, Y.; Çağatay, A.; Çakar, N.; Esen, F. The Effects of IgM-Enriched Immunoglobulin Preparations in Patients with Severe Sepsis. *Crit. Care* **2002**, 6, 357–362. https://doi.org/10.1186/cc1523
23. Vlaar, A.P.J.; de Bruin, S.; Busch, M.; Timmermans, S.A.M.E.G.; van Zeggeren, I.E.; Koning, R.; ter Horst, L.; Bulle, E.B.; van Baarle, F.E.H.P.; van de Poll, M.C.G.; et al. Anti-C5a Antibody IFX-1 (Vilobelimab) Treatment versus Best

Supportive Care for Patients with Severe COVID-19 (PANAMO): An Exploratory, Open-Label, Phase 2 Randomised Controlled Trial. *Lancet Rheumatol.* **2020**, 2, e764–e773. https://doi.org/10.1016/S2665-9913(20)30341-6
24. Wortel, C.H.; von der Möhlen, M.A.; van Deventer, S.J.; Sprung, C.L.; Jastremski, M.; Lubbers, M.J.; Smith, C.R.; Allen, I.E.; ten Cate, J.W. Effectiveness of a Human Monoclonal Anti-Endotoxin Antibody (HA-1A) in Gram-Negative Sepsis: Relationship to Endotoxin and Cytokine Levels. *J. Infect. Dis.* **1992**, 166, 1367–1374. https://doi.org/10.1093/infdis/166.6.1367
25. Abraham, E.; Laterre, P.-F.; Garg, R.; Levy, H.; Talwar, D.; Trzaskoma, B.L.; François, B.; et al. Drotrecogin Alfa (Activated) for Adults with Severe Sepsis and a Low Risk of Death. *N. Engl. J. Med.* **2005**, *353*, 1332–1341. https://doi.org/10.1056/NEJMoa050935
26. Bone, R.C.; Fisher, C.J., Jr.; Clemmer, T.P.; Slotman, G.J.; Metz, C.A.; Balk, R.A. A Controlled Clinical Trial of High-Dose Methylprednisolone in the Treatment of Severe Sepsis and Septic Shock. *N. Engl. J. Med.* **1987**, 317, 653–658. https://doi.org/10.1056/NEJM198709103171101
27. INIS Collaborative Group. Treatment of Neonatal Sepsis with Intravenous Immune Globulin. *N. Engl. J. Med.* **2011**, 365, 1201–1211. https://doi.org/10.1056/NEJMoa1100441
28. Fisher, C.J., Jr.; Agosti, J.M.; Opal, S.M.; Lowry, S.F.; Balk, R.A.; Sadoff, J.C.; Abraham, E.; Schein, R.M.H.; Benjamin, E. Treatment of Septic Shock with the Tumor Necrosis Factor Receptor:Fc Fusion Protein. *N. Engl. J. Med.* **1996**, 334, 1697–1702. https://doi.org/10.1056/NEJM199606273342603
29. Fisher, C.J., Jr.; Slotman, G.J.; Opal, S.M.; Pribble, J.P.; Bone, R.C.; Emmanuel, G.; Ng, D.; Bloedow, D.C.; Catalano, M.A. Initial Evaluation of Human Recombinant Interleukin-1 Receptor Antagonist in the Treatment of Sepsis Syndrome: A Randomized, Open-Label, Placebo-Controlled Multicenter Trial. *Crit. Care Med.* **1994**, 22, 12–21. https://doi.org/10.1097/00003246-199401000-00008
30. Reinhart, K.; Karzai, W. Anti-Tumor Necrosis Factor Therapy in Sepsis: Update on Clinical Trials and Lessons Learned. *Crit. Care Med.* **2001**, 29, 121–125. https://doi.org/10.1097/00003246-200107001-00037
31. Fisher, C.J., Jr.; Opal, S.M.; Dhainaut, J.-F.; Stephens, S.; Zimmerman, J.L.; Nightingale, P.; Harris, S.J.; Schein, R.M.H.; Panacek, E.A.; Vincent, J.-L.; et al. Influence of an Anti-Tumor Necrosis Factor Monoclonal Antibody on Cytokine Levels in Patients with Sepsis. *Crit. Care Med.* **1993**, 21, 318–327. https://doi.org/10.1097/00003246-199303000-00006
32. Kaul, R.; McGeer, A.; Norrby-Teglund, A.; Kotb, M.; Schwartz, B.; O'Rourke, K.; Talbot, J.; Low, D.E. Intravenous Immunoglobulin Therapy for Streptococcal Toxic Shock Syndrome—A Comparative Observational Study. *Clin. Infect. Dis.* **1999**, 28, 800–807. https://doi.org/10.1086/515199
33. Leeies, M.; Gershengorn, H.B.; Charbonney, E.; Kumar, A.; Fergusson, D.; Turgeon, A.F.; Cowan, J.; Paunovic, B.; Embil, J.; Houston, D.S.; et al. Intravenous Immune Globulin in Septic Shock: A Canadian National Survey of Critical Care Medicine and Infectious Disease Specialist Physicians. *Can. J. Anaesth.* **2021**, 68, 782–790. https://doi.org/10.1007/s12630-021-01941-3
34. Laupland, K.B. Polyclonal Intravenous Immunoglobulin for the Prophylaxis and Treatment of Infection in Critically Ill Adults. *Can. J. Infect. Dis.* **2002**, 13, 100–106. https://doi.org/10.1155/2002/127953
35. Hung, I.F.N.; To, K.K.W.; Lee, C.K.; Lee, K.L.; Yan, W.W.; Chan, K.; Chan, W.M.; Ngai, C.W.; Law, K.I.; Chow, F.L.; et al. Hyperimmune IV Immunoglobulin Treatment: A Multicenter Double-Blind Randomized Controlled Trial for Patients with Severe 2009 Influenza A(H1N1) Infection. *Chest* **2013**, 144, 464–473. https://doi.org/10.1378/chest.12-2907
36. Kalvelage, C.; Marx, G.; Eckmann, C.; Pletz, M.W.; Bracht, H.; Simon, T.-P.; Winkler, M.; Kindgen-Milles, D.; Albertsmeier, M.; Weigand, M.; et al. Personalized Medicine with IgGAM Compared with Standard of Care for Treatment of Peritonitis after Infectious Source Control (the PEPPER Trial): Study Protocol for a Randomized Controlled Trial. *Trials* **2019**, 20, 156. https://doi.org/10.1186/s13063-019-3244-4
37. Levin, M.; Quint, P.A.; Goldstein, B.; Barton, P.; Bradley, J.S.; Shemie, S.D.; Yeh, T.; Kim, S.S.; Cafaro, D.P.; Scannon, P.J.; Giroir, B.P. Recombinant Bactericidal/Permeability-Increasing Protein (rBPI21) as Adjunctive Treatment for Children with Severe Meningococcal Sepsis: A Randomised Trial. *Lancet* **2000**, 356, 961–967. https://doi.org/10.1016/S0140-6736(00)02712-4

38. Ohlsson, A.; Lacy, J.B. Intravenous Immunoglobulin for Preventing Infection in Preterm and/or Low Birth Weight Infants. *Cochrane Database Syst. Rev.* **2020**, CD000361. https://doi.org/10.1002/14651858.CD000361.pub4
39. Migone, T.S.; Subramanian, G.M.; Zhong, J.; Healey, L.M.; Corey, A.; Devalaraja, M.; Lo, L.; Ullrich, S.; Zimmerman, J.; Chen, A.; et al. Raxibacumab for the Treatment of Inhalational Anthrax. *N. Engl. J. Med.* **2009**, 361, 135–144. https://doi.org/10.1056/NEJMoa0810603
40. Tocut, M.; Kolitz, T.; Shovman, O.; Haviv, Y.; Boaz, M.; Laviel, S.; Debi, S.; Nama, M.; Akria, A.; Shoenfeld, Y.; et al. Outcomes of ICU Patients Treated with Intravenous Immunoglobulin for Sepsis or Autoimmune Diseases. *Autoimmun. Rev.* **2022**, 21, 103205. https://doi.org/10.1016/j.autrev.2022.103205
41. Scarpati, G.; Baldassarre, D.; Tripepi, G.; Boffardi, M.; Piazza, O. Effect of Intravenous IgM-Enriched Immunoglobulins on Presepsin and Other Sepsis Biomarkers. *Front. Pharmacol.* **2021**, 12, 717349. https://doi.org/10.3389/fphar.2021.717349
42. Derkx, B.; Wittes, J.; McCloskey, R. Randomized, Placebo-Controlled Trial of HA-1A, a Human Monoclonal Antibody to Endotoxin, in Children with Meningococcal Septic Shock. *Clin. Infect. Dis.* **1999**, 28, 770–777. https://doi.org/10.1086/515184
43. Lee, W.Y.; Kim, H.J.; Kim, E.Y. Impact of Polymyxin B Hemoperfusion Therapy on High Endotoxin Activity Level Patients After Successful Infection Source Control: A Prospective Cohort Study. *Sci. Rep.* **2021**, 11, 24132. https://doi.org/10.1038/s41598-021-03055-8
44. Senda, A.; Endo, A.; Fushimi, K.; Otomo, Y. Effectiveness of Intravenous Immunoglobulin Therapy for Invasive Group A Streptococcus Infection: A Japanese Nationwide Observational Study. *Int. J. Infect. Dis.* **2023**, 135, 84–90. https://doi.org/10.1016/j.ijid.2023.08.011
45. Akdag, A.; Dilmen, U.; Haque, K.; Dilli, D.; Erdeve, O.; Goekmen, T. Role of Pentoxifylline and/or IgM-Enriched Intravenous Immunoglobulin in the Management of Neonatal Sepsis. *Am. J. Perinatol.* **2014**, 31, 905–912. https://doi.org/10.1055/s-0033-1363771
46. Elgendy, H.A.; Ibrahim, H.M.; Hasan, B.E.; Elkawe, A.S.A. Effect of Pentoxifylline on Organ Dysfunction and Mortality in Severe Sepsis. *Open Anesthesiol. J.* **2020**, 14, 14–21. https://doi.org/10.2174/2589645802014010014
47. Aggarwal, R.; Dewan, A.; Pandey, A.; Soni, R.; Singh, S.; Kaur, S.; Kumar, A.; Soni, S.; Bansal, R.; Gupta, S. Efficacy of High-Dose Intravenous Immunoglobulin in Severe and Critical COVID-19: A Retrospective Cohort Study. *Int. Immunopharmacol.* **2022**, 106, 108615. https://doi.org/10.1016/j.intimp.2022.108615
48. Klein, D.J.; Foster, D.; Walker, P.M.; Bagshaw, S.M.; Mekonnen, H.; Antonelli, M. Polymyxin B Hemoperfusion in Endotoxemic Septic Shock Patients Without Extreme Endotoxemia: A Post Hoc Analysis of the EUPHRATES Trial. *Intensive Care Med.* **2018**, 44, 2205–2212. https://doi.org/10.1007/s00134-018-5463-7
49. Choi, Y.U.; Kim, J.G.; Jang, J.Y.; Go, T.H.; Kim, K.; Bae, K.S.; Shim, H. Adjuvant Intravenous Immunoglobulin Administration in Postoperative Critically Ill Patients with Secondary Peritonitis: A Retrospective Study. *Acute Crit. Care* **2023**, 38, 21–30. https://doi.org/10.4266/acc.2022.01515
50. Moughames, E.; Sakayan, S.; Prichett, L.; Runken, M.C.; Borst, D.; Tversky, J.; Azar, A. Outcomes of Intravenous Immunoglobulin Treatment of Immunocompromised Patients with Viral Respiratory Infections. *Ann. Allergy Asthma Immunol.* **2025**, *134*, 85–90.e1. https://doi.org/10.1016/j.anai.2024.09.001
51. Derhaschnig, U.; Reiter, R.; Knöbl, P.; Baumgartner, M.; Keen, P.; Jilma, B. Recombinant Human Activated Protein C (rhAPC; Drotrecogin Alfa [Activated]) Has Minimal Effect on Markers of Coagulation, Fibrinolysis, and Inflammation in Acute Human Endotoxemia. *Blood* **2003**, *102*, 2093–2098. https://doi.org/10.1182/blood-2003-02-0416
52. Portefaix, A.; Dhelens, C.; Recher, M.; Cour-Andlauer, F.; Naudin, J.; Mortamet, G.; Joram, N.; Tissières, P.; Ginhoux, T.; Kassai, B.; et al. High-Dose Intravenous Immunoglobulin versus Albumin 4% in Paediatric Toxic Shock Syndrome: A Randomised Controlled Feasibility Study. *Arch. Dis. Child.* **2024**, *109*, 717–723. https://doi.org/10.1136/archdischild-2022-325274

53. Presneill, J.J.; Harris, T.; Stewart, A.G.; Cade, J.F.; Wilson, J.W. A Randomized Phase II Trial of Granulocyte-Macrophage Colony-Stimulating Factor Therapy in Severe Sepsis with Respiratory Dysfunction. *Am. J. Respir. Crit. Care Med.* **2002**, *166*, 138–143. https://doi.org/10.1164/rccm.2009005
54. Cometta, A.; Baumgartner, J.-D.; Lee, M.L.; Hanique, G.; Glauser, M.-P. Prophylactic Intravenous Administration of Standard Immune Globulin as Compared with Core-Lipopolysaccharide Immune Globulin in Patients at High Risk of Postsurgical Infection. *N. Engl. J. Med.* **1992**, *327*, 234–240. https://doi.org/10.1056/NEJM199207233270404
55. McCulloch, L.; Harris, A.J.; Malbon, A.; Daniels, M.J.D.; Younas, M.; Grainger, J.R.; Allan, S.M.; Smith, C.J.; McColl, B.W. Treatment with IgM-Enriched Intravenous Immunoglobulins Enhances Clearance of Stroke-Associated Bacterial Lung Infection. *Immunology* **2022**, *167*, 558–575. https://doi.org/10.1111/imm.13553
56. Welte, T.; Dellinger, R.P.; Ebelt, H.; Werdan, K.; Löffler, K.; Torres, A. Concept for a Study Design in Patients with Severe Community-Acquired Pneumonia: A Randomised Controlled Trial with a Novel IgM-Enriched Immunoglobulin Preparation – the CIGMA Study. *Respir. Med.* **2015**, *109*, 758–767. https://doi.org/10.1016/j.rmed.2015.03.008
57. Singer, M.; Torres, A.; Heinz, C.C.; Weißmüller, S.; Staus, A.; Kistner, S.; Jakubczyk, K.; Häder, T.; Langohr, P.; Wartenberg-Demand, A.; Schüttrumpf, J.; Vincent, J.-L.; Welte, T. The Immunomodulating Activity of Trimodulin (Polyvalent IgM, IgA, IgG Solution): A Post Hoc Analysis of the Phase II CIGMA Trial. *Crit. Care* **2023**, *27*, 436. https://doi.org/10.1186/s13054-023-04719-9
58. Farkhutdinov, U. Intravenous Immunoglobulin in Community Acquired Pneumonia. *Eur. Respir. J.* **2011**, *38*, 818. Online Available: https://publications.ersnet.org/content/erj/38/suppl55/p818 (accessed on 20 August 2025).
59. Rupp, M.E.; Holley, H.P. Jr.; Lutz, J.; Dicpinigaitis, P.V.; Woods, C.W.; Levine, D.P.; Veney, N.; Fowler, V.G. Jr. Phase II, Randomized, Multicenter, Double-Blind, Placebo-Controlled Trial of a Polyclonal Anti-Staphylococcus aureus Capsular Polysaccharide Immune Globulin in Treatment of Staphylococcus aureus Bacteremia. *Antimicrob. Agents Chemother.* **2007**, *51*, 4249–4254. https://doi.org/10.1128/AAC.00570-07
60. Agafina, A.; Aguiar, V.C.; Rossovskaya, M.; Fartoukh, M.S.; Hajjar, L.A.; Thiéry, G.; Timsit, J.-F.; Gordeev, I.; Protsenko, D.; Carbone, J.; et al. Efficacy and Safety of Trimodulin in Patients with Severe COVID-19: Results from a Randomised, Placebo-Controlled, Double-Blind, Multicentre, Phase II Trial (ESsCOVID). *Eur. J. Med. Res.* **2024**, 29, 418. https://doi.org/10.1186/s40001-024-02008-x
61. Newham, P.; Ross, D.; Ceuppens, P.; Das, S.; Yates, J.W.T.; Betts, C.; Reens, J.; Randall, K.J.; Knight, R.; McKay, J.S. Determination of the Safety and Efficacy of Therapeutic Neutralization of Tumor Necrosis Factor-α (TNF-α) Using AZD9773, an Anti-TNF-α Immune Fab, in Murine CLP Sepsis. *Inflamm. Res.* **2014**, *63*, 149–160. https://doi.org/10.1007/s00011-013-0683-3
62. Newham, P.; Ceuppens, P.; Das, S.; Yates, J.W.T.; Knight, R.; McKay, J.S. AZD9773, a novel anti-TNFα immune Fab in development for severe sepsis and septic shock: demonstration of safety and efficacy in a murine CLP sepsis model. *Crit. Care* **2011**, *15*, P41. https://doi.org/10.1186/cc10410
63. Chen, G.; Li, N.; Dai, X.; Tu, S.; Shen, Z.; Wu, K.; Jin, T.; Wu, J.; Peng, C.; Sheng, G.; et al. Safety, Tolerability, Pharmacokinetics, and Pharmacodynamics of Anti-C5a Antibody BDB-001 for Severe COVID-19: A Randomized, Double-Blind, Placebo-Controlled Phase 1 Clinical Trial in Healthy Chinese Adults. *Infect. Dis. Ther.* **2023**, *12*, 1059–1072. https://doi.org/10.1007/s40121-023-00759-4
64. Rizvi, M.Q.; Singh, M.V.; Mishra, N.; Shrivastava, A.; Maurya, M.; Siddiqui, S.A. Intravenous immunoglobulin in the management of neonatal sepsis: A randomised controlled trial. *Trop. Doct.* **2023**, *53*, 222–226. https://doi.org/10.1177/00494755221138689
65. Shenoi, A.; Nagesh, N.K.; Maiya, P.P.; Bhat, S.R.; Subba Rao, S.D. Multicenter Randomized Placebo-Controlled Trial of Therapy with Intravenous Immunoglobulin in Decreasing Mortality Due to Neonatal Sepsis. *Indian Pediatr.* **1999**, *36*, 1113–1118. Online Available: https://pubmed.ncbi.nlm.nih.gov/10745332/ (accessed on 20 August 2025).

66. Horspool, A.M.; Sen-Kilic, E.; Malkowski, A.C.; Breslow, S.L.; Mateu-Borras, M.; Hudson, M.S.; Nunley, M.A.; Elliott, S.; Ray, K.; Snyder, G.A.; et al. Development of an Anti-Pseudomonas aeruginosa Therapeutic Monoclonal Antibody WVDC-5244. *Front. Cell. Infect. Microbiol.* **2023**, *13*, 1117844. https://doi.org/10.3389/fcimb.2023.1117844
67. Sandberg, K.; Fasth, A.; Berger, A.; Eibl, M.; Isacson, K.; Lischka, A.; Pollak, A.; Tessin, I.; Thiringer, K. Preterm Infants with Low Immunoglobulin G Levels Have Increased Risk of Neonatal Sepsis but Do Not Benefit from Prophylactic Immunoglobulin G. *J. Pediatr.* **2000**, *137*, 623–628. https://doi.org/10.1067/mpd.2000.109791
68. Fang, K.C. Monoclonal Antibodies to Endotoxin in the Management of Sepsis. *West. J. Med.* **1993**, *158*, 393–399. Online Available: https://pmc.ncbi.nlm.nih.gov/articles/PMC1022067/ (accessed on 20 August 2025).
69. Cross, A.S.; Opal, S.M.; Palardy, J.E.; Shridhar, S.; Baliban, S.M.; Scott, A.J.; Chahin, A.B.; Ernst, R.K. A Pilot Study of an Anti-Endotoxin Ig-Enriched Bovine Colostrum to Prevent Experimental Sepsis. *Innate Immun.* **2021**, *27*, 266–274. https://doi.org/10.1177/17534259211007538
70. Soares, M.O.; Welton, N.J.; Harrison, D.A.; Peura, P.; Shankar-Hari, M.; Harvey, S.E.; Madan, J.J.; Ades, A.E.; Palmer, S.J.; Rowan, K.M. An Evaluation of the Feasibility, Cost and Value of Information of a Multicentre Randomised Controlled Trial of Intravenous Immunoglobulin for Sepsis (Severe Sepsis and Septic Shock): Incorporating a Systematic Review, Meta-Analysis and Value of Information Analysis. *Health Technol. Assess.* **2012**, *16*, 1–186. https://doi.org/10.3310/hta16070
71. Soares, M.O.; Welton, N.J.; Harrison, D.A.; Peura, P.; Shankar-Hari, M.; Harvey, S.E.; Madan, J.; Ades, A.E.; Rowan, K.M.; Palmer, S.J. Intravenous Immunoglobulin for Severe Sepsis and Septic Shock: Clinical Effectiveness, Cost-Effectiveness and Value of a Further Randomised Controlled Trial. *Crit. Care* **2014**, *18*, 649. https://doi.org/10.1186/s13054-014-0649-z
72. Akatsuka, M.; Masuda, Y.; Tatsumi, H.; Sonoda, T. Efficacy of Intravenous Immunoglobulin Therapy for Patients With Sepsis and Low Immunoglobulin G Levels: A Single-Center Retrospective Study. *Clin. Ther.* **2022**, *44*, 295–303. https://doi.org/10.1016/j.clinthera.2021.12.008
73. Ando, Y.; Inoue, S.; Kawashima, T.; Okashiro, M.; Kotani, J.; Nishiyama, T. Intravenous Immunoglobulin G Modulates the Expression of Sepsis-Induced Coagulopathy Factors and Increases Serum IgM Levels: A Prospective, Single-Center Intervention Study. *Kobe J. Med. Sci.* **2020**, *66*, E32–E39. Online Available: https://pmc.ncbi.nlm.nih.gov/articles/PMC7447101/ (accessed on 20 August 2025).
74. Goto, K.; Yasuda, N.; Sato, Y. Effects of Low-Dose Intravenous Immunoglobulin as Adjunctive Therapy in Septic Shock Patients with and without Hypogammaglobulinemia: A Retrospective Cohort Study. *Ann. Palliat. Med.* **2022**, *11*, 2600–2608. https://doi.org/10.21037/apm-21-3694
75. Stephens, D.P.; Fisher, D.A.; Currie, B.J. Randomized, Double-Blind, Placebo-Controlled Trial of Granulocyte Colony-Stimulating Factor in Patients with Septic Shock. *Crit. Care Med.* **2008**, *36*, 448–454. https://doi.org/10.1097/01.CCM.0B013E318161E480
76. Behre, G.; Schedel, I.; Nentwig, B.; Wörmann, B.; Essink, M.; Hiddemann, W. Endotoxin Concentration in Neutropenic Patients with Suspected Gram-Negative Sepsis: Correlation with Clinical Outcome and Determination of Anti-Endotoxin Core Antibodies during Therapy with Polyclonal Immunoglobulin M-Enriched Immunoglobulins. *Antimicrob. Agents Chemother.* **1992**, *36*, 2139–2146. https://doi.org/10.1128/aac.36.10.2139
77. Buda, S.; Riefolo, A.; Biscione, R.; Goretti, E.; Cattabriga, I.; Grillone, G.; Bacchi-Reggiani, L.; Pacini, D. Clinical Experience with Polyclonal IgM-Enriched Immunoglobulins in a Group of Patients Affected by Sepsis after Cardiac Surgery. *J. Cardiothorac. Vasc. Anesth.* **2005**, *19*, 440–445. https://doi.org/10.1053/j.jvca.2005.05.003
78. Sakka, S.G. Sepsis bei einer Patientin mit Autoimmunerkrankung und immunsuppressiver Therapie – Einsatz eines polyvalenten IgGAM-Präparates. *Anästh. Intensivmed.* **2021**, *62*, 82. https://doi.org/10.19224/ai2021.082
79. Vogel, F. Bewertung der intravenösen IgM-Therapie bei schweren nosokomialen Infektionen (Ergebnis einer kontrollierten randomisierten Studie). In: Deicher H, Schoeppe W, Herausgeber. Klinisch angewandte Immunologie. Springer, Berlin, Heidelberg; **1988**. S. 30–41. https://doi.org/10.1007/978-3-642-73415-1_3

80. Yavuz, L.; Aynali, G.; Aynali, A.; Alaca, A.; Kutuk, S.; Ceylan, B.G. The Effects of Adjuvant Immunoglobulin M-Enriched Immunoglobulin Therapy on Mortality Rate and Renal Function in Sepsis-Induced Multiple Organ Dysfunction Syndrome: Retrospective Analysis of Intensive Care Unit Patients. *J. Int. Med. Res.* **2012**, *40*, 1166–1174. https://doi.org/10.1177/147323001204000337
81. Kakoullis, L.; Velissaris, D.; Tsagkaris, C.; Tzannis, K.; Papadopoulos, A.; Papadopoulou, D.; Papadopoulou, A.; Tsakris, A.; Dimopoulos, G.; Falagas, M.E. The Use of IgM-Enriched Immunoglobulin in Adult Patients with Sepsis. *J. Crit. Care* **2018**, *47*, 1–8. https://doi.org/10.1016/j.jcrc.2018.06.005
82. Masaoka, T. Effects of Intravenous Immunoglobulin Therapy in Patients with Severe Sepsis. *Nihon Rinsho (Jpn. J. Clin. Med.)* **2001**, *59*, 781–784. Online Available: https://pubmed.ncbi.nlm.nih.gov/11305006/ (accessed on 20 August 2025).
83. Opal, S.M.; Fisher, C.J.; Dhainaut, J.F.; Vincent, J.L.; Brase, R.; Lowry, S.F.; Sadoff, J.C.; Slotman, G.J.; Levy, H.; Balk, R.A.; et al. Confirmatory Interleukin-1 Receptor Antagonist Trial in Severe Sepsis: A Phase III, Randomized, Double-Blind, Placebo-Controlled, Multicenter Trial. *Crit. Care Med.* **1997**, *25*, 1115–1124. https://doi.org/10.1097/00003246-199707000-00010
84. Dhainaut, J.F.A.; Tenaillon, A.; Le Tulzo, Y.; Schlemmer, B.; Solet, J.P.; Wolff, M.; Holzapfel, L.; Zeni, F.; Dreyfuss, D.; Mira, J.P.; et al. Platelet-Activating Factor Receptor Antagonist BN 52021 in the Treatment of Severe Sepsis: A Randomized, Double-Blind, Placebo-Controlled, Multicenter Clinical Trial. *Crit. Care Med.* **1994**, *22*, 1720–1728. Online Available: https://pubmed.ncbi.nlm.nih.gov/7956274/ (accessed on 20 August 2025).
85. Dries, D.J.; Jurkovich, G.J.; Maier, R.V.; Clemmer, T.P.; Struve, S.N.; Weigelt, J.A.; Stanford, G.G.; Herr, D.L.; Champion, H.R.; Lewis, F.R.; et al. Effect of Interferon Gamma on Infection-Related Death in Patients with Severe Injuries: A Randomized, Double-Blind, Placebo-Controlled Trial. *Arch. Surg.* **1994**, *129*, 1031–1041. https://doi.org/10.1001/archsurg.1994.01420340045008
86. Sallam, M.M.; Abou-Aisha, K.; El-Azizi, M. A Novel Combination Approach of Human Polyclonal IVIG and Antibiotics Against Multidrug-Resistant Gram-Positive Bacteria. *Infect. Drug Resist.* **2016**, *9*, 301–311. https://doi.org/10.2147/IDR.S120227
87. Stuttmann, R.; Hartert, M.; Petrovici, V. Prophylactic Pseudomonas Immunoglobulin in Burn Patients. *Infection* **1987**, *15*, 80–84. https://doi.org/10.1007/BF01646129
88. Hansbrough, J.F.; Miller, L.M.; Field, T.O. Jr.; Gadd, M.A. High-Dose Intravenous Immunoglobulin Therapy in Burn Patients: Pharmacokinetics and Effects on Microbial Opsonization and Phagocytosis. *Pediatr. Infect. Dis. J.* **1988**, *7*, S57. Online Available: https://pubmed.ncbi.nlm.nih.gov/3041358/ (accessed on 20 August 2025).
89. Pilz, G.; Kääb, S.; Werdan, K.; Neeser, G.; Class, I.; Schweigart, U.; Brähler, A.; Bujdoso, O.; Neumann, R. Supplemental Immunoglobulin (ivIgG) Treatment in 163 Patients with Sepsis and Septic Shock—An Observational Study as a Prerequisite for Placebo-Controlled Clinical Trials. *Infection* **1991**, *19*, 216–227. https://doi.org/10.1007/BF01644948
90. Cavazzuti, I.; Serafini, G.; Busani, S.; Rinaldi, L.; Biagioni, E.; Buoncristiano, M.; Girardis, M. Early Therapy with IgM-Enriched Polyclonal Immunoglobulin in Patients with Septic Shock. *Intensive Care Med.* **2014**, *40*, 1888–1896. https://doi.org/10.1007/s00134-014-3474-6
91. Hamano, N.; Nishi, K.; Onose, A.; Okamoto, A.; Umegaki, T.; Yamazaki, E.; Hirota, K.; Ookura, H.; Takahashi, H.; Shingu, K. Efficacy of Single-Dose Intravenous Immunoglobulin Administration for Severe Sepsis and Septic Shock. *J. Intensive Care* **2013**, *1*, 4. https://doi.org/10.1186/2052-0492-1-4
92. Tagami, T.; Matsui, H.; Fushimi, K.; Yasunaga, H. Intravenous Immunoglobulin Use in Septic Shock Patients after Emergency Laparotomy. *J. Infect.* **2015**, *71*, 158–166. https://doi.org/10.1016/j.jinf.2015.04.003
93. Tagami, T.; Matsui, H.; Fushimi, K.; Yasunaga, H. Intravenous Immunoglobulin and Mortality in Pneumonia Patients with Septic Shock: An Observational Nationwide Study. *Clin. Infect. Dis.* **2015**, *61*, 385–392. https://doi.org/10.1093/cid/civ307

94. Sakoulas, G.; Geriak, M.; Kullar, R.; Greenwood, K.L.; Habib, M.; Vyas, A.; et al. Intravenous Immunoglobulin (IVIG) Significantly Reduces Respiratory Morbidity in COVID-19 Pneumonia: A Prospective Randomized Trial. *medRxiv* **2020**, 20157891. https://doi.org/10.1101/2020.07.20.20157891
95. Hellerud, B.C.; Orrem, H.L.; Dybwik, K.; Pischke, S.E.; Baratt-Due, A.; Castellheim, A.; Fure, H.; Bergseth, G.; Christiansen, D.; Nunn, M.A.; et al. Combined Inhibition of C5 and CD14 Efficiently Attenuated the Inflammatory Response in a Porcine Model of Meningococcal Sepsis. *J. Intensive Care* **2017**, *5*, 21. https://doi.org/10.1186/s40560-017-0217-0
96. Stevens, N.E.; Chapman, M.J.; Fraser, C.K.; Kuchel, T.R.; Hayball, J.D.; Diener, K.R. Therapeutic Targeting of HMGB1 during Experimental Sepsis Modulates the Inflammatory Cytokine Profile to One Associated with Improved Clinical Outcomes. *Sci. Rep.* **2017**, *7*, 5850. https://doi.org/10.1038/s41598-017-06205-z
97. Wang, H.; Zhu, S.; Zhou, R.; Li, W.; Sama, A.E. Therapeutic Potential of HMGB1-Targeting Agents in Sepsis. *Expert Rev. Mol. Med.* **2008**, *10*, e32. https://doi.org/10.1017/S1462399408000884
98. Warren, H.S.; Amato, S.F.; Fitting, C.; Black, K.M.; Loiselle, P.M.; Pasternack, M.S.; Cavaillon, J.M. Assessment of Ability of Murine and Human Anti-Lipid A Monoclonal Antibodies to Bind and Neutralize Lipopolysaccharide. *J. Exp. Med.* **1993**, *177*, 89–97. https://doi.org/10.1084/jem.177.1.89
99. Waymack, J.P.; Jenkins, M.E.; Alexander, J.W.; Warden, G.D.; Miller, A.C.; Carey, M.; Ogle, C.K.; Kopcha, R.G. A Prospective Trial of Prophylactic Intravenous Immune Globulin for the Prevention of Infections in Severely Burned Patients. *Burns* **1989**, *15*, 71–76. https://doi.org/10.1016/0305-4179(89)90132-0
100. Pedraza-Sánchez, S.; Cruz-González, A.; Palmeros-Rojas, O.; Gálvez-Romero, J.L.; Bellanti, J.A.; Torres, M. Polyvalent Human Immunoglobulin for Infectious Diseases: Potential to Circumvent Antimicrobial Resistance. *Front. Immunol.* **2022**, *13*, 987231. https://doi.org/10.3389/fimmu.2022.987231
101. Weisman, L.E.; Thackray, H.M.; Garcia-Prats, J.A.; Nesin, M.; Schneider, J.H.; Fretz, J.; Kokai-Kun, J.F.; Mond, J.J.; Kramer, W.G.; Fischer, G.W. Phase 1/2, Double-Blind, Placebo-Controlled, Dose-Escalation Study of Pagibaximab (BSYX-A110), an Antistaphylococcal Monoclonal Antibody for the Prevention of Staphylococcal Bloodstream Infections in Very-Low-Birth-Weight Neonates. *Antimicrob. Agents Chemother.* **2009**, *53*, 2879–2886. https://doi.org/10.1128/AAC.01565-08
102. Weisman, L.E.; Thackray, H.M.; Steinhorn, R.H.; Walsh, W.F.; Lassiter, H.A.; Dhanireddy, R.; Brozanski, B.S.; Palmer, K.G.; Trautman, M.S.; Escobedo, M.; et al. A Randomized Study of a Monoclonal Antibody (Pagibaximab) to Prevent Staphylococcal Sepsis. *Pediatrics* **2011**, *128*, 271–279. https://doi.org/10.1542/peds.2010-3081
103. Ishikura, H.; Nakamura, Y.; Kawano, Y.; Tanaka, J.; Mizunuma, M.; Ohta, D.; Nishida, T.; Murai, A. Intravenous Immunoglobulin Improves Sepsis-Induced Coagulopathy: A Retrospective, Single-Center Observational Study. *J. Crit. Care* **2015**, *30*, 579–583. https://doi.org/10.1016/j.jcrc.2015.02.012
104. Perrella, A.; Rinaldi, L.; Guarino, I.; Bernardi, F.F.; Castriconi, M.; Antropoli, C.; Pafundi, P.C.; Di Micco, P.; Sarno, M.; Capoluongo, N.; et al. Sepsis Outcome after Major Abdominal Surgery Does Not Seem to Be Improved by the Use of Pentameric Immunoglobulin IgM: A Single-Center Retrospective Analysis. *J. Clin. Med.* **2023**, *12*, 6887. https://doi.org/10.3390/jcm12216887
105. Dimaano, E.M.; Saito, M.; Honda, S.; Miranda, E.A.; Alonzo, M.T.; Valerio, M.D.; et al. Lack of Efficacy of High-Dose Intravenous Immunoglobulin Treatment of Severe Thrombocytopenia in Patients with Secondary Dengue Virus Infection. *Am. J. Trop. Med. Hyg.* **2007**, *77*, 1135–1138. https://doi.org/10.4269/ajtmh.2007.77.1135
106. Takahashi, G.; Shibata, S. Study of Usefulness of Low-Dose IgG for Patients with Septic Disseminated Intravascular Coagulation. *Biomark. Med.* **2020**, *14*, 1189–1196. https://doi.org/10.2217/bmm-2020-0204
107. Takano, H.; Kanda, N.; Wakimoto, Y.; Ohbe, H.; Nakamura, K. Intravenous Immunoglobulin for Mortality and Inflammatory Status in Patients with Sepsis: A Retrospective Database Study. *Front. Immunol.* **2024**, *15*, 1511481. https://doi.org/10.3389/fimmu.2024.1511481

108. Baker, C.J.; Melish, M.E.; Hall, R.T.; Casto, D.T.; Vasan, U.; Givner, L.B.; et al. Intravenous Immune Globulin for the Prevention of Nosocomial Infection in Low-Birth-Weight Neonates. *N. Engl. J. Med.* **1992**, *327*, 213–219. https://doi.org/10.1056/NEJM199207233270401
109. Fanaroff, A.A.; Korones, S.B.; Wright, L.L.; Wright, E.C.; Poland, R.L.; Bauer, C.B.; Tyson, J.E.; et al. A Controlled Trial of Intravenous Immune Globulin to Reduce Nosocomial Infections in Very-Low-Birth-Weight Infants. *N. Engl. J. Med.* **1994**, *330*, 1107–1113. https://doi.org/10.1056/NEJM199404213301602
110. Bernard, G.R.; Vincent, J.-L.; Laterre, P.-F.; LaRosa, S.P.; Dhainaut, J.-F.; Lopez-Rodriguez, A.; Steingrub, J.S.; Garber, G.E.; Helterbrand, J.D.; Ely, E.W.; et al. Efficacy and Safety of Recombinant Human Activated Protein C for Severe Sepsis. *N. Engl. J. Med.* **2001**, *344*, 699–709. https://doi.org/10.1056/NEJM200103083441001
111. Savva, A.; Roger, T. Targeting Toll-Like Receptors: Promising Therapeutic Strategies for the Management of Sepsis-Associated Pathology and Infectious Diseases. *Front. Immunol.* **2013**, *4*, 387. https://doi.org/10.3389/fimmu.2013.00387
112. Palliyil, S.; Mawer, M.; Alwafi, S.; Fogg, L.; Buda de Cesare, G.; Tan, T.; Walker, L.; MacCallum, D.; Porter, A.; Munro, C. Monoclonal Antibodies Targeting Surface-Exposed Epitopes of Candida albicans Cell Wall Proteins Confer In Vivo Protection in an Infection Model. *Antimicrob. Agents Chemother.* **2022**, 66, e01957-21. https://doi.org/10.1128/aac.01957-21
113. Rosario-Colon, J.; Eberle, K.; Xin, H. Monoclonal Antibodies Targeting Candida Disrupt Biofilms and Inhibit Growth across Global Clinical Isolates. *iScience* **2025**, 28, 112459. https://doi.org/10.1016/j.isci.2025.112459
114. Rello, J.; Krenn, C.G.; Locker, G.; Pilger, E.; Madl, C.; Balica, L.; Dugernier, T.; Laterre, P.F.; Spapen, H.; Depuydt, P.; et al. A Randomized Placebo-Controlled Phase II Study of a Pseudomonas Vaccine in Ventilated ICU Patients. *Crit. Care* **2017**, 21, 22. https://doi.org/10.1186/s13054-017-1601-9
115. Ali, S.O.; Yu, X.Q.; Robbie, G.J.; Wu, Y.; Shoemaker, K.; Yu, L.; DiGiandomenico, A.; Keller, A.E.; Anude, C.; Hernandez-Illas, M.; et al. Phase 1 Study of MEDI3902, an Investigational Anti–Pseudomonas aeruginosa PcrV and Psl Bispecific Human Monoclonal Antibody, in Healthy Adults. *Clin. Microbiol. Infect*. **2019**, 25, 629.e1–629.e6. https://doi.org/10.1016/j.cmi.2018.08.004
116. Chastre, J.; François, B.; Bourgeois, M.; Komnos, A.; Ferrer, R.; Rahav, G.; De Schryver, N.; Lepape, A.; Koksal, I.; Luyt, C.-E.; et al. Safety, Efficacy, and Pharmacokinetics of Gremubamab (MEDI3902), an Anti-*Pseudomonas aeruginosa* Bispecific Human Monoclonal Antibody, in *P. aeruginosa*-Colonised, Mechanically Ventilated Intensive Care Unit Patients: A Randomised Controlled Trial. *Crit. Care* **2022**, *26*, 355. https://doi.org/10.1186/s13054-022-04204-9
117. Dhainaut, J.-F.; Antonelli, M.; Wright, P.; Desachy, A.; Reignier, J.; Lavoué, S.; Charpentier, J.; Belger, M.; Cobas-Meyer, M.; Maier, C.; et al. Extended Drotrecogin Alfa (Activated) Treatment in Patients with Prolonged Septic Shock. *Intensive Care Med.* **2009**, 35, 1187–1195. https://doi.org/10.1007/s00134-009-1436-1
118. Ranieri, V.M.; Thompson, B.T.; Barie, P.S.; Dhainaut, J.-F.; Douglas, I.S.; Finfer, S.; Gårdlund, B.; Marshall, J.C.; Rhodes, A.; Artigas, A.; et al. Drotrecogin Alfa (Activated) in Adults with Septic Shock. *N. Engl. J. Med.* **2012**, 366, 2055–2064. https://doi.org/10.1056/NEJMoa1202290
119. Carlone, G.; Torelli, L.; Maestro, A.; Zanon, D.; Barbi, E.; Maximova, N. Pentaglobin Efficacy in Reducing the Incidence of Sepsis and Transplant-Related Mortality in Pediatric Patients Undergoing Hematopoietic Stem Cell Transplantation: A Retrospective Study. *J. Clin. Med.* **2020**, 9, 1592. https://doi.org/10.3390/jcm9051592
120. Douzinas, E.E.; Pitaridis, M.T.; Louris, G.; Andrianakis, I.; Katsouyanni, K.; Karmpaliotis, D.; Economidou, J.; Sfyras, D.; Roussos, C. Prevention of Infection in Multiple Trauma Patients by High-Dose Intravenous Immunoglobulins. *Crit. Care Med.* **2000**, *28*, 8–15. https://doi.org/10.1097/00003246-200001000-00002
121. Fomsgaard, A.; Holder, I.A. Effect of a Human IgG Preparation Rich in Antibodies to a Wide Range of Lipopolysaccharides on Gram-Negative Bacterial Sepsis in Burned Mice. *APMIS* **1993**, *101*, 229–234. https://doi.org/10.1111/j.1699-0463.1993.tb00105.x

122. Gooding, A.M.; Bastian, J.F.; Peterson, B.M.; Wilson, N.W. Safety and Efficacy of Intravenous Immunoglobulin Prophylaxis in Pediatric Head Trauma Patients: A Double-Blind Controlled Trial. *J. Crit. Care* **1993**, *8*, 212–216. https://doi.org/10.1016/0883-9441(93)90004-5
123. Bancalari, A.; Muñoz Pérez, T. Prolonged Intravenous Immunoglobulin Treatment in Very Low Birth Weight Infants with Late-Onset Sepsis. *Neonatal Pediatr. Med.* **2020**, *27*, 259–264. https://doi.org/10.3233/NPM-190259
