# Supplementary material for: Assessing Monoclonal and Polyclonal Antibodies in Sepsis and Septic Shock: A Systematic Review of Efficacy and Safety
Source: Int J Mol Sci. 2025 Sep 11;26(18):8859. doi: 10.3390/ijms26188859 (PMC12469444; doi:10.3390/ijms26188859)
Supplement: Supplementary file 1 [file ijms-26-08859-s001.zip › Supplementary material 4. GRADE Summary of Findings Table.pdf]

Summary of findings table

Monoclonal and polyclonal antibodies compared with placebo or standard care for sepsis and septic shock

Population: Adult patients with sepsis or septic shock
Setting: Hospital intensive care units and emergency departments (multinational studies, predominantly USA, Europe, Asia)
Intervention: Monoclonal antibodies (anti-TNF-α, anti-endotoxin, anti-complement, anti-PD-L1, anti-CD14, anti-adrenomedullin) or polyclonal antibodies (IVIG, IgM-enriched IVIG)
Comparison: Placebo or standard care

Outcomes	Anticipated absolute effects[1] (95% CI)	Relative effect (95% CI)	№ of participants (studies)	Certainty of the evidence (GRADE)	Comments
	**Risk with placebo or standard care**	**Risk with monoclonal and polyclonal antibodies**			
		**Range across studies:** 29.5 to 40.3 per 100 patients			
**All-cause mortality** *Follow-up: 28 days*	**Range across studies:** 30.8 to 42.8 per 100 patients	**Narrative synthesis findings:**<br>• Anti-TNF-α: No significant reduction in unselected patients<br>• Anti-endotoxin: No significant mortality benefit<br>• IgM-enriched IVIG: Mortality reduction of 10-58% vs placebo 36-75% in small studies<br>• Subgroup benefit in patients with elevated IL-6 or early septic shock	**Not pooled** *Substantial heterogeneity in interventions, populations, and sepsis definitions precluded meta-analysis*	**10,332 participants** (29 RCTs)<br><br>**Key studies:**<br>• Abraham 1998: 1,879<br>• Bone 1995: 1,102<br>• Abraham 1995: 994<br>• Angus 2000: 847<br>• Albertson 2003: 826	⊕⊕⊕⊖ **Moderate**[2,3]
**Organ dysfunction severity** *Assessed with: SOFA score changes* *Follow-up: 7-28 days*	**Baseline SOFA scores:** Range 8.5 to 12.0 across studies	**SOFA score improvements:**<br>• Mean reduction of 4.1 vs 2.0 points (p=0.01) in septic shock subgroups with targeted therapies<br>• Emerging precision strategies (anti-PD-L1, anti-C5a, anti-adrenomedullin) showed measurable SOFA improvements	**Not pooled** *Heterogeneous measurement timepoints and intervention types*	**Subset of participants from 29 RCTs**<br><br>**Studies reporting SOFA:**<br>• Bauer 2021: 72<br>• Laterre 2021: 301<br>• Morris 2012: 300<br>• Hotchkiss 2019: 35	⊕⊕⊖⊖ **Low**[2,3,4]
**Critical care resource utilization** *Assessed with: Vasopressor-free days, ICU-free days, ventilator-free days* *Follow-up: 14-28 days*	**Baseline organ support requirements:**<br>• Vasopressor use: 46-75% of patients<br>• Mechanical ventilation: 60-85% of patients	**Resource utilization improvements:**<br>• Vasopressor-free days: median improvement of 1-2 days<br>• Shock-free survival at day 14: 65% vs 55% (p=0.12)<br>• Time to shock reversal: 3-day reduction (median 4 vs 6 days, p=0.02)	**Not pooled** *Inconsistent outcome definitions and measurement timepoints*	**Subset of participants from 29 RCTs**<br><br>**Studies reporting resource outcomes:**<br>• Abraham 1998: 1,879<br>• Cohen 1996: 564<br>• Rice 2006: 621<br>• Panacek 2004: 634	⊕⊕⊖⊖ **Low to Moderate**[2,3,4]
**Biomarker modulation** *Assessed with: IL-6, TNF-α, HLA-DR, adrenomedullin, C5a levels* *Follow-up: 24 hours to 7 days*	**Baseline inflammatory markers:** Elevated across studies, variable reporting	**Biomarker changes:**<br>• Adrecizumab: Significant reduction in circulating bio-adrenomedullin levels<br>• Anti-TNF-α: Variable cytokine modulation<br>• Anti-C5a: Measurable complement pathway inhibition<br>• Anti-endotoxin: Inconsistent inflammatory marker changes	**Not pooled** *Heterogeneous biomarkers and measurement methods*	**Subset of participants from 29 RCTs**<br><br>**Studies with biomarker data:**<br>• Laterre 2021: 301<br>• Bauer 2021: 72<br>• Abraham 1998: 1,879<br>• Panacek 2004: 634	⊕⊕⊖⊖ **Low to Moderate**[2,3,4]
**Safety and tolerability** *Assessed with: Serious adverse events, infusion*	**Placebo adverse event rates:** 4-6% serious adverse events across studies	**Intervention adverse event rates:**<br>• Overall SAEs: 4.6% (similar to placebo)<br>• Infusion reactions: 5-	**Consistently favorable safety profile across all intervention types**	**10,332 participants** (29 RCTs)<br><br>**Safety data from all studies:**	⊕⊕⊕⊕ **High**

Outcomes	Anticipated absolute effects[1] (95% CI)	Relative effect (95% CI)	№ of participants (studies)	Certainty of the evidence (GRADE)	Comments
reactions, immunogenicity, secondary infections Follow-up: 28-90 days		15% (mild, transient)<br>• Immunogenicity: <5% (murine/chimeric), up to 40% (ovine fragments) without clinical consequences<br>• No increased secondary infections or organ toxicity		• Anti-TNF-α: 6,214 patients<br>• Anti-endotoxin: 3,202 patients<br>• Polyclonal IVIG: 793 patients<br>• Other targeted: 123 patients	

GRADE Working Group grades of evidence

High certainty: We are very confident that the true effect lies close to that of the estimate of the effect
Moderate certainty: We are moderately confident in the effect estimate; the true effect is likely to be close to the estimate of the effect, but there is a possibility that it is substantially different
Low certainty: Our confidence in the effect estimate is limited; the true effect may be substantially different from the estimate of the effect
Very low certainty: We have very little confidence in the effect estimate; the true effect is likely to be substantially different from the estimate of effect

Explanations

[1] **Anticipated absolute effects:** Based on observed control group risks across included studies. For systematic reviews without meta-analysis, ranges are provided rather than point estimates.

[2] **Downgraded for risk of bias:** Several trials (De Simone, Gallagher, Konrad, Toth) had high risk of bias in blinding of participants and outcome assessors.

[3] **Downgraded for inconsistency:** Substantial heterogeneity in antibody targets (TNF-α, endotoxin, complement, adrenomedullin), patient selection criteria, sepsis definitions (evolution from Bone 1992 to Sepsis-3), and outcome measurement methods across the 30-year study period.

[4] **Downgraded for imprecision:** Confidence intervals for secondary outcomes frequently crossed the line of no effect, with many studies underpowered for secondary endpoints. Wide variability in effect estimates across studies.

Clinical interpretation

For mortality (primary outcome): Current evidence does not support routine use of single-target monoclonal antibodies in unselected sepsis populations. IgM-enriched polyclonal immunoglobulin may reduce mortality when administered early, but evidence is limited to small studies. Biomarker-guided approaches show promise in selected subgroups.

For organ dysfunction: Precision antibody strategies targeting specific pathways (complement, checkpoint inhibition, adrenomedullin) may improve organ dysfunction scores, particularly in septic shock patients with appropriate biomarker profiles.

For resource utilization: Modest improvements in vasopressor requirements and shock resolution may occur with targeted approaches, though clinical significance requires further validation in larger studies.

For safety: All antibody interventions demonstrate excellent tolerability profiles, supporting continued investigation in appropriately selected patient populations.

Future research priorities: Implementation of rapid immune profiling, adaptive trial designs, combination regimens, and precision medicine approaches based on individual patient immune status and biomarker profiles.

Exact Participant Numbers

Total: 10,332 participants across 29 RCTs

Detailed breakdown by intervention type:

- **Anti-TNF-α:** 6,214 patients
- **Anti-endotoxin:** 3,202 patients
- **Polyclonal IVIG:** 793 patients
- **Other targeted:** 123 patients
